# Genome-wide gene expression in response to parasitoid attack in *Drosophila*

**DOI:** 10.1186/gb-2005-6-11-r94

**Published:** 2005-10-31

**Authors:** Bregje Wertheim, Alex R Kraaijeveld, Eugene Schuster, Eric Blanc, Meirion Hopkins, Scott D Pletcher, Michael R Strand, Linda Partridge, H Charles J Godfray

**Affiliations:** 1 Centre for Evolutionary Genomics, Department of Biology, University College London, Darwin Building, Gower Street, London WC1E 6BT, UK; 2 NERC Centre for Population Biology, Division of Biology, Imperial College London, Silwood Park Campus, Ascot, Berkshire SL5 7PY, UK; 3 European Bioinformatics Institute, Wellcome Trust Genome Campus, Hinxton, Cambridge CB10 1SD, UK; 4 Huffington Center on Aging and Molecular and Human Genetics, Baylor College of Medicine, One Baylor Plaza, Houston, TX 77030, USA; 5 Department of Entomology, 420 Biological Sciences, University of Georgia, Athens, GA 30602-2603, USA

## Abstract

**Background:**

Parasitoids are insect parasites whose larvae develop in the bodies of other insects. The main immune defense against parasitoids is encapsulation of the foreign body by blood cells, which subsequently often melanize. The capsule sequesters and kills the parasite. The molecular processes involved are still poorly understood, especially compared with insect humoral immunity.

**Results:**

We explored the transcriptional response to parasitoid attack in *Drosophila *larvae at nine time points following parasitism, hybridizing five biologic replicates per time point to whole-genome microarrays for both parasitized and control larvae. We found significantly different expression profiles for 159 probe sets (representing genes), and we classified them into 16 clusters based on patterns of co-expression. A series of functional annotations were nonrandomly associated with different clusters, including several involving immunity and related functions. We also identified nonrandom associations of transcription factor binding sites for three main regulators of innate immune responses (GATA/*srp*-like, NF-κB/*Rel*-like and Stat), as well as a novel putative binding site for an unknown transcription factor. The appearance or absence of candidate genes previously associated with insect immunity in our differentially expressed gene set was surveyed.

**Conclusion:**

Most genes that exhibited altered expression following parasitoid attack differed from those induced during antimicrobial immune responses, and had not previously been associated with defense. Applying bioinformatic techniques contributed toward a description of the encapsulation response as an integrated system, identifying putative regulators of co-expressed and functionally related genes. Genome-wide studies such as ours are a powerful first approach to investigating novel genes involved in invertebrate immunity.

## Abstract 2

Expression profiling of the transcriptional response at 9 time points of *Drosophila *larvae attacked by insect parasites revealed 159 genes that were differentially expressed between parasitized and control larvae. Most genes with altered expression following parasitoid attack had not previously been associated with immune defense.

## Background

*Drosophila melanogaster *is an important model organism for studying the mechanistic basis and evolution of immunity and pathogen defense. The two main classes of parasites against which it must defend itself in the wild are pathogenic microorganisms (bacteria, viruses, microsporidia and fungi) and parasitoids. Parasitoids are insects whose larvae develop by destructively feeding in (endoparasitoids) or on (ectoparasitoids) the bodies of other insects, eventually killing their hosts. They are ubiquitous in natural and agricultural ecosystems and can have major impacts on the population densities of their host, which makes them a valued agent for biocontrol. Most species that parasitize *Drosophila *are endoparasitic wasps (Hymenoptera) that attack the larval stage, or are species that feed externally on the pupae but inside the puparium. It is well known that host insects including *Drosophila *have evolved potent immunologic defense responses against parasitoid attack, and that parasitoids have evolved counter-strategies to subvert host defenses [1]. How these defense and counter-defense responses are regulated is not well understood, however. Here we report a microarray study of the transcriptional response of *Drosophila *to parasitoid attack. It is the first global expression analysis of the immunologic defense of a host insect against parasitoids, and aims to provide a comprehensive description of the timing and sequence of genes that signal during this innate immune response.

Like most animals, the innate immune response of *Drosophila *consists of both humoral and cellular defense mechanisms. Humoral defenses against bacterial and fungal infection have been intensely investigated over the past decade and are now relatively well understood [2,3]. These humoral defenses are activated when pathogen recognition molecules detect conserved surface molecules on microorganisms. This in turn activates the Toll and imd signaling pathways, which upregulate expression of antimicrobial peptides and many other genes [4,5]. Homologous signaling pathways regulate antimicrobial defense in other animals including vertebrates [6]. Cellular immune responses such as phagocytosis and nodule formation are also very important in defense against microorganisms [7]. The Janus kinase (JAK)/signal transducer and activator of transcription (STAT) pathway is closely involved in the cellular and humoral responses as well [8].

**Figure 1:**
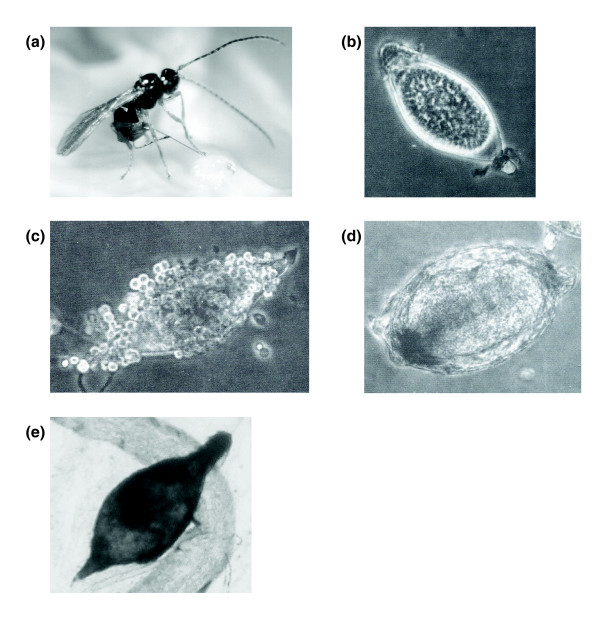
The *Drosophila *immune response after attack by parasitoids. **(a) **The parasitoid *Asobara tabida *stabs a second instar *Drosophila melanogaster *larvae with her ovipositor and inserts a single egg. **(b) **The parasitoid egg is susceptible to nonself recognition by membrane-bound and noncellular pattern recognition proteins in the larval hemolymph. **(c) **Hemocyte proliferation and differentiation is triggered, and the blood cells aggregate around the parasitoid egg. **(d) **The hemocytes form a multilayered capsule around the parasitoid egg and melanin is deposited on the capsule. **(e) **The parasitoid egg dies when it becomes fully melanized.

The chief invertebrate defense against macroparasites such as parasitoids is a cellular immune response called encapsulation (Figure 1) [1]. An encapsulation response begins when blood cells (hemocytes) recognize and bind to the foreign body. Additional hemocytes then adhere to the target and one another, which results in the formation of a capsule comprised of overlapping layers of cells. This response typically begins 4-6 hours after parasitism and is completed by about 48 hours [9]. Capsules often melanize, 24-72 hours after parasitism, and parasitoids are probably killed by asphyxiation or through necrotizing compounds associated with the melanization pathway [10,11].

In *Drosophila *larvae three types of mature hemocytes are recognized: plasmatocytes, lamellocytes and crystal cells. Plasmatocytes and crystal cells are present in the hemolymph of healthy larvae, whereas lamellocytes are only produced after attack by parasitoids [10-12]. Capsules consist primarily of lamellocytes, although crystal cells and plasmatocytes are present. Crystal cells also release phenoloxidase and possibly other factors that result in melanization of the capsule [13]. After parasitism the numbers of hemocytes increase via proliferation of cells in the hematopoietic organs (lymph glands) and hemocytes already in circulation. However, hematopoietic responses vary with the species of parasitoid and the stage of the host attacked [14-16]. The molecular basis for recognition of parasitoids is unknown, although experiments with mutant stocks implicate a number of signaling pathways (Toll, JAK/STAT and ras/raf/mitogen-activated protein kinase [MAPK]) in hemocyte proliferation and capsule formation [8,17,18].

Parasitoids have evolved several different strategies to overcome host immune responses [1]. Wasps in the genus *Asobara *(Braconidae) are important parasitoids of larvae of *Drosophila*, including *D. melanogaster*. They evade encapsulation by laying eggs that adhere to the fat body and other internal organs of the host [19,20]. This often results in incomplete formation of a capsule, which allows the parasitoid egg to hatch and escape encapsulation [9]. The parasitoid larva then suspends development while its host grows in size and only starts its destructive feeding during the host's pupal period. The growth of parasitized *Drosophila *larvae is normal until pupariation, irrespective of whether they successfully encapsulate the parasitoid, except that the investment in immune responses may incur slight delays in their speed of development [21,22]. The fraction of *D. melanogaster *surviving parasitism varies with larval age at the time of attack, temperature, geographic strain and parasitoid species [9,23]. *D. melanogaster *can also be selected in the laboratory for increased resistance to its parasitoids. For example, five generations of selection for resistance against *Asobara tabida *increased the frequency of larvae that successfully encapsulated parasitoid eggs from about 5% to about 60% [24,25]. Increased resistance was associated with higher densities of circulating hemocytes, but also reduced larval competitiveness [26]. There are also differences in the degree to which different *Drosophila *spp. can defend themselves against parasitism, and this too appears to be correlated with hemocyte densities [27].

Previous genome-wide studies of *Drosophila *immunity all investigated responses against microbial pathogens [28-34]. Defenses against macroparasites such as parasitoids are likely to be very different, and their study, like that of responses to microbial pathogens, may reveal conserved components of the innate immune system. As a first step toward unraveling the genetic control of defenses against parasitoids, we designed a large-scale experiment to monitor the involvement and timing of differentially expressed genes during the entire immune response. We used the Affymetrix *Drosophila *Genome 1 Array chip (Affymetrix, Santa Clara, CA, USA) to study the transcriptional response of *D. melanogaster *to attack by *A. tabida*. Larvae of a Southern European strain of fly that is partially resistant to this parasitoid were exposed to parasitoid attack and then RNA was harvested at nine subsequent time points (from 10 minutes to 72 hours) and compared with RNA from control larvae of the same age. We used bioinformatic techniques to look for patterns of co-expression and for shared regulatory sequences. We also used current knowledge of the molecular basis of defense against parasitoids to identify a set of candidate genes and molecular systems that might be involved in defense against parasitoids, and explored whether they were present in our transcription set.

Comparison with previous studies revealed many differences in gene expression patterns between the antimicrobial and antiparasitoid responses, and implicated several new genes in insect immunity. Clusters of co-expressed genes were identified that we believe may be functional related components of the immune response (for example, a series of serpins and serine-type endopeptidases that may be involved in a proteolytic cascade). We identified a putative transcription factor binding site motif that has not hitherto been linked to any known transcription factor. The transcription factor binding sites of three known regulators of immunity were strongly associated with several clusters of co-expressed genes. Some genes known to be involved in encapsulation were identified in our screens whereas others were not, indicating that they are post-transcriptionally regulated.

Our work increases our understanding of the immunologic defense responses in hosts to parasitoid attack, and paves the way for further experiments to investigate the roles of genes and pathways of particular interest. It suggests a variety of new approaches to understanding the encapsulation process and should help us to move toward a systems level description of innate immunity in insects.

## Results

**Figure 2:**
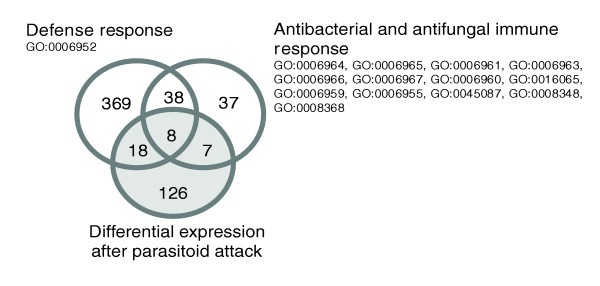
Venn diagrams of genes that changed expression after parasitoid attack and known immunity genes. The differentially expressed genes after parasitoid attack differed largely from those with a GeneOntology (GO) annotation for immunity or defense (GO database September 2004; the GO codes are also shown in the figure). Some of the probe sets in our set matched to multiple genes (see additional data files), thus reporting on the expression of potentially all of these genes. We included the multiple gene annotations per probe set to define our set of differentially expressed genes for the comparisons.

The expression profiles of 159 probe sets differed significantly between parasitized and control larvae. Because we accepted a 1% false discovery rate (see Materials and methods, below), a small number of these probe sets (probably one or two) could have been incorrectly identified. Our assignment of genes to these probe sets, and the functional and structural annotation of these genes are provided in Additional data file 1. Note that some probe sets matched more than one gene (see Materials and methods, below) and some genes are represented by more than one probe set; thus, there are sometimes differences between (sub)totals or percentages calculated for probe sets and genes. Of all the differentially expressed genes, 55% had some information on 'molecular function', 55% on 'biologic process', and 46% on both in the GeneOntology database. For 59 genes (37%) there was no functional annotation in GeneOntology. These percentages did not differ significantly from their equivalents calculated for the full set of genes represented on the Affymetrix *Drosophila *microarray (*P *> 0.05, Fisher exact test). Thirty-three genes had GeneOntology annotations that included immunity and defense functions, which, as expected, was significantly more than expected by chance (*P *< 0.001, EASE analysis). However, more than 80% of the differentially expressed genes had not previously been associated with an immune or defense response in GeneOntology, whereas many known immunity genes were not differentially expressed (Figure 2).

### Patterns of co-expression

**Figure 3:**
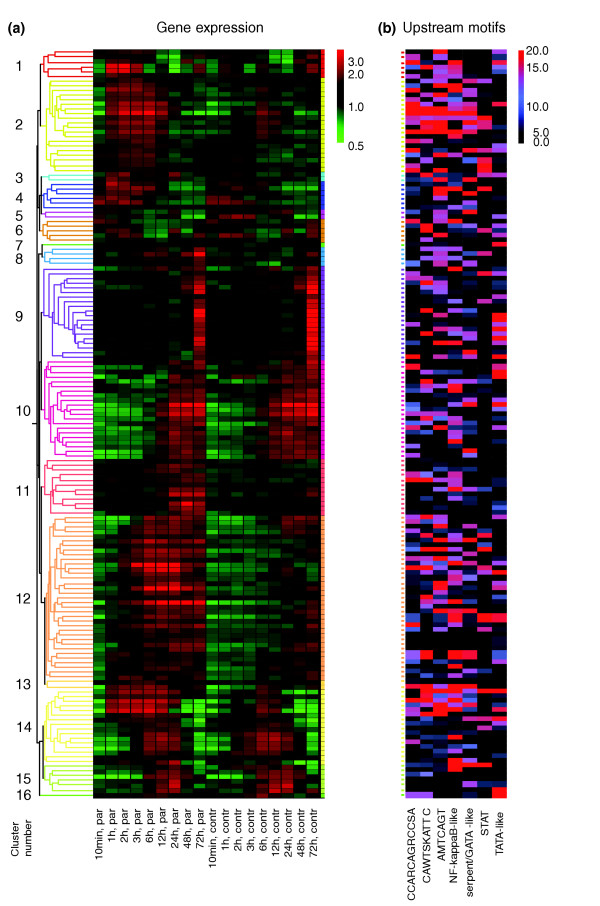
Gene expression levels and distribution of regulatory motifs for the genes differentially expressed after parasitoid attack. **(a) **Expression levels for genes (rows) at different sample time points (columns: 1-9 parasitized larvae; 10-18 unparasitized larvae). The expression levels are given as multiples of the median for that gene, using a color code illustrated at top right. At the left the dendrogram produced by the clustering algorithm is shown, with the 16 clusters discussed in the text depicted in different colors (with their code numbers; the final column on the right shows the clusters again using the same color key). **(b) **The distribution of putative regulatory motifs in the -1,000 to +50 base pair upstream regions of the genes. The colors indicate the number and strength of the matches for each motif (see code on upper right, in which a score of 0 is equivalent to no matches, 10 is equivalent to one strong or two weak matches, and 20 is equivalent to multiple strong matches).

**Figure 4:**
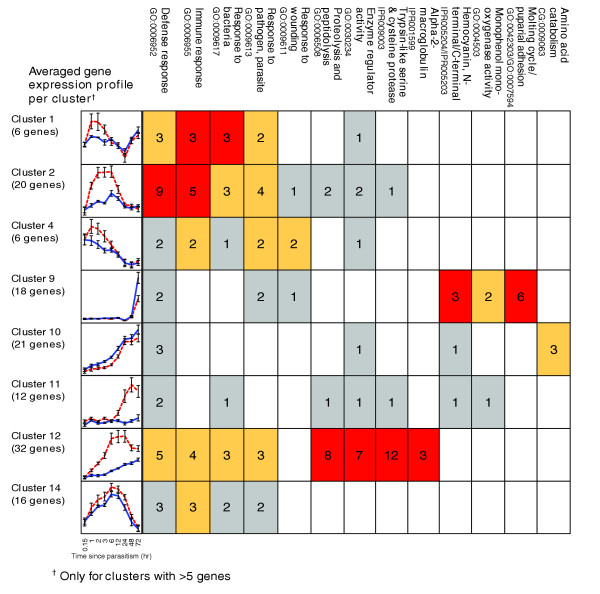
Gene expression profiles and functional annotations for the eight largest clusters of co-expressed genes. On the left-hand side the average expression levels for the genes in the eight clusters are shown (log_2_-transformed expression values, divided by the median for that gene across all time points and treatments). Dashed lines represent parasitized and unbroken lines represent unparasitized larvae, and the bars indicate standard errors. Functional annotations associated with clusters are shown along the top, and colors in the matrix indicate the strength of association (yellow = Ease scores (see text) <0.05; red = after Bonferroni correction at *P *< 0.05; grey = at least one gene with this annotation). The full annotation for all probe sets is provided in Additional data file 1.

The pattern of expression of the 159 probe sets that responded to parasitoid attack is shown in Figure 3a. The clustering algorithm sorted the probe sets into a gene tree, from which we defined 16 clusters that varied in size from one to 35 probe sets. Of these clusters, seven contained five or fewer genes, and because of this there is low statistical power to detect over-represented annotation terms. However, 83% of the probe sets were placed in eight clusters that each included more than five genes. The mean expression profile of genes in these clusters, as well as the GeneOntology annotation terms that were significantly over-represented, are shown in Figure 4; the individual gene expression profiles and the full details of the annotation are provided in Additional data files 1 and 2.

In six of these clusters (clusters 1, 2, 4, 11, 12 and 14 in Figure 4; 92 genes in all) the genes tended to have higher expression levels in parasitized than in control larvae, whereas in the remaining two (clusters 9 and 10; 39 genes) the reverse pattern was found. The clustering algorithm uses information from both temporal changes in expression and differences between treatment and control. The clusters with upregulated genes in parasitized larvae fall into a group in which the genes tend to be expressed more strongly for 3-6 hours after parasitism before returning to the same levels as controls (clusters 1, 2 and 4; 32 genes) and one in which the greatest differences occur 6-72 hours after parasitism (clusters 11 and 12; 44 genes), with the genes in the remaining more heterogeneous cluster 14 (16 genes) tending to be differentially expressed at some of the intermediate time points. Of the two clusters of downregulated genes, cluster 10 (21 genes) is largely defined by reduced expression levels in parasitized larvae throughout the course of the experiment, whereas cluster 9 (18 genes) contains genes that are expressed at the end of the experiment, and more strongly in control larvae.

We found highly significant over-representation of annotation terms in four clusters. Half of the genes in cluster 1 (six genes), which were expressed within 1-3 hours of parasitism, are annotated as involved in both immune response and response to bacteria. They included the two antimicrobial peptides *AttA *and *AttB*. Cluster 2 (20 genes) had highly significant over-representation of the category immune response (five genes: *CG15066*, *nec*, *Mtk*, *hop*, *dome*) and of its parent category defense response (including a further four genes: *IM1*, *IM2*, *CG13422*, *CG3066*).

Cluster 12 (32 genes) contained a highly significant over-representation of genes for the GeneOntology terms proteolysis and peptidolysis (eight genes) and enzyme regulator activity (seven genes), and the InterPro terms peptidase, trypsin-like serine and cysteine proteases (12 genes), as well as proteins with putative α_2_-macroglobulin domains (three genes), which may be involved in protease inhibition. These genes are upregulated relative to controls, in particular between 6 and 24 hours after parasitism. Their annotations suggest that they may be involved in a proteolytic cascade that might regulate part of the immune response, such as the formation of the melanized capsule. This hypothesis is supported by the occurrence of clip domains, which enable activation of proteinase zymogens, in several of the serine-type endopeptidases (*CG16705*, *CG11313*, *CG3505*).

Finally, cluster 9 contained a highly significant over-representation of genes with the GeneOntology annotations molting cycle and puparial adhesion (six genes) and the InterPro terms hemocyanin (N-terminal and C-terminal; three genes). This cluster comprises genes expressed at 72 hours after parasitism, by which time the third-instar larva is preparing to pupate; hence, the appearance of genes associated with molting and pupariation is not surprising. What is more interesting is the relatively reduced expression of these genes in parasitized larvae. Even hosts that have successfully been parasitized pupate (the parasitoid emerges from the puparium) and the low expression probably reflects delayed development caused by parasitism. Two of the genes with hemocyanin domains have monophenol mono-oxygenase activity (*CG8193*, *Bc*), and the latter of these has been associated with the melanization stage of encapsulation. In our assay, however, the expression profile suggests a closer involvement in pupation than in capsule melanization.

### Regulatory sequences

Our analysis identified a set of six putative transcription factor DNA-binding motifs (TFBMs) that were significantly associated with genes in the different clusters. To these we added the STAT motif, which did not quite meet all of our criteria but which is known to be involved in the encapsulation response [8]. The pattern of association of these seven motifs is shown in Figure 3b. Three of the six putative TFBMs matched sequences associated with known transcription factors: *serpent *and related GATA-factors, *Relish *and similar nuclear factor-κB (NF-κB) factors, and TATA transcription factors. Both *serpent *and *Relish *were previously associated with the *Drosophila *immune response [35,36] and *serpent *with hematopoiesis [37].

Table 1 shows in which clusters and at which times the seven TFBMs are most strongly over-represented, and detailed quantitative information is provided in Additional data files 2, 3, 4. We found strong associations between the *serpent*/GATA-type motifs and the genes in cluster 2, many of which had been annotated as being involved in immunity, and the *Relish*/NF-κB-type motifs and the genes in cluster 12 associated with proteolysis and peptidolysis. A number of genes that shared the *Relish*/NF-κB-like binding site motif are all located in a cluster on the 2R chromosome (*IM1*, *IM2*, *CG15065*, *CG15066*, *CG15067*, *CG15068*, *CG16836*, *CG16844*, *CG18107*). The single most significant association, however, was with the motif CCARCAGRCCSA (using IUPAC Ambiguous DNA Characters [38]), which has not hitherto been associated with any transcription factor. It was found to be particularly often associated with genes in clusters 2 and 12, both upstream and in the first 50 base pairs after the start codon.

We tested whether the genes for the transcription factors associated with the TFBMs were themselves upregulated or downregulated after parasitoid attack. The NF-κB-like factor *Relish *was significantly upregulated 1 hour after parasitism before returning to the same levels as controls. There was no evidence of changed expression for *serpent *or any of the other GATA-like factors, *Stat92E*, or TATA factors. Interestingly, *serpent*/GATA-type motifs were found to be over-represented in clusters 1, 2 and 12 (upregulated genes that tend to be associated with immunity) as well as in clusters 9 and 10 (downregulated genes that tend to be associated with development and metabolism). The lack of differential expression of this transcription factor might thus be explained by it being present in both parasitized and unparasitized larvae but performing different functions.

### Candidate genes

**Figure 5:**
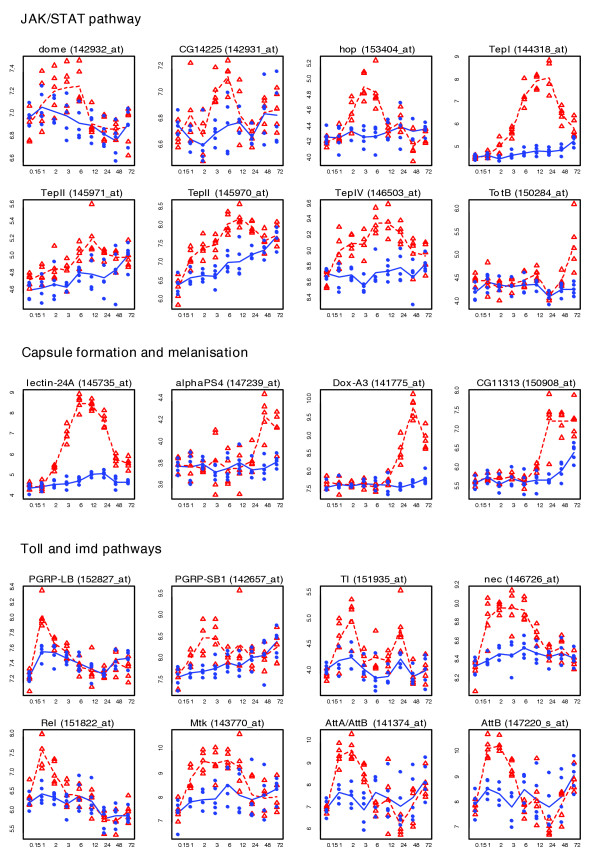
Expression profiles of genes from pathways and processes known to be involved in immunity. Each graph depicts the log_2 _expression values for a single gene at different time points (in hours) after parasitoid attack. The blue circles and red triangles show the individual replicates of the control and parasitized larvae, respectively. The lines denote the average expression at each time point. See text for a discussion of the selected genes.

We explored whether a variety of genes known to be involved in the response to parasitoid attack had differential patterns of expression. In particular, we looked for genes associated with hemocyte proliferation and differentiation; cellular defense, in particular capsule formation and melanization; and the humoral response to microorganism infection and in regulating coagulation and melanization (Table 2). The gene expression profiles of a selection of candidate genes that were differentially expressed are shown in Figure 5. The expression profiles of all differentially expressed genes are provided in Additional data file 2.

The most dramatic initial response to parasitoid infection involves proliferation of hemocytes and differentiation of lamellocytes in the larval lymph glands, and recent work has shown that this involves the Toll and the JAK/STAT signaling pathways, which are both also implicated in responses to microorganism infection [8,39]. Activation of the Toll pathway in the lymph glands results in hemocyte proliferation, whereas in the fat body it results in the transcription of antimicrobial peptides [39]. Because relatively little is known about this pathway in the lymph glands, we discuss the Toll pathway in relation to its antimicrobial humoral response (see below). The *os *and *Upd*-like genes for the ligands that activate the JAK/STAT pathway in flies were not differentially expressed in our assay. The receptor *dome *and a similar but shortened version of this receptor, *CG14225*, as well as the *Drosophila *Jak *hop*, were all significantly upregulated 2-6 hours after attack. The transcription factor Stat92E (for discussion of the STAT TFBM, see above) is associated with proteins in the Tep and Tot families, whose functions are involved respectively in enzyme regulation and severe stress responses [8]. The genes *TepI*, *TepII*, *TepIV *and *TotB *were differentially expressed after attack by parasitoids (with the peak of expression later than *dome *and *hop*), whereas *TotM *and *TepIII *were not. The other Tot genes (including the best characterized *TotA *[40]) were not represented on the Affymetrix *Drosophila *Genome 1 Array. The JAK/STAT pathway is also thought to crosstalk with the ras/raf/MAPK pathway during hemocyte proliferation [41], but no genes associated with the latter were significantly affected by parasitism.

The encapsulation process that results in the death of the parasitoid egg involves cell adhesion and melanization [9]. Lectins and integrins are two important classes of protein that mediate cell adhesion in immune responses [42]. The gene *lectin-24A *was massively upregulated in parasitized larvae 6-48 hours after parasitization at the time when the capsule is formed (10-fold to 16-fold at the peak of expression). Lectins can function as adhesion ligands for invertebrate hemocytes [42]. The gene *αPS4*, which encodes an α integrin subunit, was upregulated at 48-72 hours, at about the time when the multilayered capsules are completed and melanization occurs [9]. Also at this time, a gene for an immunoglobulin-like protein with haemocyanin domains and predicted monophenol mono-oxygenenase activity (*Dox-A3*), and a serine-type endopeptidase with predicted monophenol mono-oxygenase activator activity (*CG11313*) were upregulated. Both are likely to be involved in melanin deposition. Two other genes in our list encode proteins with predicted monophenol mono-oxygenase activity (*Bc*, *CG8193*), but the expression profiles of these genes suggested a role in pupation and/or metamorphosis rather than in melanization.

The *Drosophila *response to microorganism attack involves, among others, the production of antimicrobial peptides controlled by the Toll and imd pathways [43]. The primary receptors are peptidoglycan recognition proteins that bind specifically to different classes of microorganism, and of the 13 genes of this family known in *Drosophila *two were significantly upregulated (*PGRP-LB*, *PGRP-SB1*). However, only a few components of the Toll (*Tl *and *nec*) and imd (*Rel*) pathways were significantly upregulated in response to parasitoid attack. Out of the 14 antimicrobial peptides known in *Drosophila*, only three (*Mtk*, *AttA *and *AttB*) showed significantly increased expression in parasitized larvae. The first of these acts against filamentous fungi and Gram-positive bacteria, and the latter two against Gram-negative bacteria [44]. All of these genes showed their greatest relative increase in expression soon after parasitism. Parasitoid attack involves wounding and penetration, and it is possible that the production of antimicrobial peptides is associated with damage to the exoskeleton and low-level exposure to microbial factors on the surface of the fly larvae or ovipositor of the wasp.

## Discussion

We still have a relatively poor understanding of the genetic mechanisms that underlie host defense to parasitoid attack, despite the immense importance of parasitoids to the population dynamics and control of many insects. A full understanding will require extensive experimental investigation, but we believe that the dataset described here provides a first and important step toward unraveling the genes and pathways involved and their sequence of action.

We investigated the transcriptional profile of *D. melanogaster *larvae during the 72 hours after they had been parasitized by *A. tabida*. The *Drosophila *strain we used was highly immunocompetent and was able to encapsulate about 75% of parasitoid eggs. Furthermore, the counter-resistance strategy of the parasitoid species we used is thought to consist of evasion rather than manipulation of host defenses [19,20]. We were thus able to study a strong and uninterrupted defense response to parasitoid attack. The 72-hour period we studied, which covers the full immune response from detection of the parasitoid egg to completion of the capsule, lasts from the late second instar to just before pupation, which is just over half the length of the host's total larval stage. As expected, a very large number of genes exhibited differences in expression over time (over 8,000 genes with a 1% false discovery rate). A much more restricted set of genes (represented by 159 probe sets) differed significantly in their transcription profiles between the control and parasitized groups. We analyzed patterns of co-expression and shared regulatory motifs within this set of genes, and then asked whether they encoded proteins previously associated with defenses involved in the response to parasitoid attack. The majority of differentially expressed genes in our study had not previously been associated with innate immunity, which is a reflection of the substantial differences in immunologic responses to pathogens and macro-parasites.

Based on our clustering algorithm we identified 16 clusters, the eight largest of which contained 83% of the probe sets. Six included genes (70%) that tended to be more highly expressed in parasitized larvae, whereas two contained genes (30%) that tended to be more highly expressed in nonparasitized larvae. Not all clusters had a clear temporal signature, but we identified groups of genes expressed during the first few hours after parasitoid attack and then later at the time of capsule formation. One cluster contained genes that had reduced levels of expression in parasitized larvae only at the final sampling point, 72 hours after attack. The genes in almost all clusters exhibited significant changes in expression through time in both parasitized and control larvae, which reinforces the importance of having controls of the same age rather than comparing transcription profiles before and after parasitoid attack. Moreover, it indicates that most genes are not exclusively involved in immunity and defense, but also in other processes while the fly larva grows and readies for pupation.

We annotated all the genes in each cluster and then tested statistically for over-represented GeneOntology and InterPro terms. For relatively small clusters of genes, as present here, this procedure does not have very great statistical power, yet we were able to associate potential functions with four clusters: (i) two clusters of genes expressed soon after parasitoid attack were associated with immune functions, (ii) a cluster of genes that were expressed later after parasitism was associated with functions involved in proteolytic cascades, and (iii) the cluster of reduced-expression genes at 72 hrs was associated with preparation for pupation. The first two observations are consistent with an initial "front-line" reaction to parasite challenge, followed by a slower response, perhaps involving the consolidation of the capsule. The last observation is probably a reflection of another consequence of parasitism, a reduction in the rate of development, perhaps a cost of mounting the defensive response [21,22]. At the last sampling point unparasitized larvae were further developed and had begun to express genes associated with pupation.

Our search for potential TFBMs identified six potential sequences, three of which represented already well known transcription factors. The most significant sequence, CCARCAGRCCSA, was not associated with a currently recognized factor, and might represent a new regulatory mechanism involving a novel transcription factor. To screen for such a transcription factor, one could use a yeast 1-hybrid system and protein purification with affinity columns. Interestingly, two clusters of relatively highly expressed genes with significant annotation associations also had strong associations with TFBMs; an immune-related cluster and the possible regulatory-cascade cluster were both significantly associated with *serpent*/GATA-type motifs, *Relish*/NF-κB-like motifs, the STAT motif, and the novel sequence just discussed. The transcription factor *Rel *itself was significantly upregulated immediately after parasitism, but not any of the other transcription factors identified in our screen. These data contribute toward a description of the encapsulation response as an integrated system rather than a simple collection of individual genes.

A number of biochemical systems and signaling pathways are known to be involved in the response to parasitoid attack or the formation of melanotic capsules. The JAK/STAT and Toll pathways have been implicated in regulating hemocyte proliferation. Several components of these signaling pathways, as well as a number of target genes they regulate, exhibited significantly increased expression levels in parasitized larvae compared with controls. We hypothesize that upregulated expression of lectins and integrins, and genes with functions associated with melanin deposition are involved in capsule formation. The Toll and imd pathways have a well known association with microbial defense, and Toll has also been implicated in regulating immune responses toward macro-parasites [18]. Two peptidoglycan recognition proteins and three antimicrobial peptides were significantly upregulated soon after parasitism. Because parasitoid attack involves puncturing the body wall, with the obvious possibility of microbial infection, we suggest that upregulation of these genes reflects low level exposure to microorganisms at parasitoid oviposition. Overall, however, parasitism by *A. tabida *induced relatively few changes in expression of antimicrobial effector genes under Toll and imd pathway control.

As with other microarray studies, there are limitations to what our work can tell us about the *Drosophila *response to parasitoid attack. Although the Affymetrix *Drosophila *Genome 1 Array chip contains a large fraction of *Drosophila *genes, about 8.5% are missing and so cannot be included in any analysis. More seriously, much of the response to parasitoid attack likely does not involve *de novo *gene expression but post-transcriptional and translational events. This may be particularly true of any initial, rapid response to parasitoid attack, where any delay in protein synthesis would be maladaptive. Several genes previously implicated in melanization were not differentially expressed, which also indicates the importance of post-transcriptional and post-translational regulation of gene expression. Finally, there is always the danger of false-positives in testing numerous hypotheses simultaneously. Fortunately, because of the large number of microarrays used in this study, we had relatively high statistical power, and we corrected for multiple hypothesis testing using Storey's false discovery rate method. This meant that of the 159 probe sets we identified for further study, we estimate that only one or two are likely to have been erroneously included.

In interpreting our results, two further more specific issues must be considered. First, with the combination of host and parasitoid strains used here, we estimate that about three-quarters of the flies parasitized in the experiment will mount a successful immune response and survive parasitism, but that about a quarter will succumb. Some hosts fail to encapsulate completely the parasitoid egg because it is partially embedded in host tissue. However, parasitized host larvae almost always show some signs of capsule formation and melanization, irrespective of whether they succeed in killing the parasitoid egg (unpublished data). This suggests that much of the transcriptional response to parasitoid attack will be the same in hosts that will or will not survive, although we cannot exclude the possibility that especially some of the later differences in gene expression are pathologic responses to parasitoid attack.

Second, it was not feasible to dissect out the parasitoid eggs from the larvae. Were we to have done this in live larvae, it would have resulted in changes in gene expression due to the major trauma involved, whereas in frozen larvae the eggs become firmly attached to larval host tissue and are very difficult to remove. It is thus possible that there might have been cross-reactivity between parasitoid transcripts and the probe sets on the microarray. However, we think this unlikely, both because the volume of RNA in the parasitoid egg is small compared with that in the host larvae, and because the specificity of the probes means that they are unlikely to cross-react with nucleic acid from an insect as evolutionarily distant as a hymenopterous wasp. The high specificity of the probes was substantiated when we blasted the sequences of the 159 *Drosophila *probe sets from our study to the genome scaffold of honeybee (*Apis melifera*, another hymenopteran). Over 75% of the probe sets gave no match at all, and those in the remaining probe sets were very poor (one or two probes per probe set, with at least three errors to the perfect match (PM) sequences).

Microarrays have been used to study the transcription profile of *Drosophila *adults or cells subject to attack by microbial pathogens. DeGregorio [5,30], Irving [28] and Boutros [32] and their coworkers challenged flies by wounding them with needles dipped in suspensions of bacteria or by shaking them with spores of the pathogenic fungus *Beauveria bassiana*. Roxström-Linquist and colleagues [31] compared the transcription profiles of adult flies orally infected by bacteria, microsporidia (*Octosporea muscaedomesticae*) and *Drosophila *C virus per os, or through shaking them with *Beauveria *spores. Irving [34] and Johansson [33] and their coworkers recently measured gene expression at 5-6 hours after microbial infection in, respectively, the hemocytes of third-instar larvae and a hemocyte-like cell line of *Drosophila*. Overall, 43% of the genes in our study appeared in one or more of the lists of genes identified as being involved in immunity in the microbial pathogen studies in adults, and only 8-10% of the genes in our study were also listed as upregulated or downregulated in the studies of cells. The overlap with individual studies was low, ranging from 8% to 32%. The genes that did consistently appear in the antimicrobial studies were predominantly those in the Toll and imd pathways, and some of the serine-type endopeptidases. However, the signaling in the Toll and imd pathways in response to parasitoid attack was atypical compared to the antimicrobial response, with the expression of many intracellular signaling elements and effector genes remaining unaffected. Thus, although there appears to be limited overlap, the innate humoral response to microorganisms and the innate cellular response to macroparasites are substantially different.

Irving and coworkers [34] also explored the transcriptional profile of larval hemocytes from mutant stocks differing in the abundances of plasmatocytes, crystal cells, and lamellocytes. Interestingly, some of the genes we identified as upregulated after parasitoid attack (for example, the integrin *αPS4*, the monophenol monooxygenase *Dox-A3 *and the G-protein coupled receptor *mthl2*) were associated in their study with the presence of lamellocytes, specialized hemocytes that are involved in capsule formation.

Genes involved in immunity against microbial pathogens and parasites have also been studied in genome-wide screens of the mosquito *Anopheles gambiae*, which is one of the main vectors of the human malaria parasite *Plasmodium *[45]. The *Anopheles *genome contains families of immunity genes that are partly orthologous to those in *Drosophila *[46]. Mosquitoes can mount a melanotic encapsulation response against the ookinete stage of *Plasmodium *in the insect's gut. This kills the parasite and disrupts the transmission cycle [47,48]. In contrast to the cellular encapsulation response by *Drosophila*, the melanotic encapsulations of the single-celled malaria parasites by *Anopheles *do not contain hemocytes and result from a humoral melanization of the ookinete [49,50] Gene silencing studies in the mosquito revealed that two C-type lectins and a leucine rich-repeat immunity protein were pivotal in the melanization response, with the former two averting melanization and the latter inducing it [51]. Parasitoid attack induced strong upregulation of a gene encoding a C-type lectin (*lectin-24A*) and the slight downregulation of a leucine-rich repeat gene (*Pxn*).

Compared with our results from *Drosophila*, there is a greater overlap in *Anopheles *between genes involved in microbial challenge and parasite infection [45]. A probable explanation for this is the difference between *Plasmodium *and parasitoids as targets for the immune system. Mosquito immunity against *Plasmodium *is mostly a noncellular response [49,50], and indeed there is evidence in *Anopheles *for pattern recognition receptors that both respond to bacteria and *Plasmodium *[46,52]. In addition, the natural history of the infection is different, with *Plasmodium *having a variable but relatively minor affect on mosquito fitness [53], whereas the parasitoid is invariably fatal if it is not destroyed. There may also be differences between the defense response of larval and adult insects.

Previous work has shown that there are geographic clines in the degree to which *Drosophila melanogaster *can defend itself against *Asobara tabida *[23], and that it is possible to artificially select *D. melanogaster *for enhanced resistance against this parasitoid [24]. Orr and Irving [54] demonstrated that differences in parasitoid resistance between several field populations were largely restricted to genes on chromosome 2. Three of our clusters with upregulated expression in parasitized larvae contained significantly more genes located on chromosome 2 than expected by chance (for clusters 2, 4 and 12, χ^2 ^with five degrees of freedom: *P *< 0.05) and, more specifically, a significant over-representation of differentially expressed genes located at chromosomal band 55C (EASE analysis: *P *< 0.001). Previous studies have suggested the occurrence of two loci in this region that might be related to parasitoid resistance, although the genes at these loci await further characterization [55]. An interesting evolutionary question is whether the differences in resistance, both geographical and before and after selection, are reflected in changes in transcription profile, and whether the genes involved are the same as those identified in the present study. Much evolutionary theory of host-parasite interactions predicts complex dynamics of alleles at loci involved in host defense, but has proved hard to test in the absence of firm information about the genes involved. Microarray studies offer a valuable tool for identifying these genes and making progress on this question.

*Drosophila *are attacked by several groups of parasitoids in addition to *A. tabida *and its relatives. In particular, parasitoids in the genus *Leptopilina *(Figitidae = Eucoilidae) have widespread distributions and can cause high levels of mortality in field populations of *Drosophila *[56-58]. *Leptopilina boulardi *is more specialized than *A. tabida *and exclusively parasitizes species of the *melanogaster *group. Artificial selection experiments showed that enhanced resistance to *L. boulardi *(increasing from about 0.5% to about 45% over five generations) also confers better resistance to *A. tabida *but not *vice versa *[59]. *Leptopilina *spp. have evolved a very different strategy to overcome the host immune response compared with that of *A. tabida*. At oviposition virus-like particles from the long (or venom) gland are injected into the host, and disrupt the immune system by altering hemocyte function [15,60,61]. Comparative microarray studies of flies exposed to the two parasitoids might help to explain the asymmetric cross-resistance and may also tell us whether the apparently very different counter-resistance mechanisms of *Asobara *and *Leptopilina *are reflected in different responses to parasitism by the host. Comparative microarray studies may also help to explain the curious observation that some species of *Drosophila *(*D. subobscura *is the best known example) never mount a defense response against a parasitoid egg, despite suffering high levels of attack in the field [62]. Finally, the strong selection pressure found in parasitoid-host interactions, in which one of the two participants invariably perishes, has resulted in a wide diversity of defense and counter-defense strategies in different species [1]. Comparative gene expression profiling of different parasitoid-host systems may help to reveal the unique and shared processes that underlie these defense and counter-defense strategies.

## Conclusion

**Figure 6:**
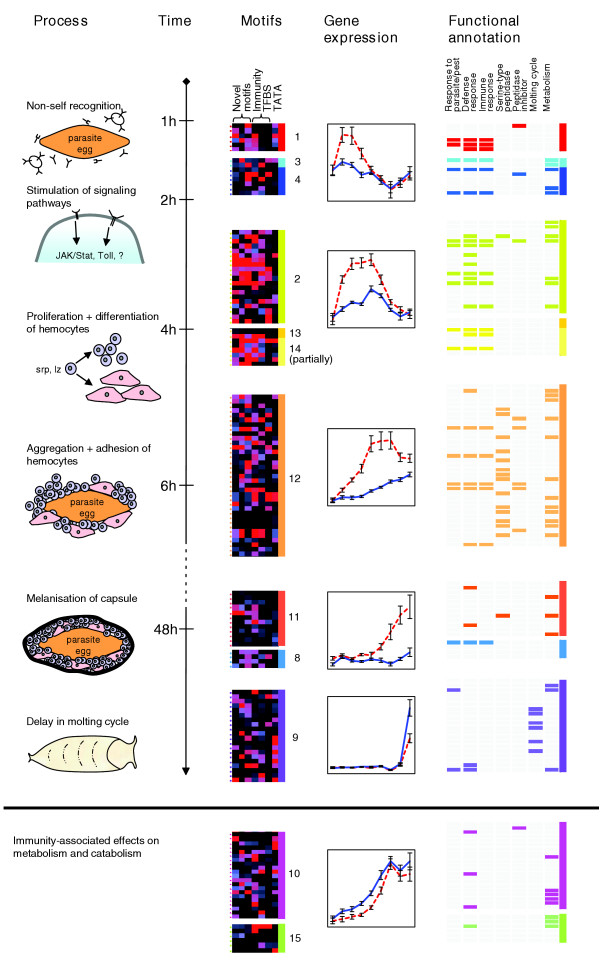
Overview and summary of our findings. The two left-hand columns show the time elapsed since parasitoid attack and a diagrammatic summary of major cellular and metabolic consequences of parasitism. The three right hand columns show the results of this study and the gene clusters that we hypothesize are associated with the different processes sketched on the far left. These three columns show the following: over-represented transcription factor binding motifs arranged by cluster (with code number) ordered by their time of maximum expression; average expression profiles of genes in these clusters (parasitized larvae in red, unparasitized larvae in blue) with marked temporal restricted expression; and functional annotations associated with genes in these clusters, in the same order as in the first of the three columns. A group of genes with relatively constant levels of reduced expression in parasitized larvae is shown separately at the bottom.

We believe that this is the first genome-wide study of the immune response of a host insect to attack by a parasitoid. Our study is relatively unusual in that we used 90 microarrays to produce a highly replicated and densely sampled time series in order to study the events that follow parasitoid attack. In Figure 6 we summarize our results and compare the expression profiles, functional annotations, and transcription factor binding motifs of the major gene clusters we identified. Different groups of co-expressed genes are associated with distinct phases of the response to parasitism identified by morphologic and previous molecular studies. We believe that further investigation of the genes identified here will help us to understand invertebrate cellular defense. Most genes whose expression changed in response to parasitoid attack differed from those induced during the antimicrobial immune response, and had not previously been associated with immunity and defense functions. We applied a combination of bioinformatic techniques to analyze our data, which contributed toward a description of the encapsulation response as an integrated system, identifying putative regulators of co-expressed and functionally related genes.

Parasitoids are major sources of mortality for *Drosophila *as well as many other types of insects. They are also of significant economic importance as biocontrol agents, and largely because of this the physiology of defense against parasitoids has been intensively studied for many years. Genome-wide expression studies such as ours provide a uniquely powerful approach to investigating new genes involved in invertebrate immunity and will complement these earlier approaches. Much current molecular work on insect immunity has concentrated on the humoral response to microorganisms, and our molecular understanding of cellular immunity is not as well developed. Improving the latter is important if we are to achieve a more balanced appreciation of how insects defend themselves from pathogens and parasites. Invertebrates do not have an adaptive immune system, as in vertebrates, but elements of the innate immune system are strongly conserved across the two groups of animals [6,63,64]. This is clearly so for the humoral immune response, but recent work has revealed unexpected homologies involving components of cellular innate immunity [65,66]. A better understanding of cellular defense in *Drosophila *thus may also be useful in the investigation of topics such as vertebrate lymphopoiesis and hematopoiesis.

## Materials and methods

### Insect strains

*Drosophila melanogaster *used in the study were collected in Avigliano, Italy, in July 2001, and were subsequently cultured in the laboratory on yeast-sugar *Drosophila *medium [25], at 20°C under a 16:8 light:dark cycle. The parasitoid strain was originally collected in Sospel, France and had been maintained in the laboratory for over 20 years on *D. subobscura*. On average, 73% of Sospel parasitoid eggs were successfully encapsulated by our experimental strain of fly.

### Collection of parasitized and control hosts

A single parasitoid was observed searching for 30 host larvae in a patch of yeast placed on an agar base in a Petri dish. The host larvae were in their late-second instar, and the parasitoid had had experience of oviposition during the previous 24 hours. When a larva was seen to be parasitized, it was transferred to a fresh Petri dish, where it was allowed to develop at 20°C for a fixed period of time before harvesting for RNA extraction. Ten parasitized larvae were collected per female, and larvae attacked within a short time frame (within 1-30 minutes, depending on the time point that was being collected) were reared together in the same dish. Larvae attacked by the parasitoid but rejected (defined by the ovipositor inserted for <10 s [67]) were not used in the study. We collected larvae at nine different times after parasitism: 10-15 minutes, 1 hour, 2 hours, 3 hours, 6 hours, 12 hours, 24 hours, 48 hours and 72 hours. To control for handling trauma, any variation in developmental stage across replicates, and the effect of the circadian rhythm on gene expression, a second pair of Petri dishes was set up in parallel, and the larvae treated identically except that they were not exposed to the parasitoid. At harvest, larvae were carefully teased from the medium with a spatula, snap-frozen in liquid nitrogen, and then stored at -80°C until RNA extraction. Sample collection for the study took 7 weeks.

### RNA isolation and array hybridizations

Microarray hybridizations (Affymetrix *Drosophila *Genome 1 Array) were performed for five biologic replicates per time point for both parasitized and control larvae (90 chips used in total). Because of circadian patterns in gene expression and possible changes in experimental conditions over the 7 weeks, the RNA used for each hybridization was pooled from flies harvested at different times of day and from over the complete collection period. In preparing samples, the paired sets of control and parasitized larvae continued to be handled together. To avoid large differences in RNA concentrations in the sample pools, the number of fly larvae used per biologic replicate depended on their age (less than 12 hours post-parasitism, 120 larvae; 12 hours, 100 larvae; 24 hours, 50 larvae; 48 and 72 hours, 30 larvae).

Preparation of material for the microarray analysis largely followed the Affymetrix manual. Briefly, samples were homogenized in 1 ml Trizol in FastPrep tubes (Lysing Matrix D; Q-Biogene, Morgan Irvine, CA, USA) using a bead mill (Hybaid RiboLyser; Hybaid, Teddington, UK). Total RNA was isolated using Trizol reagent (Invitrogen, Carlsbad, CA, USA) and the RNeasy (Qiagen, Hilden Germany) kit, following the manufacturers' instructions. For the RNA precipitation step in the Trizol protocol, 700 μl 70% diethyl pyrocarbonate-treated H_2_O-ethanol was used, and this volume was then applied directly onto RNeasy mini columns. The RNeasy protocol was then followed from the RW1 wash step onward. For each sample, double-stranded cDNA was synthesized from 20 μg total RNA using a commercially available kit (Roche Biochemicals, Basel, Switzerland). Biotin-labeled cRNA was then transcribed using T7 RNA polymerase and the BioArray Transcript labelling kit (Enzo, Farmingdale, NY, USA), followed by probe hydrolysis in 5 μl buffer (200 mmol/l Tris-acetate, pH 8.1, 500 mmol/l KOAc, 150 mmol/l MgOAc). The quality of total RNA and cRNA, and the fragmentation were checked using an Agilent Bio-analyzer (Agilent Technology, Palo Alto, CA, USA). The fragmented cRNA samples were stained, hybridized, and scanned by the Affymetrix microarray service at MRC Geneservices (Hinxton, UK).

### Microarray analysis

Initial manipulation of the raw intensity data from the hybridizations was performed using the 'affy package' [68] of the Bioconductor Project [69,70]. An estimate of the logarithmically transformed expression level of each gene based on the intensity of the different probe sets was obtained using the RMA method (robust multi-array analysis [71]) with standard settings (for example quantile normalization and calculation of expressions levels using median polish).

We analyzed gene expression levels using the *R *statistical package [72]. For each of the 14,010 probe sets on the Affymetrix chip, we had 90 data points representing five replicate measurements of expression levels in (paired sets of) control and parasitized larvae at each of nine time points (after parasitism). We knew that expression levels would vary with time because host larvae molted from the second to the third instar and initiated metamorphosis during the 72 hours of study. To detect effects of parasitism, we therefore carried out a mixed-model analysis of variance for each gene by first fitting a nine-level fixed 'time factor' and a random 'pair factor', and then testing for significance by adding the nested treatment × time interaction. This nested interaction term allowed us to test whether variation in expression values could be attributed to treatment (that is, attack by a parasitoid) across all time points or during a subset of time points. Analysis of variance makes specific assumptions about the distribution of the statistical error terms, and we confirmed that this method was appropriate by checking the form of residual plots of all genes with a significant treatment interaction effect and, for a subset of 25 genes, by repeating the analysis using an empirical *F *distribution constructed using random permutation [73]. Because we were conducting a large number of statistical tests, we could not rely on simple *P *values as a measure of statistical significance. Instead we used the positive false discovery rate method of Storey [74] and Storey and Tibshirani [75] and identified a set of significant genes while accepting a rate of false positives of 1%.

Within the set of genes that exhibited a significant response to parasitoid attack, we identified subsets with common patterns of expression using a clustering algorithm based on Pearson correlation coefficients and implemented in GeneSpring (version 6.2; Silicon Genetics, now acquired by Agilent Technology). Greater weights were assigned to later time points and to parasitized samples, which is where the largest variation in expression patterns was observed. The threshold for defining clusters was initially chosen by eye, although we checked that the clusters were reasonably robust by varying the parameters of the clustering algorithm.

The complete set of raw and normalized microarray data from this study is accessible through the public repository ArrayExpress at the European Bioinformatics Institute (accession number E-MAXD-6) [76]. Data produced during this project is also catalogued in EnvBase (accession number egcat:000031) [77]. The normalized data of the probe sets that exhibited a significant response to parasitoid attack are provided in Additional data file 5.

### Bioinformatics

We used bioinformatic tools to annotate the probe sets with significantly different expression profiles in parasitized and control larvae, and to look for patterns indicating functional relations and co-regulation in the major clusters of co-expressed genes.

The probes on Affymetrix microarray chips are arranged in probe sets, and we first associated these with the genes listed in the *Drosophila *genome project (FlyBase [78]), which we accessed through the Ensembl project web portal (version 16.3 [79]). Every individual probe sequence (usually 14 per probe set) was aligned against all available transcript sequences and matches (allowing for one error) recorded. Cases in which four probes from a probe set matched more than one gene, and those in which fewer than 10 probes matched the same gene were excluded, which meant that some probe sets remained unannotated. For 22% of the probe sets whose expression was influenced by parasitism, the probe set matched more than one transcript sequence, and in these cases annotation information from all peptides is provided. In our analyses, we used information on molecular function and biologic process from GeneOntology (September 2004 annotation [80]), and protein families, domains and functional sites from InterPro (version 7.1 [81]).

To determine whether sets of co-expressed genes identified using the clustering algorithm shared structural or functional traits, we asked whether genes in a cluster shared a particular annotation more often than expected by chance (using the program EASE [82]). EASE calculates the exact probability of randomly sampling a given number of genes with any particular annotation in relation to the total number of genes with this functional or structural annotation on the gene chip. Thus, it searches for annotations or 'biologic themes' that are statistically enriched in a group of genes as compared with the whole genome. Using EASE annotations for each probe set, the one-tailed Fisher exact probabilities and Bonferroni corrections were used to determine which particular annotation categories were over-represented.

We explored whether genes in the same cluster shared upstream motifs, including TFBMs, which might indicate coordinated expression. To do this we used the program MotifRegressor [83,84] to define a set of candidate motifs in the -1,000 to +50 base pair region of the differentially expressed genes after parasitoid attack, and then used the program Clover [85,86] to test for significant over-representation of these motifs in co-expressed genes. To define the set of candidate motifs information from individual time points were analyzed separately, and from each we retained the 20 top motifs that the program MotifRegressor identified using a regression strategy based on differential gene expression and the number and strength of match of the motif. Basically, the program searches for any sequence that is significantly associated with upregulated (or downregulated) genes. To test for over-representation of these motifs in gene clusters, the program Clover generates a score for each motif/cluster combination based on data on presence and strength of association. Initial screening identified more than 100 candidate motifs at the different time points. However, this list was reduced to six in a two-step approach: first, by merging degenerate motifs that aligned at more than half of the DNA bases per sequence, using IUPAC Ambiguous DNA Characters [38] to designate more than one DNA base at a given position within a sequence; and second, by requiring that motifs should be over-represented at multiple time points. Matches to known binding sites were identified using the TFBM databases Transfac 8.1 [87] and Jaspar [88,89], and from the list of immunity-related TFBMs presented by Senger and coworkers [36]. The significance of the associations was tested using scores generated from the -1,000 to +50 base pair regions of 1,000 randomly selected genes present on the Affymetrix *Drosophila *Genome 1 Array. Only clusters with more than five genes were included in the analysis.

## Additional data files

The following additional data are included with the online version of this article: A table annotating the probe sets with significantly different expression profiles in parasitized and control larvae (Additional data file 1); a figure showing the expression profile and upstream motifs of all genes per cluster (Additional data file 2); a table providing a full list of putative regulatory motifs that were significantly over-represented in our clusters of genes (Additional data file 3); a diagrammatic representation of the degenerate motifs of the putative TFBMs (Additional data file 4); and a table providing normalized data for the probe sets with significantly differential expression profiles in parasitized and control larvae (Additional data file 5).

## Supplementary Material

Additional data file 1 Annotation of probe sets with significantly different expression profiles in parasitized and control larvae. The annotation includes a matching score describing the fraction of the 14 probes that matched perfectly to the annotated transcript sequence, the gene name(s) assigned to probe sets, and functional and structural annotations for each gene. Whenever more than one gene was assigned to a probe set, an asterisk indicates which annotation was used in the EASE analysis. Click here for file

Additional data file 2 Expression profile and upstream motifs of all genes per cluster. The log_2 _expression values at the time points (hours) after parasitoid attack are shown for all replicates (blue circles for the control larvae; red triangles for the parasitized larvae) and the lines denote the average expression at each time point. The strength of match for the putative regulatory motifs in the upstream sequences is indicated by the height of the bars. Click here for file

Additional data file 3 A full list of putative regulatory motifs that were significantly over-represented in our clusters of genes. Motifs were identified by MotifRegressor and over-representation was calculated in Clover. The Representative Motifs denote the degenerate motifs, using IUPAC Ambiguous DNA Characters. The Raw Score measures the strength of match and the frequency of occurrence. Significance (denoted in the last two columns) is based on the comparison with the upstream sequences of 1,000 randomly chosen genes represented on the Affymetrix *Drosophila* 1 Genome Array, respectively - all genes on *Drosophila *chromosome 2. Click here for file

Additional data file 4 A diagrammatic representation of the degenerate motifs of the putative TFBMs. The size of the letters represents the likelihood of its occurrence at each position in the sequence. Click here for file

Additional data file 5 Normalized data of the probe sets with significantly differential expression profiles in parasitized and control larvae. The expression values (log_2 _transformed) for the five biologic replicates at each of nine time points after parasitism are provided for the paired sets of parasitized and control larvae. Click here for file

## Figures and Tables

**Table 1:** Putative regulatory motifs that were over-represented in the eight major clusters of differentially expressed genes

Motif	Time point (hours)	Cluster, raw score and significance^†^		
*Relish*/NF-κB-like	1, 3, 48	Cluster 1	8.54	*P *< 0.001
		Cluster 2	8.01	*P *= 0.002
		Cluster 11	5.09	*P *< 0.006
		Cluster 12	17.3	*P *< 0.001
		Cluster 14	12.4	*P *= 0.001
*serpent*/GATA-like	1, 2, 3, 6, 72	Cluster 1	7.13	*P *< 0.001
		Cluster 2	21.2	*P *< 0.001
		Cluster 9	17.5	*P *< 0.001
		Cluster 10	8.43	*P *= 0.009
		Cluster 12	10.5	*P *= 0.001
STAT	-	Cluster 2	4.88	*P *< 0.001
		Cluster 12	4.83	*P *< 0.001
TATA-like	72	Cluster 1	5.57	*P *= 0.001
		Cluster 9	13.9	*P *< 0.001
		Cluster 10	6.21	*P *= 0.002
CCARCAGRCCSA	1, 2, 3, 6	Cluster 2	56.1	*P *< 0.001
		Cluster 12	27.8	*P *< 0.001
		Cluster 14	14.3	*P *= 0.001
CAWTSKATTC	2, 3	Cluster 2	17.5	*P *< 0.001
		Cluster 14	8.39	*P *= 0.008
AMTCAGT	2, 3, 6, 12, 72	Cluster 2	16.6	*P *< 0.001
		Cluster 12	10.9	*P *< 0.001
		Cluster 14	8.99	*P *= 0.001

Putative motifs were identified as described in the text. The table shows the motifs identified, the time points at which they were significantly associated, and the clusters in which they appeared. For each cluster we give the raw score (a measure of the average occupancy in a set of sequences) and the associated significance value. ^†^Only for clusters with more than five genes.

**Table 2:** Survey of candidate genes previously implicated in *Drosophila *defense and immunity

Functional classification of proteins or genes	Differentially expressed candidate gene	Cluster number
**Hemocyte proliferation and differentiation^a^**		
JAK/STAT pathway		
Ligands	-	
Receptors	*dome (CG14226), CG14225*	2
JAK	*hop (CG1594)*	2
STAT	-	
Possible effector molecules	*TepI (CG18096), TepII (CG7052), TepIV (CG10363)*	12
	*TotB (CG5609)*	8
Toll pathway (in lymph glands)		
Ligands	-	
Regulators of pathway	*nec (CG1857)*	2
Receptors	*Tl (CG5490)*	3
Intracellular signaling elements	-	
NF-κB transcription factor	*Relish (CG11992)*	4
Ras/Raf/MAPK pathway	-	
Notch pathway	-	
VEGF receptor pathway	-	
GATA factor homologs (e.g. *srp*)	-	
RUNX/AML1-like proteins (*lz*)	-	
**Cellular defense, in particular encapsulation^b^**		
Recognition/surface binding factors		
Extracellular matrix (ECM) proteins (e.g. laminin, collagen IV, fibronectin)	*dome (CG14226)*	2
	*prc (CG5700)*	14
	*Hml (CG7002)*	10
	*CG6788/CG32496*	11
Integrins	*αPS4 (CG16827)*	11
Immunoglobulin superfamily members	*Pxn (CG12002)*	6
	*CG8100*	10
	*CG14225*	13
Scavenger receptors (CD36-like)	*CG12789*	4
	*CG2736*	10
	*Tequila (CG4821)*	12
Possible pattern recognition receptors	*lectin-24A (CG3410)*	12
G-protein type receptors	*mthl2 (CG17795)*	11
Surface helper molecules		
Vinculin, talin, paxillins	-	
Surface-associated signaling molecules		
Integrin-linked focal adhesion kinases (FAKs)	-	
Integral membrane proteins	*rost (CG9552)*	4
	*Tsp42Ek (CG12841)*	9
Intracellular signaling pathway factors		
Phosphotidylinositol 3-kinase (PI3K)	-	
GTP-binding proteins (Ras/Rho family members)	-	
Protein kinase C (PKCs) or PKC regulators	*CG5958 (PKC transporter)*	10
Protein tyrosine phosphatase (PTPs)	*dome (CG14226)*	2
Serine/threonine kinases	-	
Scaffolding proteins (RACK)	-	
Cytoskeletal proteins (actins, tubulins, for example)	*αTub85E (CG9476), αTub84D (CG2512), αTub84E (CG1913), βTub60D (CG3401)*	11
Eicosinoid pathway elements	-	
Effector molecules		
NO pathway factors	-	
PPO pathway factors	*Dox-A3 (CG2952), CG11313,*	*11*
	*G8193, Bc (CG5779),*	*9*
	*Fmo-2 (CG3174)*	*15*
Porferins or related molecules	-	
Tumor necrosis factor (TNFs)	*CG13559*	2
**Humoral defense^b^**		
Humoral pattern-recognition receptors	*PGRP-SB1 (CG9681)*	1
	*lectin-24A (CG3410)*	12
	*Hml (CG7002)*	10
Serine proteases	*CG3066*	2
	*CG30414, CG30086, CG30090, Tequila (CG4821), CG16705, CG31780 / BG:DS07108.1 (CG18477), CG6639, CG3117, CG31827/BG:DS07108.5 (CG18478), CG18563, CG4793, CG4259*	12
	*CG11313*	11
	*CG16713*	4
Serpins and other protease inhibitors	*nec*	2
	*CG6687, CG16712, CG16705, TepI (CG18096), TepII (CG7052), TepIV (CG10363)*	12
	*BcDNA:SD04019 (CG17278)*	14
	*CG16704*	1
Known ligand-like molecules (e.g. *spz*)	-	
Surface receptors		
Toll and associated family members	*TI (CG5490)*	3
Toll or imd pathway (in fatbody)		
Intracellular signalling elements (e.g., tube, Pelle, DTRAF, DECSIT)	-	
NF-κB transcription factor	*Rel (CG11992)*	4
Effector molecules or antimicrobial peptides	*AttA (CG10146), AttB (CG18372)*	1
	*Mtk (CG8175), IM1 (CG18108), IM2 (CG18106), CG13422, CG15066*	2
	*IM4 (CG15231), CG18279, CG16844*	14
Related apoptotic regulators		
Dredd	-	
Ubiquitins	-	
PPO and associated pathway molecules	*Dox-A3 (CG2952)*	11
Melanin and free-radical intermediates	*Fmo-2 (CG3174)*	15

The table lists the different functional classes of genes and protein surveyed, any genes in these classes that were differentially expressed, and the cluster the gene was assigned to. Note that some genes with multiple annotations can appear in more than one category.

^a^Based on [17,66,90,91]; ^b^based on [11,92] (MR Strand, personal communication).
