# Supplementary material for: Genome-wide gene expression in response to parasitoid attack in Drosophila
Source: Genome Biol. 2005 Oct 31;6(11):R94. doi: 10.1186/gb-2005-6-11-r94 (PMC1297650; doi:10.1186/gb-2005-6-11-r94)
Supplement: Additional data file 1 — Annotation of probe sets with significantly different expression profiles in parasitized and control larvae. The annotation includes a matching score describing the fraction of the 14 probes that matched perfectly to the annotated transcript sequence, the gene name(s) assigned to probe sets, and functional and structural annotations for each gene. Whenever more than one gene was assigned to a probe set, an asterisk indicates which annotation was used in the EASE analysis. [file gb-2005-6-11-r94-S1.pdf]

Affymetrix ID	Gene Product	Matching Score	Gene	Gene Name	cluster number	GeneOntology Biological Process	GeneOntology Molecular Function	InterPro domain
141374_at	CG10146-PA	0.79	CG10146	AttA	1	6961 antibacterial humoral response (sensu Protostomia)<br>42742 defense response to bacteria<br>50829 defense response to Gram-negative bacteria		5520 Attacin, N-terminal region<br>5521 Attacin, C-terminal region
147220_s_at	CG18372-PA	1	CG18372	AttB*	1	6952 defense response<br>6961 antibacterial humoral response (sensu Protostomia)<br>42742 defense response to bacteria		5520 Attacin, N-terminal region<br>5521 Attacin, C-terminal region
	CG10146-PA	1	CG10146	AttA		6961 antibacterial humoral response (sensu Protostomia)<br>42742 defense response to bacteria<br>50829 defense response to Gram-negative bacteria		
152827_at	CG14704-PC<br>CG14704-PA<br>CG14704-PB	1<br>1<br>1	CG14704	PGRP-LB	1	6952 defense response<br>6955 immune response<br>16045 detection of bacteria	16019 peptidoglycan receptor activity	2502 N-acetylmuramoyl-L-alanine amidase, family 2<br>6619 Animal peptidoglycan recognition protein PGRP
145728_at	CG16704-PA	1	CG16704	CG16704	1		4867 serine-type endopeptidase inhibitor activity	1588 Casein, alpha/beta<br>2223 Pancreatic trypsin inhibitor (Kunitz)
148267_at	CG32405-PA<br>CG32404-PA	0.43<br>0.57	CG32405<br>CG32404	CG32405*<br>CG32404	1		5214 structural constituent of cuticle (sensu Insecta)<br>5214 structural constituent of cuticle (sensu Insecta)	618 Insect cuticle protein<br>618 Insect cuticle protein
150533_at	CG13641-PA	1	CG13641	CG13641	1			
144891_at	CG2444-PA	1	CG2444	CG2444	2			
147431_at	CG18108-PA	1	CG18108	IM1	2	6952 defense response		
147430_at	CG15066-PA	0.93	CG15066	CG15066	2	6952 defense response<br>8063 Toll signaling pathway<br>19731 antibacterial humoral response	5554 molecular_function unknown	
147546_at	CG13422-PA	1	CG13422	CG13422	2	5976 polysaccharide metabolism<br>6952 defense response<br>50829 defense response to Gram-negative bacteria	15926 glucosidase activity	
152100_at	CG31705-PA	1	CG31705	BcDNA:GH07269	2			
141685_at	CG3066-PA<br>CG3066-PC<br>CG3066-PB	1<br>1<br>1	CG3066	CG3066	2	6508 proteolysis and peptidolysis<br>6952 defense response	4185 serine carboxypeptidase activity<br>4295 trypsin activity<br>8439 monophenol monooxygenase activator activity	1254 Peptidase S1, chymotrypsin<br>1314 Peptidase S1A, chymotrypsin<br>6604 Disulphide knot CLIP<br>9003 Peptidase, trypsin-like serine and cysteine proteases
146726_at	CG1857-PA	1	CG1857	nec	2	6508 proteolysis and peptidolysis<br>6952 defense response<br>6966 antifungal humoral response (sensu Protostomia)<br>8063 Toll signaling pathway	4867 serine-type endopeptidase inhibitor activity	215 Serpin
143770_at	CG8175-PA	1	CG8175	Mtk	2	6952 defense response<br>6961 antibacterial humoral response (sensu Protostomia)<br>6966 antifungal humoral response (sensu Protostomia)<br>50830 defense response to Gram-positive bacteria<br>50832 defense response to fungi		
147432_at	CG18107-PA	1	CG18107	CG18107	2			
151210_at	CG16836-PA	1	CG16836	CG16836	2			
144068_at	CG18106-PA	1	CG18106	IM2	2	6952 defense response		
147433_at	CG15067-PA	1	CG15067	CG15067	2			9056 Cytochrome c

Affymetrix ID	Gene Product	Matching Score	Gene	Gene Name	cluster number	GeneOntology Biological Process	GeneOntology Molecular Function	InterPro domain
153404_at	CG1594-PA	1	CG1594	hop	2	910 cytokinesis;<br>6952 defense response;<br>6955 immune response;<br>6959 humoral immune response<br>6968 cellular immune response<br>7259 JAK-STAT cascade<br>7260 tyrosine phosphorylation of STAT protein<br>7262 STAT protein nuclear translocation<br>7298 border cell migration (sensu Insecta)<br>7350 blastoderm segmentation<br>7365 periodic partitioning<br>7398 ectoderm development<br>7399 neurogenesis<br>7424 tracheal system development (sensu Insecta)<br>7442 hindgut morphogenesis<br>7456 eye morphogenesis (sensu Drosophila)<br>7467 photoreceptor differentiation (sensu Drosophila)<br>7476 wing morphogenesis<br>7480 leg morphogenesis (sensu Holometabola)<br>7498 mesoderm development<br>7530 sex determination<br>7538 primary sex determination<br>8283 cell proliferation<br>9993 oogenesis (sensu Insecta)<br>16318 ommatidial rotation<br>19827 stem cell maintenance<br>30097 hemopoiesis<br>30707 ovarian follicle cell development (sensu Insecta)<br>35010 encapsulation of foreign target<br>35171 lamellocyte differentiation<br>35172 hemocyte proliferation (sensu Arthropoda)<br>42067 establishment of ommatidial polarity (sensu Drosophila)<br>42078 germ-line stem cell renewal<br>42386 hemocyte differentiation (sensu Arthropoda)<br>45317 equator specification<br>48103 somatic stem cell renewal	4713 protein-tyrosine kinase activity<br>4715 non-membrane spanning protein tyrosine kinase activity<br>4718 Janus kinase activity	299 Band 4.1<br>719 Protein kinase<br>980 SH2 motif<br>1245 Tyrosine protein kinase<br>6025 Peptidase M, neutral zinc metallopeptidases, zinc-binding site<br>8266 Tyrosine protein kinase, active site
143053_at	CG11648-PD<br>CG11648-PB<br>CG11648-PA<br>CG11648-PC	1<br>1<br>1<br>1	CG11648	Abd-B	2	35215 genital disc development<br>45705 negative regulation of salivary gland determination<br>45843 negative regulation of myogenesis<br>48066 pigmentation	3704 specific RNA polymerase II transcription factor activity	1356 Homeobox<br>9057 Homeodomain-like
149053_at	CG9451-PA	1	CG9451	CG9451	2	6796 phosphate metabolism	3993 acid phosphatase activity	560 Histidine acid phosphatase
147785_at	CG13559-PA	1	CG13559	CG13559	2			6629 LPS-induced tumor necrosis factor alpha factor
149779_at	CG18549-PA	1	CG18549	CG18549	2			10291 Eukaryotic protein of unknown function DUF895
149964_at	CG14872-PB<br>CG14872-PA	1<br>0.64	CG14872	CG14872	2			566 Lipocalin-related protein and Bos/Can/Equ allergen
142932_at	CG14226-PA	1	CG14226	dome	2	2009 morphogenesis of an epithelium<br>6470 protein amino acid dephosphorylation<br>6955 immune response<br>7259 JAK-STAT cascade<br>7298 border cell migration (sensu Insecta)<br>7350 blastoderm segmentation<br>7424 tracheal system development (sensu Insecta)<br>7425 tracheal cell fate determination (sensu Insecta)<br>7442 hindgut morphogenesis	4725 protein tyrosine phosphatase activity<br>4888 transmembrane receptor activity<br>4907 interleukin receptor activity<br>19955 cytokine binding	194 H+-transporting two-sector ATPase, alpha/beta subunit, central region<br>3961 Fibronectin, type III<br>8957 Fibronectin, type III-like fold

Affymetrix ID	Gene Product	Matching Score	Gene	Gene Name	cluster number	GeneOntology Biological Process	GeneOntology Molecular Function	InterPro domain
153038_at	CG3135-PA	1	CG3135	CG3135	2			742 EGF-like domain, subtype 2<br>3306 WIF domain<br>6209 EGF-like domain<br>6210 Type I EGF<br>9030 Growth factor, receptor
151935_at	CG5490-PA<br>CG5490-PB	1<br>1	CG5490	Tl	3	910 cytokinesis<br>6952 defense response<br>6955 immune response<br>6960 antimicrobial humoral response (sensu Protostomia)<br>6963 antibacterial polypeptide induction<br>6966 antifungal humoral response (sensu Protostomia)<br>6967 antifungal polypeptide induction<br>7155 cell adhesion<br>7352 zygotic determination of dorsal/ventral axis<br>8063 Toll signaling pathway<br>9617 response to bacteria<br>9620 response to fungi<br>9880 embryonic pattern specification<br>9950 dorsal/ventral axis specification<br>19221 cytokine and chemokine mediated signaling pathway<br>30097 hemopoiesis<br>35007 regulation of melanization defense response<br>35172 hemocyte proliferation (sensu Arthropoda)<br>42742 defense response to bacteria<br>45087 innate immune response<br>50830 defense response to Gram-positive bacteria<br>[illegible]	4888 transmembrane receptor activity<br>19955 cytokine binding	157 TIR<br>372 Cysteine-rich flanking region, N-terminal<br>483 Cysteine-rich flanking region, C-terminal<br>1611 Leucine-rich repeat<br>3591 Leucine-rich repeat, typical subtype<br>4075 Interleukin-1 receptor, type I/Toll precursor
147450_at	CG5493-PA	1	CG5493	CG5493	3	9063 amino acid catabolism	17172 cysteine dioxygenase activity	10300 Cysteine dioxygenase type I
151822_at	CG11992-PA	1	CG11992	Rel	4	6357 regulation of transcription from Pol II promoter<br>6955 immune response<br>6963 antibacterial polypeptide induction<br>6967 antifungal polypeptide induction<br>6968 cellular defense response<br>7165 signal transduction<br>7249 I-kappaB kinase/NF-kappaB cascade<br>8063 Toll signaling pathway<br>9617 response to bacteria<br>45449 regulation of transcription<br>50829 defense response to Gram-negative bacteria	3700 transcription factor activity<br>3704 specific RNA polymerase II transcription factor activity<br>5515 protein binding<br>30528 transcription regulator activity	451 NF-kappa-B/Rel/dorsal<br>2110 Ankyrin<br>2909 Cell surface receptor IPT/TIG<br>8967 p53-like transcription factor
146022_at	CG14275-PA<br>CG14275-PB	1<br>1	CG14275	CG14275	4			
152891_at	CG12789-PA<br>CG12789-PB	1<br>1	CG12789	CG12789	4	6629 lipid metabolism<br>6869 lipid transport<br>6915 apoptosis<br>6952 defense response<br>7010 cytoskeleton organization and biogenesis<br>7165 signal transduction<br>35071 salivary gland cell death<br>42116 macrophage activation<br>48102 autophagic cell death	5044 scavenger receptor activity	2159 CD36 antigen<br>5428 Adhesion molecule CD36
145730_at	CG16713-PA	1	CG16713	CG16713	4		4867 serine-type endopeptidase inhibitor activity	2223 Pancreatic trypsin inhibitor (Kunitz)
154129_at	CG9552-PA	1	CG9552	rost	4	7498 mesoderm development<br>7519 myogenesis<br>7520 myoblast fusion		
146165_at	CG6113-PA	1	CG6113	CG6113	4	6629 lipid metabolism	4806 triacylglycerol lipase activity	73 Alpha/beta hydrolase fold<br>379 Esterase/lipase/thioesterase<br>504 RNA-binding region RNP-1 (RNA recognition motif)<br>6693 Ab-hydrolase associated lipase region<br>8262 Lipase, active site

Affymetrix ID	Gene Product	Matching Score	Gene	Gene Name	cluster number	GeneOntology Biological Process	GeneOntology Molecular Function	InterPro domain
141450_at	CG1982-PA	1	CG1982	Sodh-1	5	5975 carbohydrate metabolism	3939 L-iditol 2-dehydrogenase activity	205 NAD-binding site<br>2085 Zinc-containing alcohol dehydrogenase superfamily<br>2328 Zinc-containing alcohol dehydrogenase
146461_at	CG10621-PA	0.93	CG10621	CG10621	5	8652 amino acid biosynthesis	16205 selenocysteine methyltransferase activity	3726 Homocysteine S-methyltransferase
152709_at	CG10078-PA<br>CG10078-PB	1<br>1	CG10078	Prat2	6	5996 monosaccharide metabolism<br>6144 purine base metabolism<br>6189 'de novo' IMP biosynthesis	4044 amidophosphoribosyltransferase activity	583 Glutamine amidotransferase, class-II<br>834 Peptidase M14, carboxypeptidase A<br>836 Phosphoribosyltransferase<br>2375 Purine/pyrimidine phosphoribosyl transferase<br>5854 Amidophosphoribosyl transferase
152747_at	CG12002-PA	1	CG12002	Pxn	6	6800 oxygen and reactive oxygen species metabolism<br>6952 defense response	4601 peroxidase activity	372 Cysteine-rich flanking region, N-terminal<br>1007 von Willebrand factor, type C<br>1611 Leucine-rich repeat<br>2007 Animal haem peroxidase<br>3591 Leucine-rich repeat, typical subtype<br>3598 Immunoglobulin C-2 type<br>7110 Immunoglobulin-like<br>10255 Haem peroxidase
141397_at	CG8502-PC<br>CG8502-PA	1<br>1	CG8502	CG8502	6		8010 structural constituent of larval cuticle (sensu Insecta)	618 Insect cuticle protein
151301_at	CG13315-PA	1	CG13315	CG13315	6			
154385_at	CG30388-PA	1	CG30388	Magi	6	6605 protein targeting<br>7242 intracellular signaling cascade<br>8105 asymmetric protein localization	4385 guanylate kinase activity<br>5102 receptor binding	1202 WW/Rsp5/WWP domain<br>1478 PDZ/DHR/GLGF domain
147879_at	CG4781-PA	1	CG4781	CG4781	7			1611 Leucine-rich repeat<br>3591 Leucine-rich repeat, typical subtype
150284_at	CG5609-PA	1	CG5609	TotB	8	16065 humoral defense mechanism (sensu Protostomia)		10825 Stress-inducible humoral factor Turandot
152063_at	CG4716-PB<br>CG4716-PA	0.71<br>1	CG4716	CG4716	8		4486 methylenetetrahydrofolate dehydrogenase activity	
152995_at	CG18316-PA	1	CG18316	CG18316	8			
155091_at	CG10596-PA<br>CG10596-PB<br>CG10596-PC	1<br>1<br>1	CG10596	Msr-110	8			
146714_at	CG12841-PA	1	CG12841	Tsp42Ek	9	7398 ectoderm development<br>7399 neurogenesis<br>19226 transmission of nerve impulse	5057 receptor signaling protein activity	301 CD9/CD37/CD63 antigen<br>8952 Tetraspanin
148725_at	CG8745-PA	1	CG8745	CG8745	9	6520 amino acid metabolism	4587 ornithine-oxo-acid transaminase activity	5814 Aminotransferase class-III<br>10164 Ornithine aminotransferase
141430_at	CG11491-PA<br>CG11491-PF	0.071429	CG11491	br	9	6355 regulation of transcription, DNA-dependent<br>6357 regulation of transcription from Pol II promoter<br>6914 autophagy<br>7458 progression of morphogenetic furrow (sensu Drosophila)<br>7459 photoreceptor fate commitment (sensu Drosophila)<br>7530 sex determination<br>7552 metamorphosis<br>7562 eclosion<br>8219 cell death<br>9613 response to pest, pathogen or parasite<br>19226 transmission of nerve impulse<br>35070 salivary gland histolysis<br>35071 salivary gland cell death<br>35072 ecdysone-mediated induction of salivary gland cell death<br>35075 response to ecdysone	3677 DNA binding<br>3700 transcription factor activity<br>3704 specific RNA polymerase II transcription factor activity	210 BTB/POZ domain<br>345 Cytochrome c heme-binding site<br>1559 Aryldialkylphosphatase<br>7087 Zn-finger, C2H2 type
142955_at	CG5770-PA	1	CG5770	CG5770	9			7931 Protein of unknown function DUF725
147487_r_at	CG18606-PA	1	CG18606	CG18606	9			
147772_at	CG9891-PA	1	CG9891	yellow-d2	9			3534 Major royal jelly protein

Affymetrix ID	Gene Product	Matching Score	Gene	Gene Name	cluster number	GeneOntology Biological Process	GeneOntology Molecular Function	InterPro domain
143348_at	CG7596-PA	1	CG7596	Sgs5	9	7594 puparial adhesion	5198 structural molecule activity	
143493_at	CG7604-PA	0.86	CG7604	Eig71Ee	9	7594 puparial adhesion	5198 structural molecule activity	8997 Ricin B-related lectin
148817_at	no matches				9			
143347_at	CG12181-PB	1	CG12181	Sgs4	9	7594 puparial adhesion	5198 structural molecule activity	
143349_at	CG18087-PA	1	CG18087	Sgs7	9	7594 puparial adhesion	5198 structural molecule activity	
150084_at	CG7587-PA	1	CG7587	CG7587	9			
143158_at	CG17285-PA<br>CG17285-PB	0.96<br>0.96	CG17285	Fbp1	9	15032 fat body storage protein uptake	8565 protein transporter activity	5203 Hemocyanin, C-terminal<br>5204 Hemocyanin, N-terminal
143350_at	CG6132-PA	1	CG6132	Sgs8	9	7594 puparial adhesion	5198 structural molecule activity	
144980_at	CG12715-PA	1	CG12715	CG12715	9			8166 Protein of unknown function DUF23
143346_at	CG11720-PA	1	CG11720	Sgs3	9	7594 puparial adhesion	5198 structural molecule activity	
145696_at	CG15404-PA	1	CG15404	CG15404	9			
151052_at	no matches				9			
142220_at	CG8193-PA	1	CG8193	CG8193	9	6952 defense response	4503 monophenol monooxygenase activity	2227 Tyrosinase<br>5203 Hemocyanin, C-terminal<br>5204 Hemocyanin, N-terminal<br>8922 Di-copper centre-containing<br>896 Arthropod hemocyanin/insect LSP
151955_at	CG5779-PA	1	CG5779	Bc	9	6952 defense response<br>9611 response to wounding<br>9613 response to pest, pathogen or parasite<br>35006 melanization defense response	4503 monophenol monooxygenase activity	896 Arthropod hemocyanin/insect LSP<br>2227 Tyrosinase<br>5203 Hemocyanin, C-terminal<br>5204 Hemocyanin, N-terminal<br>8922 Di-copper centre-containing
144738_at	CG15369-PA	1	CG15369	CG15369	10		4869 cysteine protease inhibitor activity	10 Cysteine protease inhibitor<br>3244 Sarcocystatin
151802_at	CG32954-PC<br>CG32954-PG<br>CG32954-PB<br>CG32954-PE<br>CG32954-PD<br>CG32954-PH<br>CG32954-PF<br>CG32954-PA	0.86<br>0.86<br>0.86<br>0.86<br>0.86<br>0.86<br>0.86<br>0.86	CG32954	CG32954	10			205 NAD-binding site<br>2198 Short-chain dehydrogenase/reductase SDR<br>2424 Insect alcohol dehydrogenase family<br>2425 Insect alcohol dehydrogenase<br>2427 Alcohol dehydrogenase-related protein
152569_at	CG30019-PC<br>CG30019-PA<br>CG30019-PB	1<br>1<br>1	CG30019	CG30019	10			262 FMN-dependent alpha-hydroxy acid dehydrogenase<br>3009 FMN/related compound-binding core<br>8259 FMN-dependent alpha-hydroxy acid dehydrogenase, active site
148743_at	CG8100-PA	1	CG8100	CG8100	10			5203 Hemocyanin, C-terminal<br>5204 Hemocyanin, N-terminal<br>8922 Di-copper centre-containing
150576_at	CG10514-PA	1	CG10514	CG10514	10			4119 Protein of unknown function DUF227
146585_at	CG8665-PA	1	CG8665	CG8665	10	9063 amino acid catabolism	16155 formyltetrahydrofolate dehydrogenase activity	1555 Phosphoribosylglycinamide formyltransferase, active site<br>2086 Aldehyde dehydrogenase<br>2376 Formyl transferase, N-terminal<br>5793 Formyl transferase, C-terminal<br>6163 Phosphopantetheine-binding domain<br>9081 Acyl carrier protein-like
143009_s_at	CG10962-PB	1	CG10962	CG10962	10		16616 oxidoreductase activity, acting on the CH-OH group of donors, NAD or NADP as acceptor	2198 Short-chain dehydrogenase/reductase SDR<br>2347 Glucose/ribitol dehydrogenase
146569_at	CG9259-PA	1	CG9259	CG9259	10			345 Cytochrome c heme-binding site<br>4119 Protein of unknown function DUF227
147664_at	CG3292-PA	1	CG3292	CG3292	10	1501 skeletal development<br>7498 mesoderm development	4035 alkaline phosphatase activity<br>19204 nucleotide phosphatase activity	1952 Alkaline phosphatase
152648_at	CG2736-PA	1	CG2736	CG2736	10	6952 defense response	5044 scavenger receptor activity	2159 CD36 antigen
147740_at	CG9877-PA	1	CG9877	CG9877	10			
151932_at	CG13279-PA	1	CG13279	Cyt-b5-r	10		5489 electron transporter activity	1199 Cytochrome b5<br>5804 Fatty acid desaturase
149914_at	CG7390-PB	1	CG7390	smp-30	10	7165 signal transduction	5509 calcium ion binding	2372 Bacterial quinoprotein

Affymetrix ID	Gene Product	Matching Score	Gene	Gene Name	cluster number	GeneOntology Biological Process	GeneOntology Molecular Function	InterPro domain
	CG7390-PA	1				7242 intracellular signaling cascade<br>7275 development<br>9948 anterior/posterior axis specification<br>19722 calcium-mediated signaling		5511 Senescence marker protein-30 (SMP-30)
145956_at	CG5958-PA	1	CG5958	CG5958	10	6731 coenzyme and prosthetic group metabolism<br>15875 vitamin or cofactor transport	5386 carrier activity<br>16918 retinal binding	1071 Cellular retinaldehyde binding/alpha-tocopherol transport<br>1251 Cellular retinaldehyde-binding)/triple function, C-terminal<br>8273 Cellular retinaldehyde-binding/triple function, N-terminal
141370_at	CG7758-PA	1	CG7758	ppl	10	6546 glycine catabolism	16787 hydrolase activity	2930 Glycine cleavage H-protein<br>3016 2-oxo acid dehydrogenase, lipoyl-binding site
152781_at	CG3724-PA	1	CG3724	Pgd	10	6098 pentose-phosphate shunt	4616 phosphogluconate dehydrogenase (decarboxylating) activity	6113 6-phosphogluconate dehydrogenase, decarboxylating<br>6114 6-phosphogluconate dehydrogenase, C-terminal<br>6115 6-phosphogluconate dehydrogenase, NAD binding domain<br>6183 6-phosphogluconate dehydrogenase<br>6184 6-phosphogluconate-binding site<br>8927 6-phosphogluconate dehydrogenase, C-terminal-like
149624_at	CG3999-PA	1	CG3999	CG3999	10	9063 amino acid catabolism	4375 glycine dehydrogenase (decarboxylating) activity	3437 Glycine cleavage system P-protein
152571_at	CG7966-PA	1	CG7966	CG7966	10	6952 defense response	8430 selenium binding	8826 56kDa selenium binding
141375_at	CG5656-PA	1	CG5656	CG5656	10	1501 skeletal development<br>7498 mesoderm development	4035 alkaline phosphatase activity<br>19204 nucleotide phosphatase activity	1952 Alkaline phosphatase
152708_at	CG7002-PA	1	CG7002	Hml	10	6952 defense response<br>7155 cell adhesion<br>7599 hemostasis	5198 structural molecule activity<br>5529 sugar binding	421 Coagulation factor 5/8 type C domain (FA58C)<br>436 Sushi domain/SCR domain/CCP module<br>1007 von Willebrand factor, type C<br>1846 von Willebrand factor, type D<br>2172 Low density lipoprotein-receptor, class A<br>2557 Chitin binding Peritrophin-A<br>2919 Trypsin inhibitor-like, cysteine-rich TIL region<br>6207 Cystine knot, C-terminal<br>6209 EGF-like domain<br>6210 Type I EGF 742 EGF-like domain, subtype 2
152265_at	CG11892-PA<br>CG11892-PB	1<br>1	CG11892	CG11892	10			4119 Protein of unknown function DUF227
143389_f_at	CG2512-PB<br>CG2512-PA	0.11<br>0.11	CG2512	alphaTub84D*	11	6886 intracellular protein transport<br>6928 cell motility<br>7017 microtubule-based process<br>7059 chromosome segregation<br>7067 mitosis	5200 structural constituent of cytoskeleton<br>5525 GTP binding<br>15631 tubulin binding	217 Tubulin<br>2452 Alpha tubulin<br>3008 Tubulin/FtsZ, GTPase<br>8280 Tubulin/FtsZ, C-terminal
	CG9476-PA	1	CG9476	alphaTub85E		6886 intracellular protein transport<br>6928 cell motility<br>7010 cytoskeleton organization and biogenesis<br>7059 chromosome segregation<br>7067 mitosis<br>8151 cell growth and/or maintenance	5200 structural constituent of cytoskeleton<br>5525 GTP binding<br>15631 tubulin binding	217 Tubulin<br>2452 Alpha tubulin<br>3008 Tubulin/FtsZ, GTPase<br>8280 Tubulin/FtsZ, C-terminal
	CG1913-PA	0.25	CG1913	alphaTub84B		6886 intracellular protein transport<br>6928 cell motility<br>7010 cytoskeleton organization and biogenesis<br>7059 chromosome segregation<br>7067 mitosis<br>8151 cell growth and/or maintenance	5200 structural constituent of cytoskeleton<br>5525 GTP binding<br>15631 tubulin binding	217 Tubulin<br>2452 Alpha tubulin<br>3008 Tubulin/FtsZ, GTPase<br>8280 Tubulin/FtsZ, C-terminal
145267_at	CG32496-PA	0.93	CG32496	CG32496*	11	42742 defense response to bacteria<br>6952 defense response		2181 Fibrinogen, beta/gamma chain, C-terminal globular
	CG6788-PA	1	CG6788	CG6788		7155 cell adhesion<br>42742 defense response to bacteria		2181 Fibrinogen, beta/gamma chain, C-terminal globular
142349_at	CG18550-PA	1	CG18550	yellow-f	11	2165 larval or pupal development (sensu Insecta)<br>42435 indole derivative biosynthesis<br>48066 pigmentation<br>6583 melanin biosynthesis from tyrosine<br>7398 ectoderm development	4167 dopachrome isomerase activity<br>5102 receptor binding<br>5198 structural molecule activity<br>16860 intramolecular oxidoreductase activity	3534 Major royal jelly protein

Affymetrix ID	Gene Product	Matching Score	Gene	Gene Name	cluster number	GeneOntology Biological Process	GeneOntology Molecular Function	InterPro domain
146311_at	CG9928-PA	1	CG9928	CG9928	11			
143392_r_at	CG3401-PA	1	CG3401	betaTub60D	11	6886 intracellular protein transport<br>7017 microtubule-based process<br>7059 chromosome segregation<br>7067 mitosis<br>7409 axonogenesis<br>7411 axon guidance<br>7507 heart development<br>9416 response to light<br>30537 larval behavior	5200 structural constituent of cytoskeleton<br>5525 GTP binding<br>15631 tubulin binding	217 Tubulin<br>2453 Beta tubulin<br>3008 Tubulin/FtsZ, GTPase<br>8280 Tubulin/FtsZ, C-terminal
148230_at	CG17795-PA<br>CG17795-PB	1<br>1	CG17795	mthl2	11	6950 response to stress<br>7186 G-protein coupled receptor protein signaling pathway<br>8340 determination of adult life span	4930 G-protein coupled receptor activity	276 Rhodopsin-like GPCR superfamily<br>832 G-protein coupled receptor family 2 (secretin-like)<br>10596 Methuselah, N-terminal
150908_at	CG11313-PA	1	CG11313	CG11313	11	6508 proteolysis and peptidolysis	4295 trypsin activity<br>8439 monophenol monooxygenase activator activity	1254 Peptidase S1, chymotrypsin<br>1314 Peptidase S1A, chymotrypsin<br>6604 Disulphide knot CLIP<br>9003 Peptidase, trypsin-like serine and cysteine proteases
147239_at	CG16827-PA	1	CG16827	alphaPS4	11	7155 cell adhesion<br>7157 heterophilic cell adhesion<br>7160 cell-matrix adhesion	4872 receptor activity<br>50839 cell adhesion molecule binding	413 Integrins alpha chain
141775_at	CG2952-PA	1	CG2952	Dox-A3	11	6952 defense response	4503 monophenol monooxygenase activity	896 Arthropod hemocyanin/insect LSP<br>2227 Tyrosinase<br>5203 Hemocyanin, C-terminal<br>5204 Hemocyanin, N-terminal<br>8922 Di-copper centre-containing
149425_at	CG14610-PA	1	CG14610	CG14610	11			
152216_at	CG1208-PA	1	CG1208	CG1208	11	5975 carbohydrate metabolism<br>8643 carbohydrate transport	5355 glucose transporter activity	3663 Sugar transporter<br>5828 General substrate transporter<br>5829 Sugar transporter superfamily<br>7114 Major facilitator superfamily
146214_at	CG16743-PA	1	CG16743	CG16743	11			
142387_at	CG30086-PA	1	CG30086	CG30086	12			1254 Peptidase S1, chymotrypsin<br>9003 Peptidase, trypsin-like serine and cysteine proteases
144222_at	CG4793-PC	1	CG4793	BG:DS07486.3	12		8047 enzyme activator activity	1254 Peptidase S1, chymotrypsin<br>1314 Peptidase S1A, chymotrypsin<br>1547 Glycoside hydrolase, family 5<br>9003 Peptidase, trypsin-like serine and cysteine proteases
141353_at	CG6687-PA	1	CG6687	CG6687	12	6508 proteolysis and peptidolysis	4867 serine-type endopeptidase inhibitor activity	215 Serpin<br>454 Eubacterial/plasma membrane H+-transporting two-sector ATPase, C subunit
147699_at	CG4250-PA	1	CG4250	CG4250	12			6209 EGF-like domain
146405_at	CG6639-PA	1	CG6639	CG6639	12			1254 Peptidase S1, chymotrypsin<br>1314 Peptidase S1A, chymotrypsin<br>9003 Peptidase, trypsin-like serine and cysteine proteases
145677_at	CG3117-PA	1	CG3117	CG3117	12			1254 Peptidase S1, chymotrypsin<br>9003 Peptidase, trypsin-like serine and cysteine proteases
147773_at	CG30414-PA	1	CG30414	CG30414	12	6508 proteolysis and peptidolysis<br>6521 regulation of amino acid metabolism<br>8652 amino acid biosynthesis	4295 trypsin activity	1254 Peptidase S1, chymotrypsin<br>1314 Peptidase S1A, chymotrypsin<br>9003 Peptidase, trypsin-like serine and cysteine proteases
145970_at	CG7052-PD<br>CG7052-PC<br>CG7052-PE<br>CG7052-PA<br>CG7052-PB	1<br>1<br>1<br>1<br>1	CG7052	TepII	12	6961 antibacterial humoral response (sensu Protostomia)	17114 wide-spectrum protease inhibitor activity	215 Serpin<br>1599 Alpha-2-macroglobulin<br>2890 Alpha-2-macroglobulin, N-terminal<br>8930 Terpenoid cylases/protein prenyltransferase alpha-alpha toroid
145971_at	CG7052-PD<br>CG7052-PC<br>CG7052-PE<br>CG7052-PA	1<br>1<br>1<br>1	CG7052	TepII	12	6961 antibacterial humoral response (sensu Protostomia)	17114 wide-spectrum protease inhibitor activity	215 Serpin<br>1599 Alpha-2-macroglobulin<br>2890 Alpha-2-macroglobulin, N-terminal<br>8930 Terpenoid cylases/protein prenyltransferase alpha-alpha

Affymetrix ID	Gene Product	Matching Score	Gene	Gene Name	cluster number	GeneOntology Biological Process	GeneOntology Molecular Function	InterPro domain
	CG7052-PB	1						toroid
146406_at	CG18563-PA	1	CG18563	CG18563	12			1254 Peptidase S1, chymotrypsin<br>9003 Peptidase, trypsin-like serine and cysteine proteases
152116_at	CG4559-PB<br>CG4559-PA<br>CG4559-PC	1<br>1<br>1	CG4559	Idgf3	12	7165 signal transduction<br>7267 cell-cell signaling<br>7444 imaginal disc development	8084 imaginal disc growth factor activity<br>16799 hydrolase activity, hydrolyzing N-glycosyl compounds	1223 Glycoside hydrolase, family 18
151552_s_at	CG30090-PA	1	CG30090	CG30090	12	6508 proteolysis and peptidolysis<br>6520 amino acid metabolism<br>6521 regulation of amino acid metabolism<br>8652 amino acid biosynthesis<br>19538 protein metabolism	8233 peptidase activity<br>8236 serine-type peptidase activity	1254 Peptidase S1, chymotrypsin<br>1314 Peptidase S1A, chymotrypsin<br>9003 Peptidase, trypsin-like serine and cysteine proteases
151551_i_at	CG30090-PA	0.79	CG30090	CG30090	12	6508 proteolysis and peptidolysis<br>6520 amino acid metabolism<br>6521 regulation of amino acid metabolism<br>8652 amino acid biosynthesis<br>19538 protein metabolism	8233 peptidase activity<br>8236 serine-type peptidase activity	1254 Peptidase S1, chymotrypsin<br>1314 Peptidase S1A, chymotrypsin<br>9003 Peptidase, trypsin-like serine and cysteine proteases
141527_at	CG4821-PA<br>CG4821-PB<br>CG4821-PD	1<br>1<br>1	CG4821	Tequila	12	6508 proteolysis and peptidolysis	4252 serine-type endopeptidase activity<br>4295 trypsin activity<br>8132 pancreatic elastase activity	194 H+-transporting two-sector ATPase, alpha/beta subunit, central region<br>1190 Speract/scavenger receptor<br>1254 Peptidase S1, chymotrypsin<br>1314 Peptidase S1A, chymotrypsin<br>2172 Low density lipoprotein-receptor, class A<br>2557 Chitin binding Peritrophin-A<br>9003 Peptidase, trypsin-like serine and cysteine proteases
148446_at	CG4821-PA<br>CG4821-PB<br>CG4821-PD	1<br>1<br>1	CG4821	Tequila	12	6508 proteolysis and peptidolysis	4252 serine-type endopeptidase activity<br>4295 trypsin activity<br>8132 pancreatic elastase activity	194 H+-transporting two-sector ATPase, alpha/beta subunit, central region<br>1190 Speract/scavenger receptor<br>1254 Peptidase S1, chymotrypsin<br>1314 Peptidase S1A, chymotrypsin<br>2172 Low density lipoprotein-receptor, class A<br>2557 Chitin binding Peritrophin-A<br>9003 Peptidase, trypsin-like serine and cysteine proteases
141436_at	CG3505-PA	1	CG3505	CG3505	12			
142657_at	CG9681-PA	1	CG9681	PGRP-SB1	12	6952 defense response<br>6955 immune response	16019 peptidoglycan receptor activity<br>4872 receptor activity	2502 N-acetylmuramoyl-L-alanine amidase, family 2<br>6619 Animal peptidoglycan recognition protein PGRP
144227_at	CG15293-PA	1	CG15293	BG:DS01759.2	12			
152301_at	CG12918-PA	1	CG12918	CG12918	12			886 Endoplasmic reticulum targeting sequence<br>8139 Saposin B
154182_at	CG5520-PA	1	CG5520	Gp93	12	6457 protein folding<br>6461 protein complex assembly<br>6950 response to stress<br>6952 defense response	51082 unfolded protein binding	886 Endoplasmic reticulum targeting sequence<br>1404 Heat shock protein Hsp90<br>3594 ATP-binding region, ATPase-like<br>9079 Four-helical cytokine
142206_s_at	CG14527-PA	1	CG14527	CG14527*	12	6508 proteolysis and peptidolysis	4222 metalloendopeptidase activity	718 Peptidase M13, neprilysin<br>6025 Peptidase M, neutral zinc metallopeptidases, zinc-binding site<br>8753 Peptidase M13
	CG14526-PA	1	CG14526	BcDNA:GH07188		6508 proteolysis and peptidolysis<br>7165 signal transduction	16511 endothelin-converting enzyme activity	718 Peptidase M13, neprilysin<br>6025 Peptidase M, neutral zinc metallopeptidases, zinc-binding site<br>8753 Peptidase M13
151496_s_at	CG14526-PA	1	CG14526	BcDNA:GH07188	12	6508 proteolysis and peptidolysis<br>7165 signal transduction	16511 endothelin-converting enzyme activity	718 Peptidase M13, neprilysin<br>6025 Peptidase M, neutral zinc metallopeptidases, zinc-binding site<br>8753 Peptidase M13
154589_at	CG31918-PA	1	CG31918	CG31918	12			
147135_at	CG4670-PA	1	CG4670	CG4670	12	6464 protein modification	15926 glucosidase activity<br>16971 flavin-linked sulfhydryl oxidase activity	6662 Thioredoxin type domain<br>6663 Thioredoxin domain 2<br>6863 Erv1/Alr

Affymetrix ID	Gene Product	Matching Score	Gene	Gene Name	cluster number	GeneOntology Biological Process	GeneOntology Molecular Function	InterPro domain
145731_at	CG16712-PA	1	CG16712	CG16712	12		4867 serine-type endopeptidase inhibitor activity	2223 Pancreatic trypsin inhibitor (Kunitz)
153098_at	CG16705-PA	1	CG16705	CG16705	12	6508 proteolysis and peptidolysis	4295 trypsin activity	1254 Peptidase S1, chymotrypsin
						6521 regulation of amino acid metabolism	8439 monophenol monooxygenase activator activity	1314 Peptidase S1A, chymotrypsin
						8652 amino acid biosynthesis		2919 Trypsin inhibitor-like, cysteine-rich TIL region
								9003 Peptidase, trypsin-like serine and cysteine proteases
147139_at	CG10799-PA	1	CG10799	CG10799	12			
146503_at	CG10363-PA	1	CG10363	TepIV	12	6961 antibacterial humoral response (sensu Protostomia)	30414 protease inhibitor activity	1599 Alpha-2-macroglobulin
								2114 HPr serine phosphorylation site
								2890 Alpha-2-macroglobulin, N-terminal
								8930 Terpenoid cylases/protein prenyltransferase alpha-alpha toroid
145735_at	CG3410-PA	1	CG3410	lectin-24A	12	7283 spermatogenesis	5534 galactose binding	1304 C-type lectin
144274_at	CG31780-PA	0.57	CG31780	CG31780*	12	6508 proteolysis and peptidolysis	8047 enzyme activator activity	1254 Peptidase S1, chymotrypsin
	CG31780-PB	1				6520 amino acid metabolism	8233 peptidase activity	1314 Peptidase S1A, chymotrypsin
						6521 regulation of amino acid metabolism	8236 serine-type peptidase activity	9003 Peptidase, trypsin-like serine and cysteine proteases
						8652 amino acid biosynthesis	30234 enzyme regulator activity	
						19538 protein metabolism		
	CG18477-PA	1	CG18477	BG:DS07108.1	12		4252 serine-type endopeptidase activity	1254 Peptidase S1, chymotrypsin
							8047 enzyme activator activity	1314 Peptidase S1A, chymotrypsin
								9003 Peptidase, trypsin-like serine and cysteine proteases
149884_at	CG9616-PA	1	CG9616	CG9616	12			
150780_at	CG9989-PA	1	CG9989	CG9989	12			1604 DNA/RNA non-specific endonuclease
144225_at	CG18478-PA	1	CG18478	BG:DS07108.5*	12			1254 Peptidase S1, chymotrypsin
								1314 Peptidase S1A, chymotrypsin
								9003 Peptidase, trypsin-like serine and cysteine proteases
	CG31827-PA	1	CG31827	CG31827		6508 proteolysis and peptidolysis	4252 serine-type endopeptidase activity	1254 Peptidase S1, chymotrypsin
	CG31827-PB	1				6520 amino acid metabolism	8047 enzyme activator activity	1314 Peptidase S1A, chymotrypsin
						6521 regulation of amino acid metabolism	8233 peptidase activity	9003 Peptidase, trypsin-like serine and cysteine proteases
						8652 amino acid biosynthesis	8236 serine-type peptidase activity	
						19538 protein metabolism	30234 enzyme regulator activity	
144318_at	CG18096-PA	1	CG18096	TepI	12	6961 antibacterial humoral response (sensu Protostomia)		345 Cytochrome c heme-binding site
								1599 Alpha-2-macroglobulin
								2890 Alpha-2-macroglobulin, N-terminal
								8930 Terpenoid cylases/protein prenyltransferase alpha-alpha toroid
145620_at	CG4259-PA	1	CG4259	CG4259	12			1254 Peptidase S1, chymotrypsin
								1314 Peptidase S1A, chymotrypsin
								9003 Peptidase, trypsin-like serine and cysteine proteases
142931_at	CG14225-PA	1	CG14225	CG14225	13		4872 receptor activity	3529 Long hematopoietin receptor, gp130 family 2
147547_at	CG18067-PA	1	CG18067	CG18067	13			2073 3'5'-cyclic nucleotide phosphodiesterase
144238_at	CG5700-PB	1	CG5700	prc	14	7507 heart development		9765 Pericardin like
						16477 cell migration		
						35088 establishment and/or maintenance of apical/basal cell polarity		
154048_at	CG5991-PC	1	CG5991	CG5991	14			3817 Phosphatidylserine decarboxylase-related
	CG5991-PB	1						5221 Phosphatidylserine decarboxylase
	CG5991-PA	1						
145809_at	CG31651-PA	0.79	CG31651	CG31651	14			772 Ricin B lectin domain
								1173 Glycosyl transferase, family 2
								8997 Ricin B-related lectin
147243_at	CG8160-PA	1	CG8160	CG8160	14			
147151_at	CG18279-PB	0.82	CG18279	CG18279	14	6952 defense response	5554 molecular_function unknown	
	CG18279-PA	0.89				8063 Toll signaling pathway		
						19731 antibacterial humoral response		
151137_r_at	CG15231-PA	1	CG15231	IM4	14	6952 defense response		
						6955 immune response		
151209_at	CG15065-PA	1	CG15065	CG15065	14			
151208_at	CG15068-PA	1	CG15068	CG15068	14			

Affymetrix ID	Gene Product	Matching Score	Gene	Gene Name	cluster number	GeneOntology Biological Process	GeneOntology Molecular Function	InterPro domain
151211_at	CG16844-PA	1	CG16844	CG16844	14	6952 defense response<br>8063 Toll signaling pathway<br>19731 antibacterial humoral response	5554 molecular_function unknown	
141578_at	CG17278-PA	1	CG17278	BcDNA:SD04019	14			2350 Serine protease inhibitor, Kazal type
143268_at	CG3297-PB<br>CG3297-PA<br>CG3297-PC	1<br>1<br>1	CG3297	mnd	14	6520 amino acid metabolism<br>6865 amino acid transport	15171 amino acid transporter activity	2293 Amino acid/polyamine transporter I<br>4841 Amino acid permease-associated region
153601_at	CG9888-PA	1	CG9888	Fib	14	6365 35S primary transcript processing<br>16072 rRNA metabolism	3723 RNA binding	692 Fibrillarin
146098_at	CG3949-PA	1	CG3949	hoip	14	398 nuclear mRNA splicing, via spliceosome<br>7399 neurogenesis<br>7422 peripheral nervous system development	3723 RNA binding<br>3735 structural constituent of ribosome	2415 High mobility group-like nuclear protein<br>4037 Ribosomal protein L7AE<br>4038 Ribosomal protein L7Ae/L30e/S12e/Gadd45
146963_at	CG7637-PA	1	CG7637	CG7637	14			7264 Nucleolar RNA-binding protein Nop10p
153819_at	CG7421-PB<br>CG7421-PC<br>CG7421-PA	1<br>1<br>1	CG7421	Nopp140	14	17126 nucleologenesis		5819 Histone H5
155019_at	CG12785-PA	1	CG12785	CG12785	14			209 Peptidase S8 and S53, subtilisin, kexin, sedolisin<br>5554 Nrap protein
152425_at	CG3174-PA	1	CG3174	Fmo-2	15	6520 amino acid metabolism	4499 dimethylaniline monooxygenase (N-oxide-forming) activity	759 Adrenodoxin reductase<br>960 Flavin-containing monooxygenase FMO<br>1100 Pyridine nucleotide-disulphide oxidoreductase, class I<br>1327 FAD-dependent pyridine nucleotide-disulphide oxidoreductase
141664_at	CG12101-PB<br>CG12101-PA	1<br>1	CG12101	Hsp60	15	6457 protein folding<br>6458 'de novo' protein folding<br>6626 protein-mitochondrial targeting<br>6950 response to stress<br>9408 response to heat<br>42026 protein refolding	42623 ATPase activity, coupled<br>51082 unfolded protein binding	1844 Chaperonin Cpn60<br>2423 Chaperonin Cpn60/TCP-1<br>8950 GroEL-like chaperone, ATPase
143198_at	CG1242-PA	1	CG1242	Hsp83	15	6457 protein folding<br>6461 protein complex assembly<br>6952 defense response<br>7015 actin filament organization<br>7098 centrosome cycle<br>7283 spermatogenesis<br>7465 R7 cell fate commitment<br>8293 torso signaling pathway<br>8360 regulation of cell shape<br>8595 determination of anterior/posterior axis, embryo<br>9408 response to heat<br>45187 regulation of circadian sleep/wake cycle, sleep	42623 ATPase activity, coupled	1404 Heat shock protein Hsp90<br>3594 ATP-binding region, ATPase-like<br>9079 Four-helical cytokine
143961_at	no matches				15			
150831_at	CG18111-PA	1	CG18111	Obp99a	15		5549 odorant binding	6170 Pheromone/general odorant binding protein, PBP/GOBP
152513_at	CG10513-PA	1	CG10513	CG10513	15			4119 Protein of unknown function DUF227
154821_at	CG8147-PA	1	CG8147	CG8147	16			1952 Alkaline phosphatase
