# Supplementary material for: Genome-wide gene expression in response to parasitoid attack in Drosophila
Source: Genome Biol. 2005 Oct 31;6(11):R94. doi: 10.1186/gb-2005-6-11-r94 (PMC1297650; doi:10.1186/gb-2005-6-11-r94)

Cluster 1

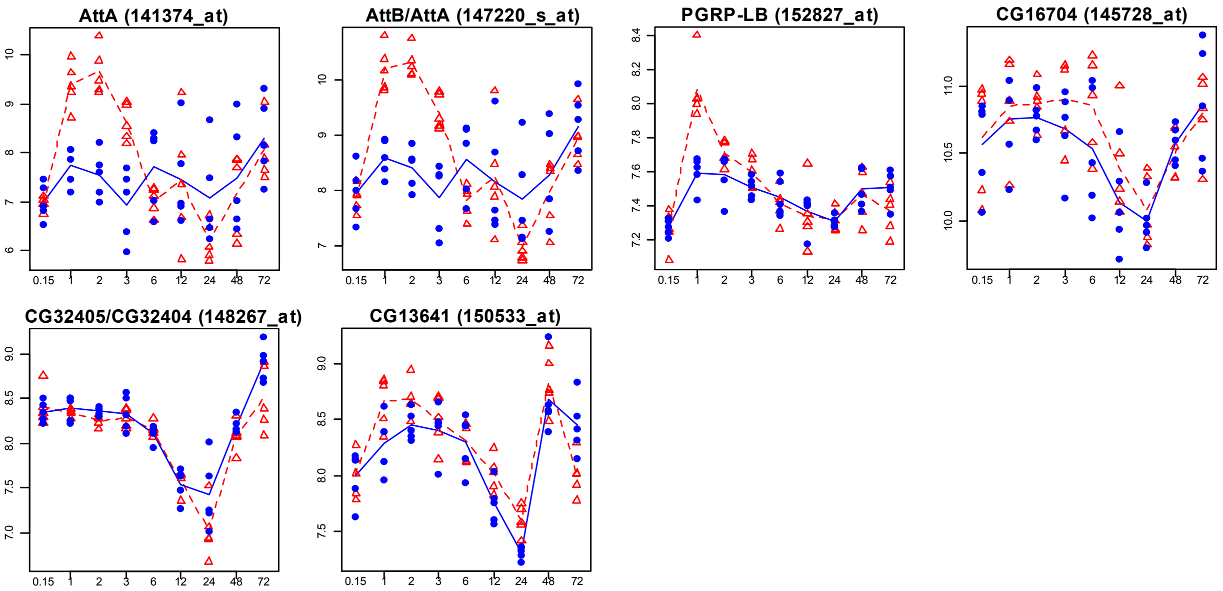

Colour legend:

CCARCAGRCCSA

CAWTSKATTC

AMTCAGT

NF-kappaB-like

MTTFA/SERPENT/GATA-like

STAT

TATA-like

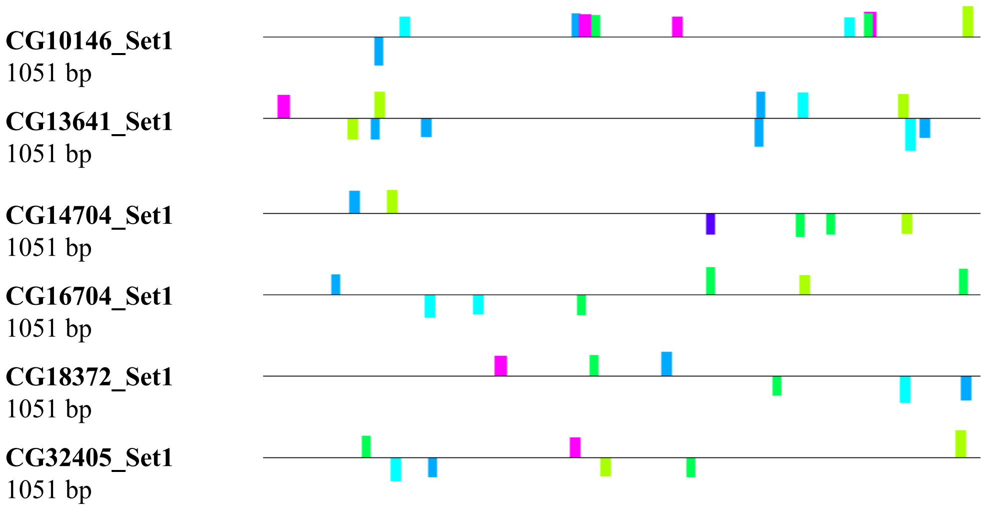

Supplemental Material:
Profiles & motifs per cluster
(Wertheim et al.)

Cluster 2

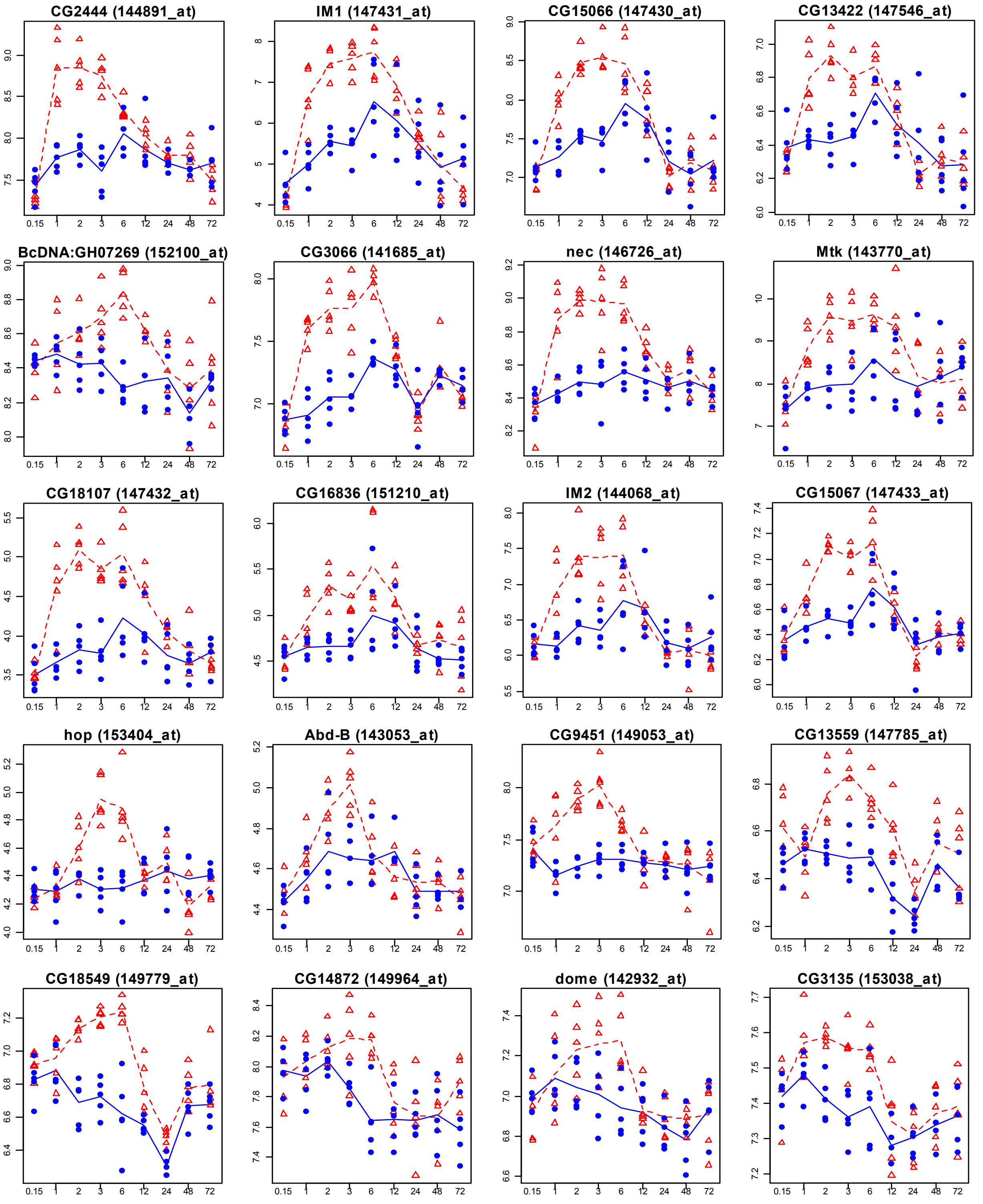

Supplemental Material:
Profiles & motifs per cluster
(Wertheim et al.)

Cluster 2 (continued)

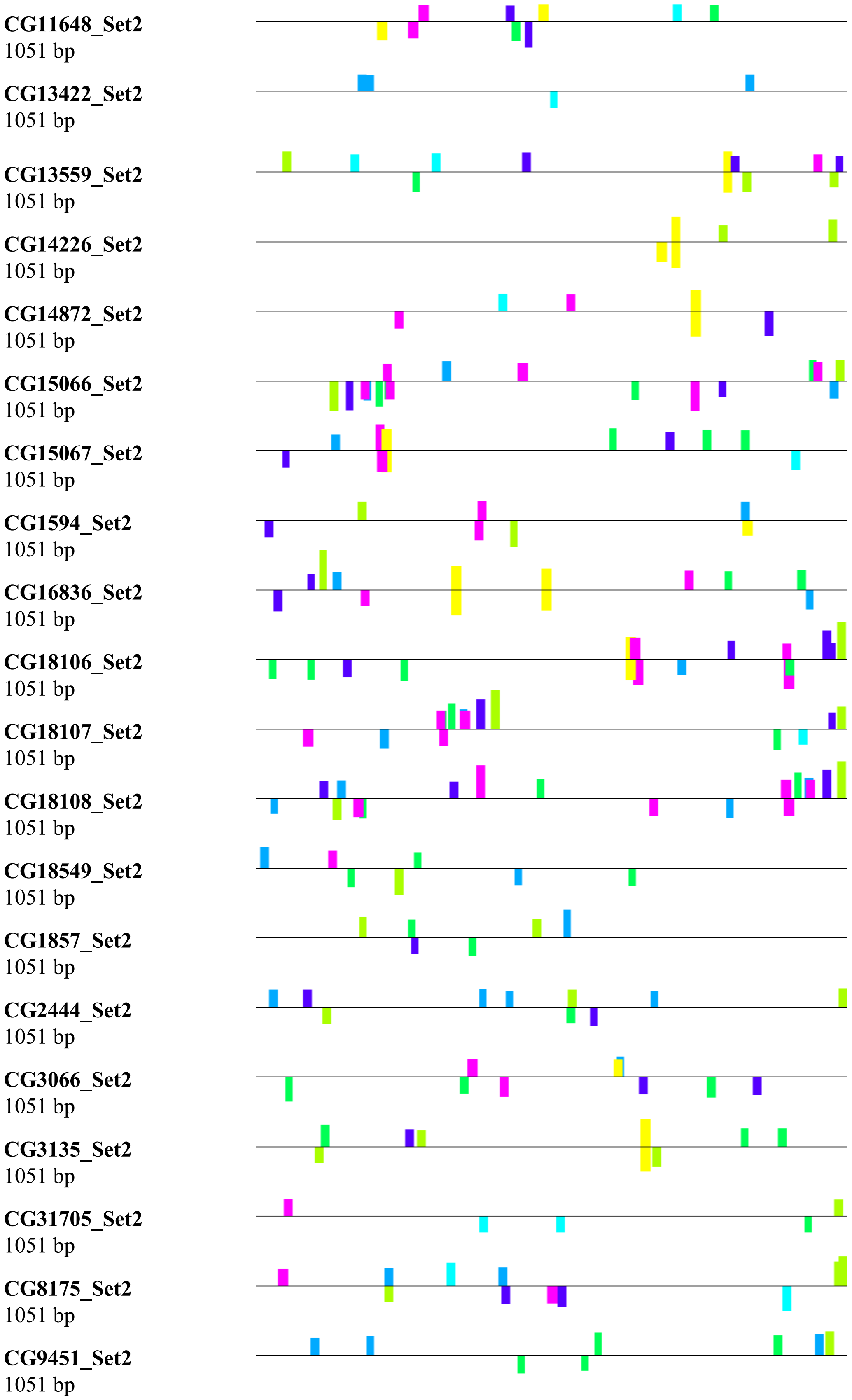

Cluster 3

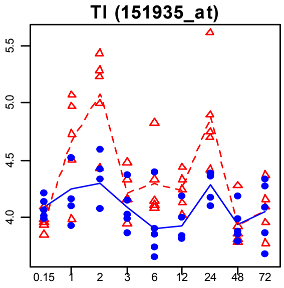

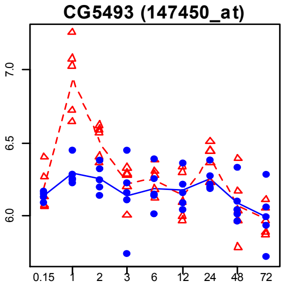

CG5490_Set3
1051 bp

CG5493_Set3
1051 bp

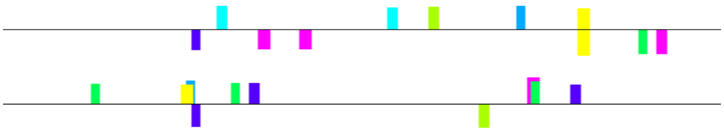

Cluster 4

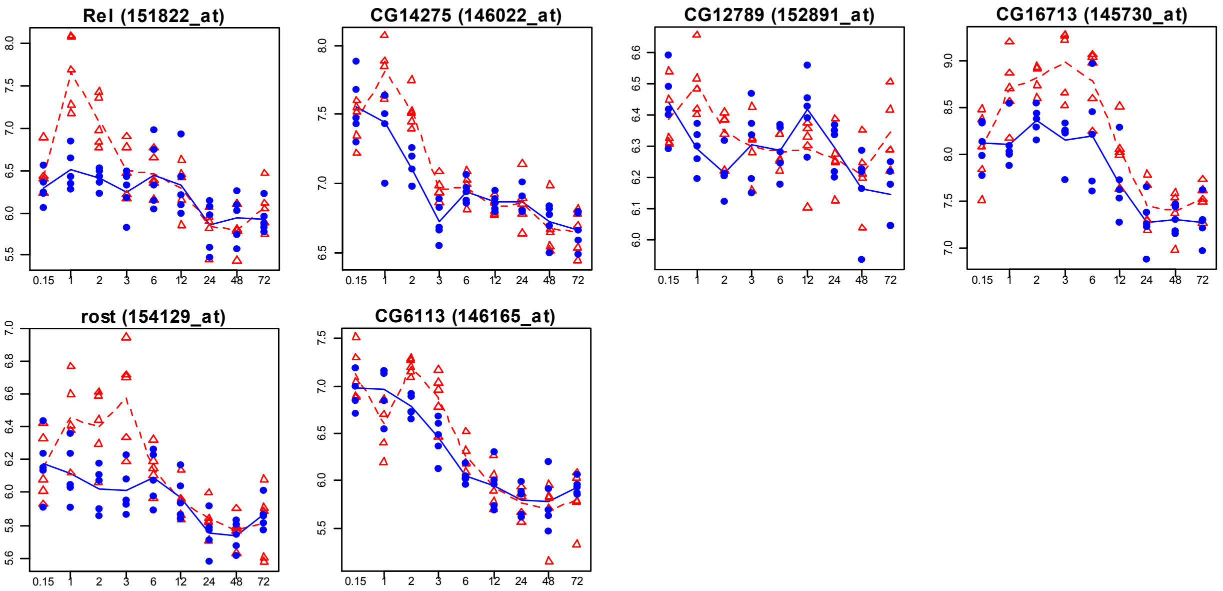

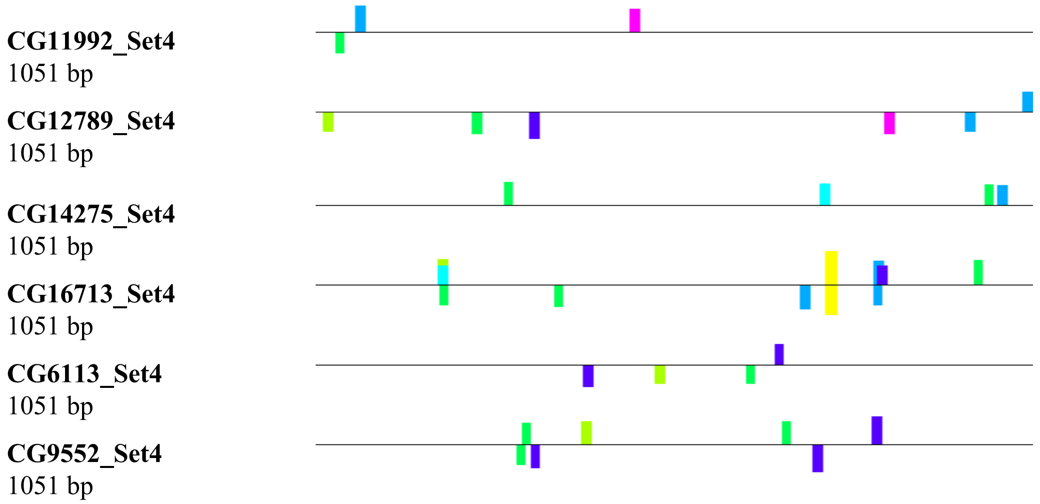

Supplemental Material:
Profiles & motifs per cluster
(Wertheim et al.)

Cluster 5

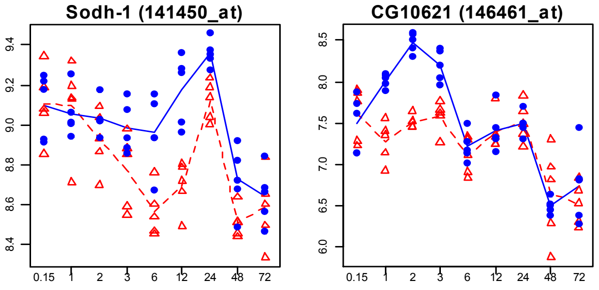

CG10621_Set5
1051 bp

CG1982_Set5
1051 bp

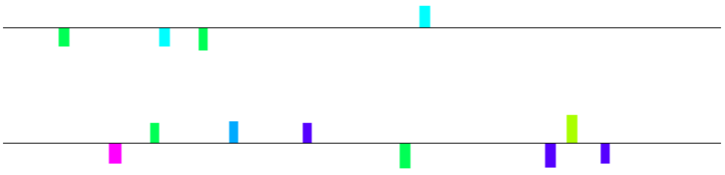

Supplemental Material:
Profiles & motifs per cluster
(Wertheim et al.)

Cluster 6

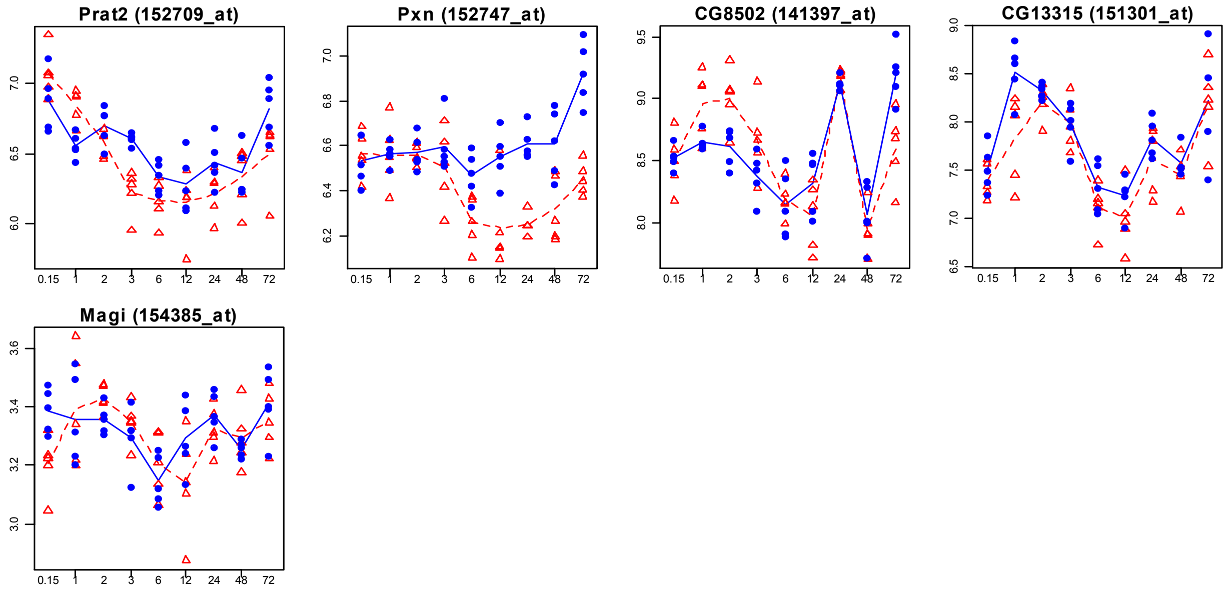

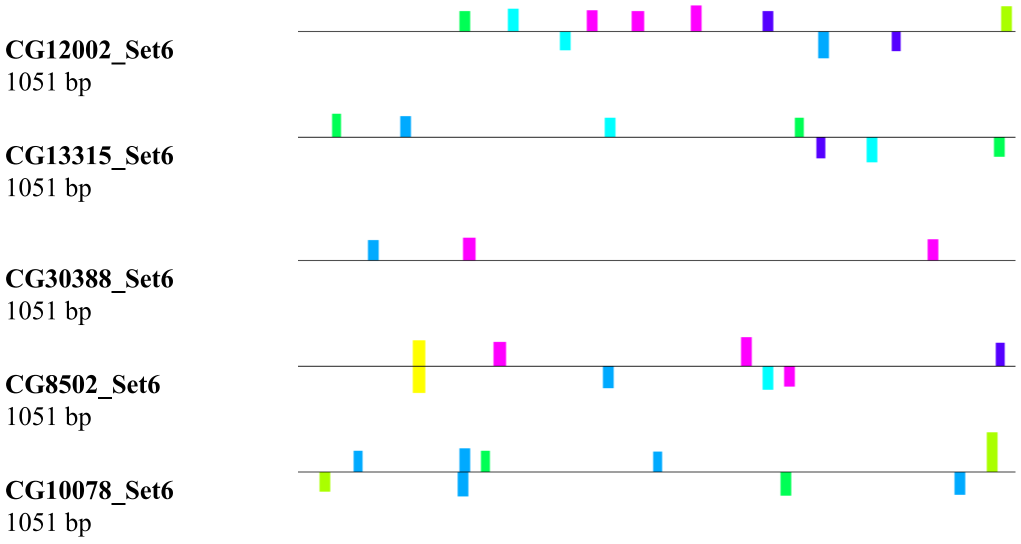

Supplemental Material:
Profiles & motifs per cluster
(Wertheim et al.)

Cluster 7

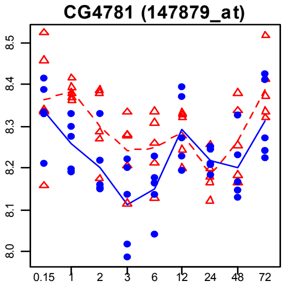

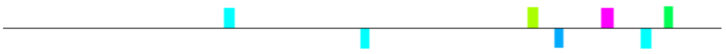

CG4781_Set7
1051 bp

Cluster 8

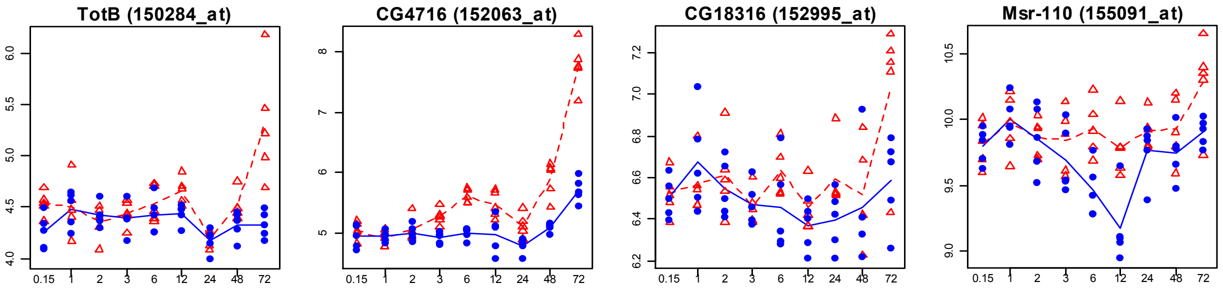
TotB (150284_at)
CG4716 (152063_at)
CG18316 (152995_at)
Msr-110 (155091_at)

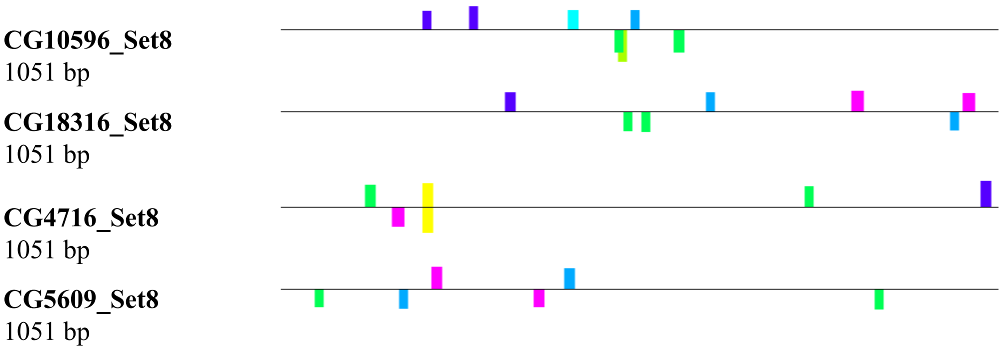
CG10596_Set8
1051 bp
CG18316_Set8
1051 bp
CG4716_Set8
1051 bp
CG5609_Set8
1051 bp

Cluster 9

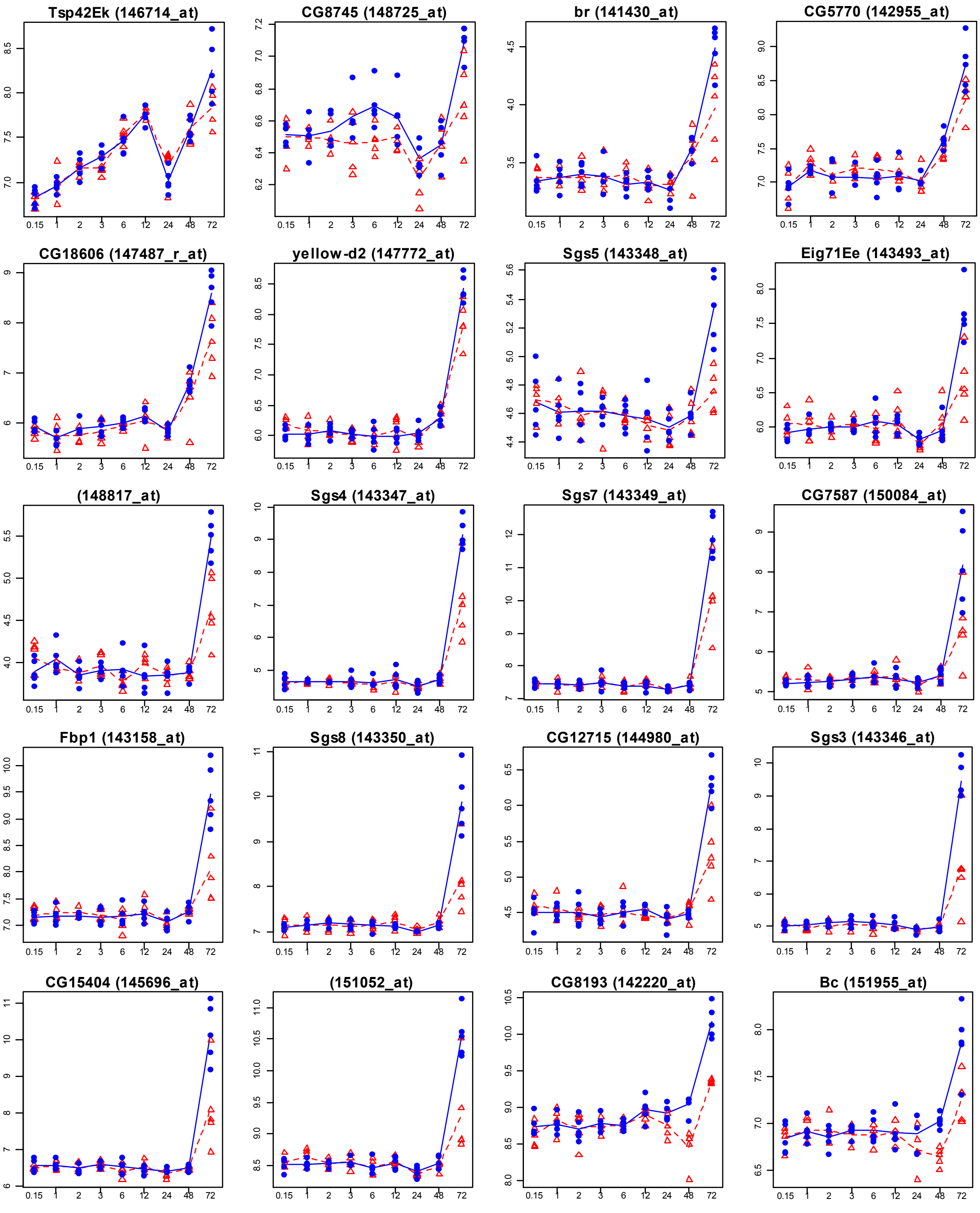

Supplemental Material:
Profiles & motifs per cluster
(Wertheim et al.)

Cluster 9 (continued)

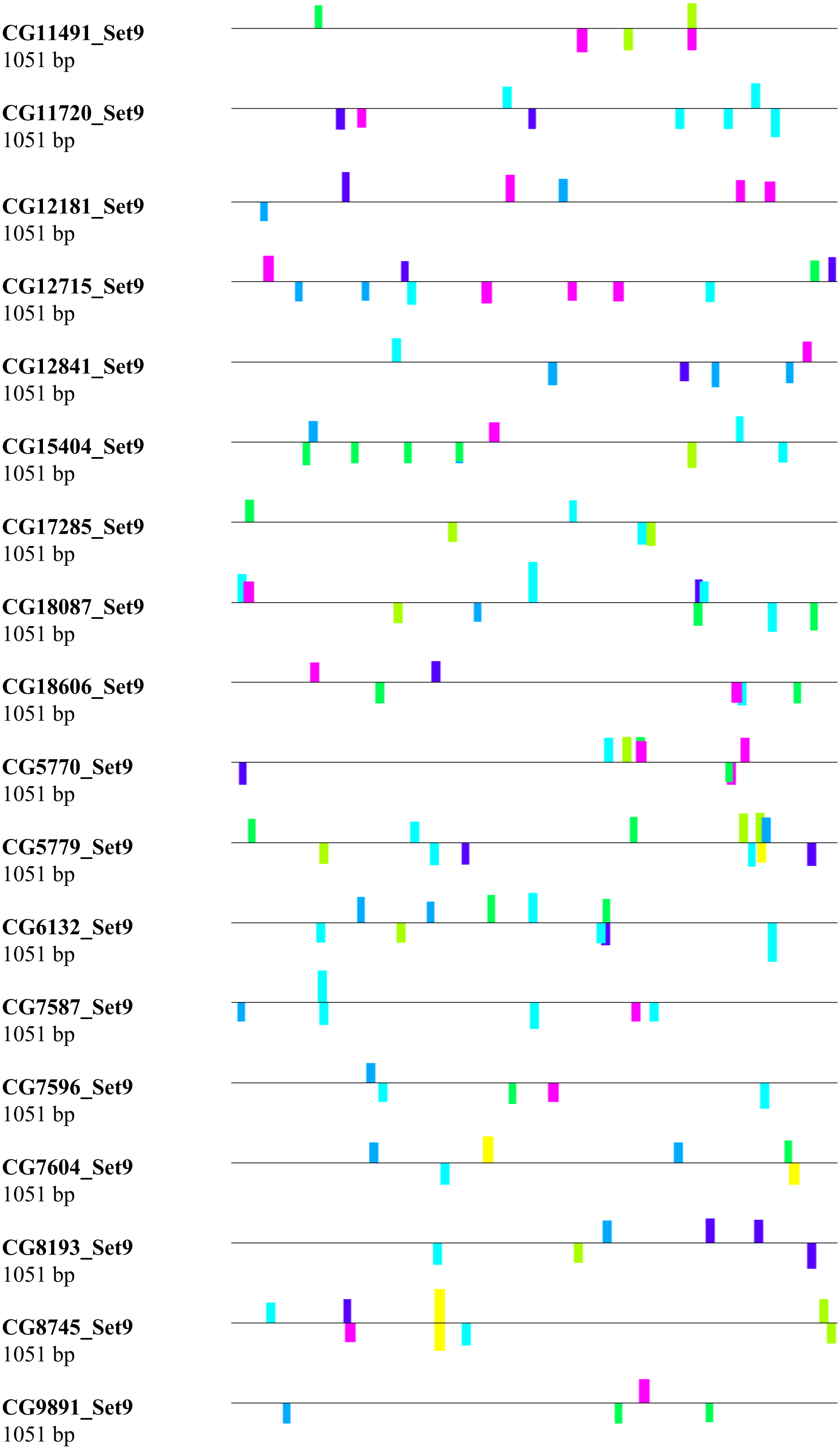

Supplemental Material:
Profiles & motifs per cluster
(Wertheim et al.)

Cluster 10

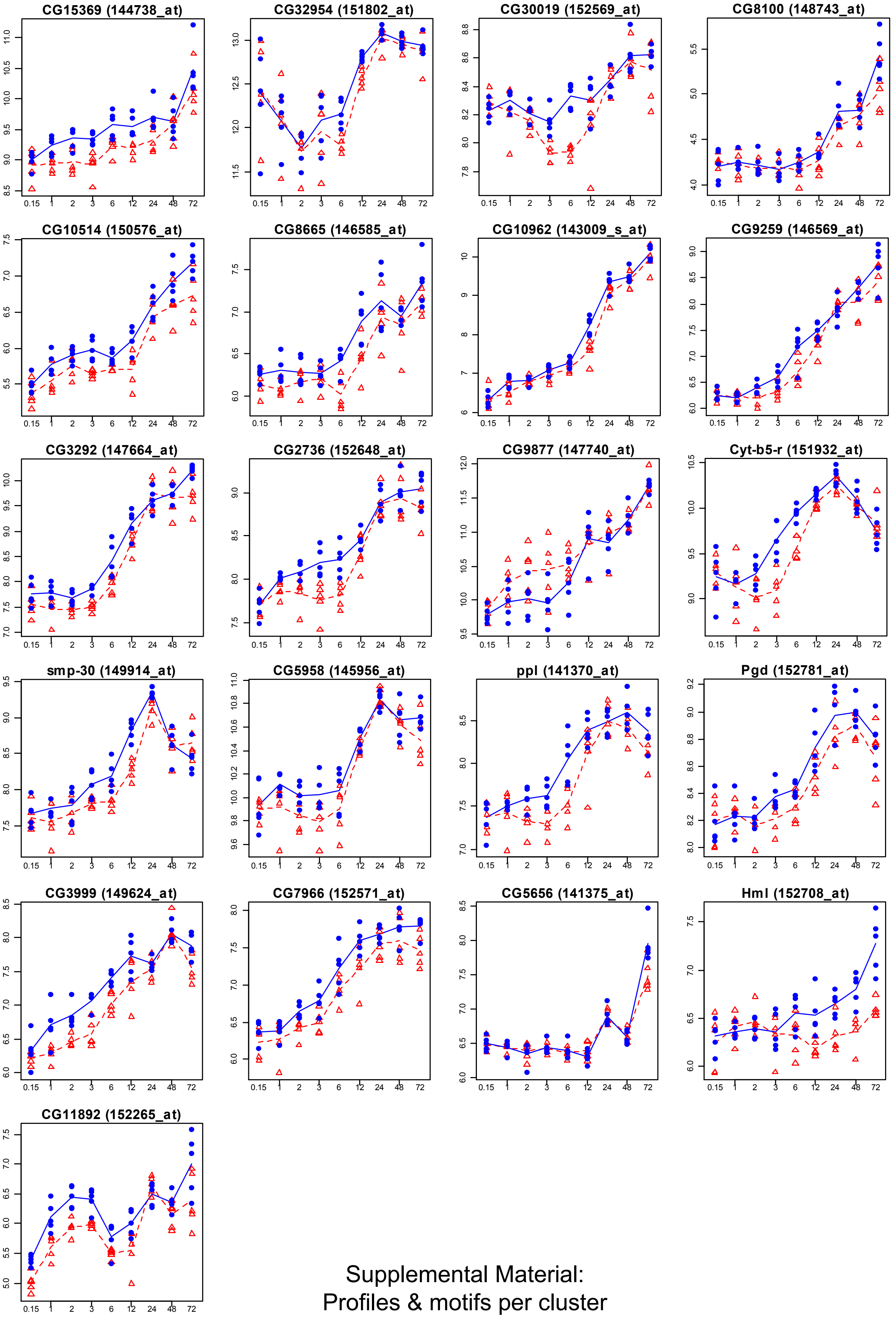

Supplemental Material:
Profiles & motifs per cluster
(Wertheim et al.)

Cluster 10 (continued)

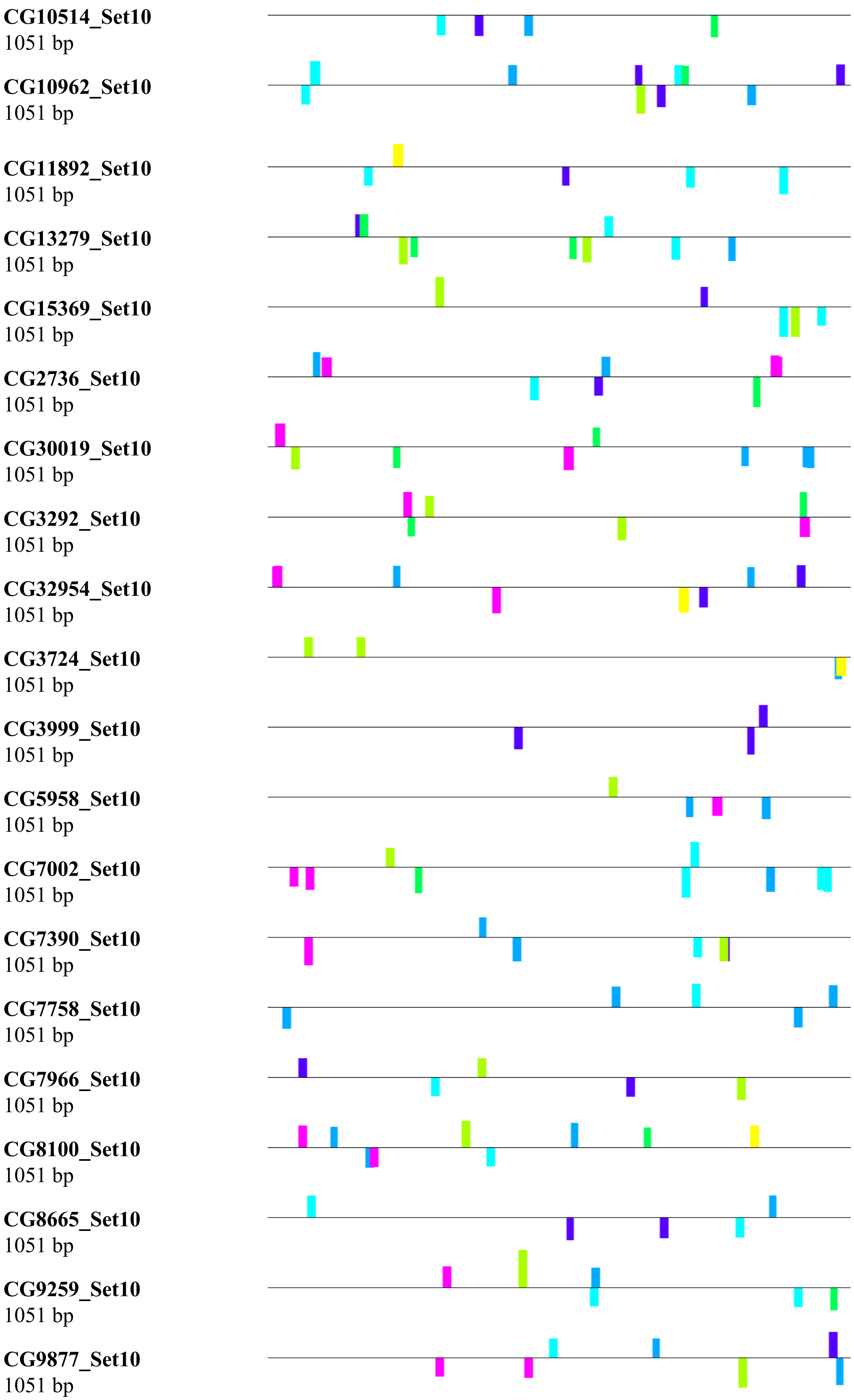

Supplemental Material:
Profiles & motifs per cluster
(Wertheim et al.)

Cluster 11

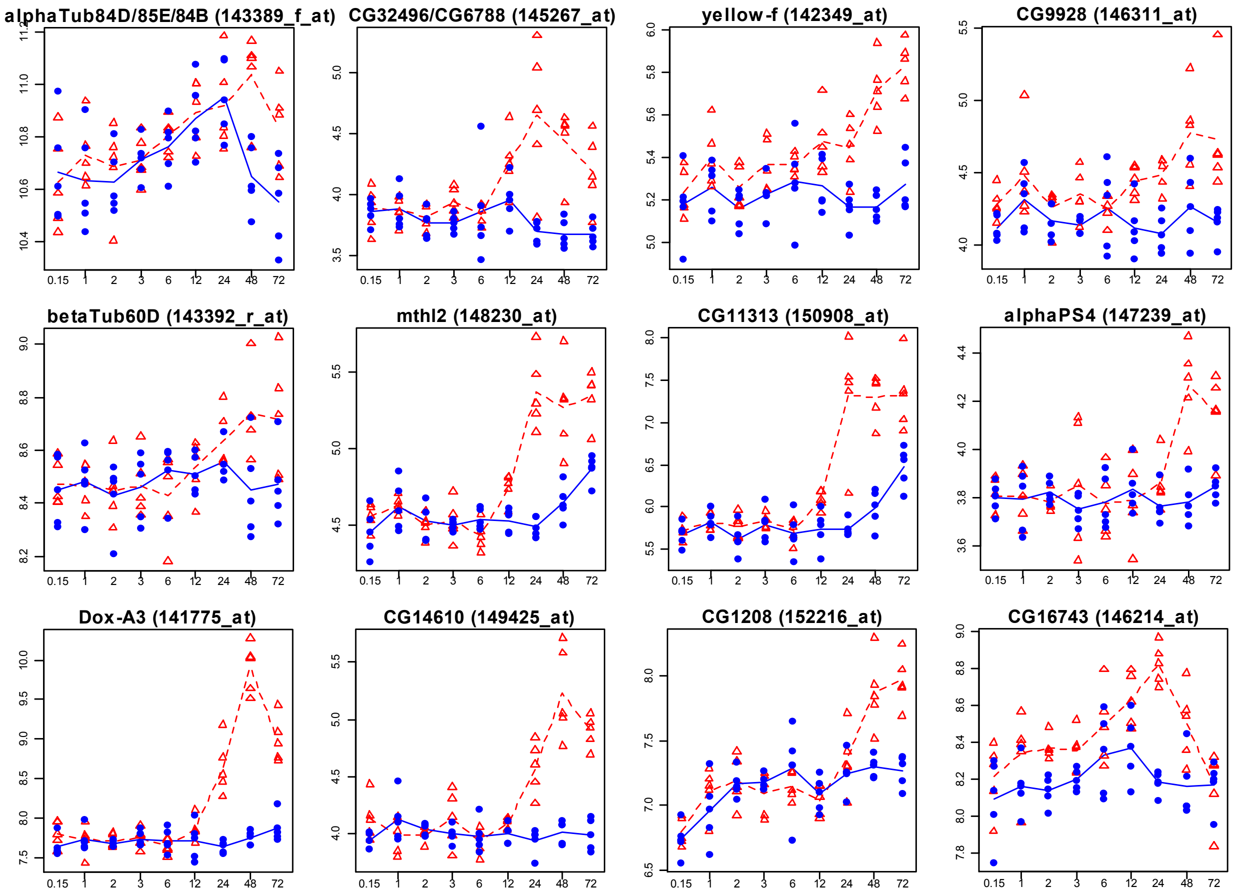

Supplemental Material:
Profiles & motifs per cluster
(Wertheim et al.)

Cluster 11 (continued)

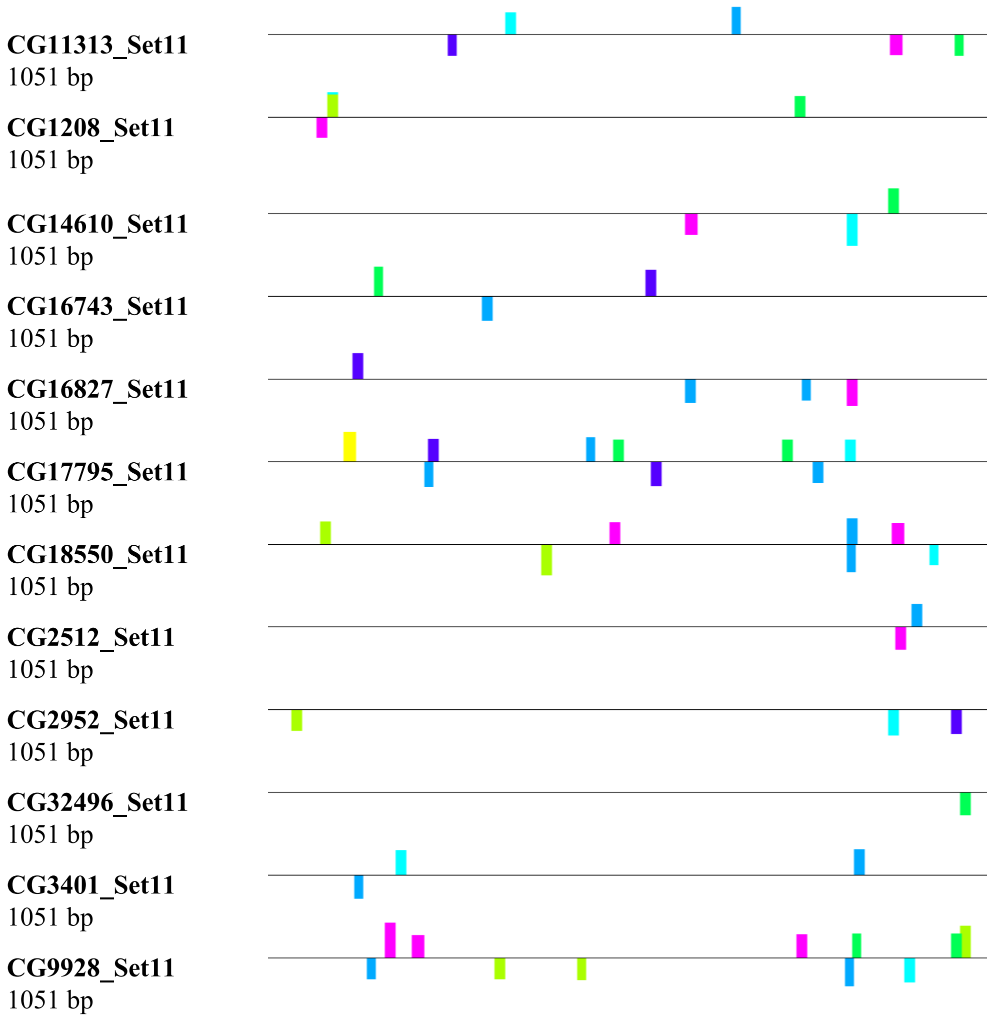

Cluster 12

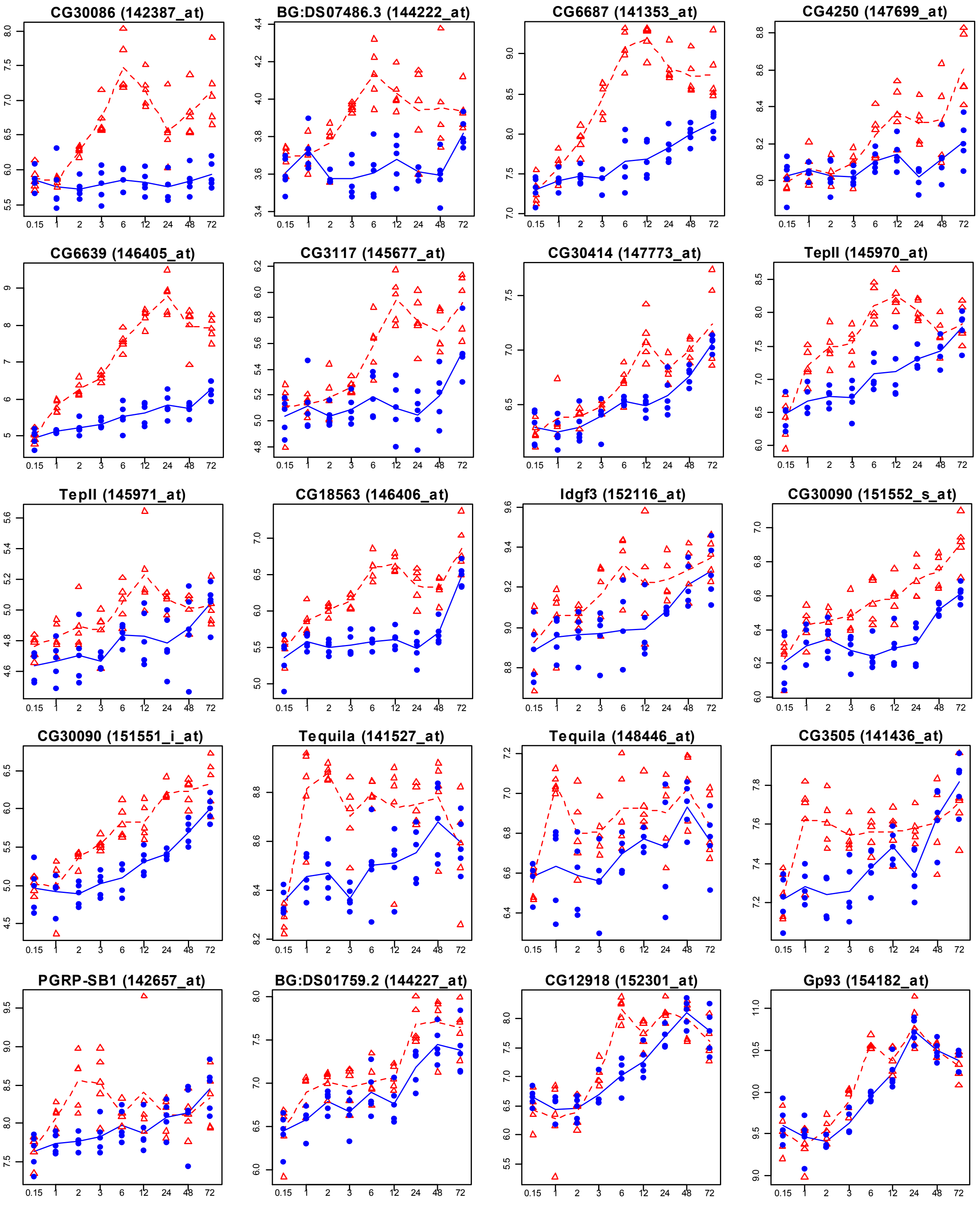

Supplemental Material:
Profiles & motifs per cluster
(Wertheim et al.)

Cluster 12 (continued)

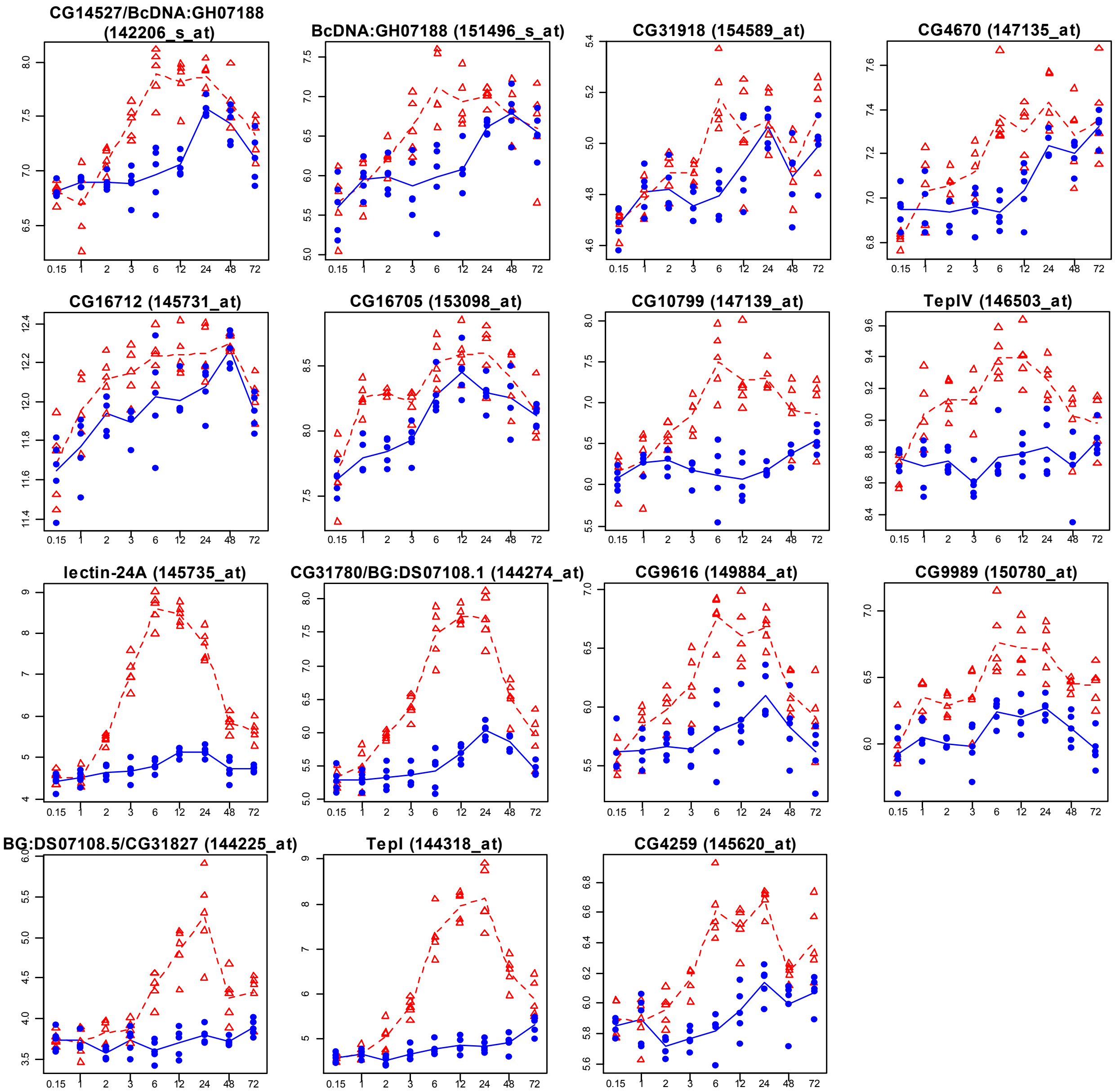

Supplemental Material:
Profiles & motifs per cluster
(Wertheim et al.)

Cluster 12 (continued)

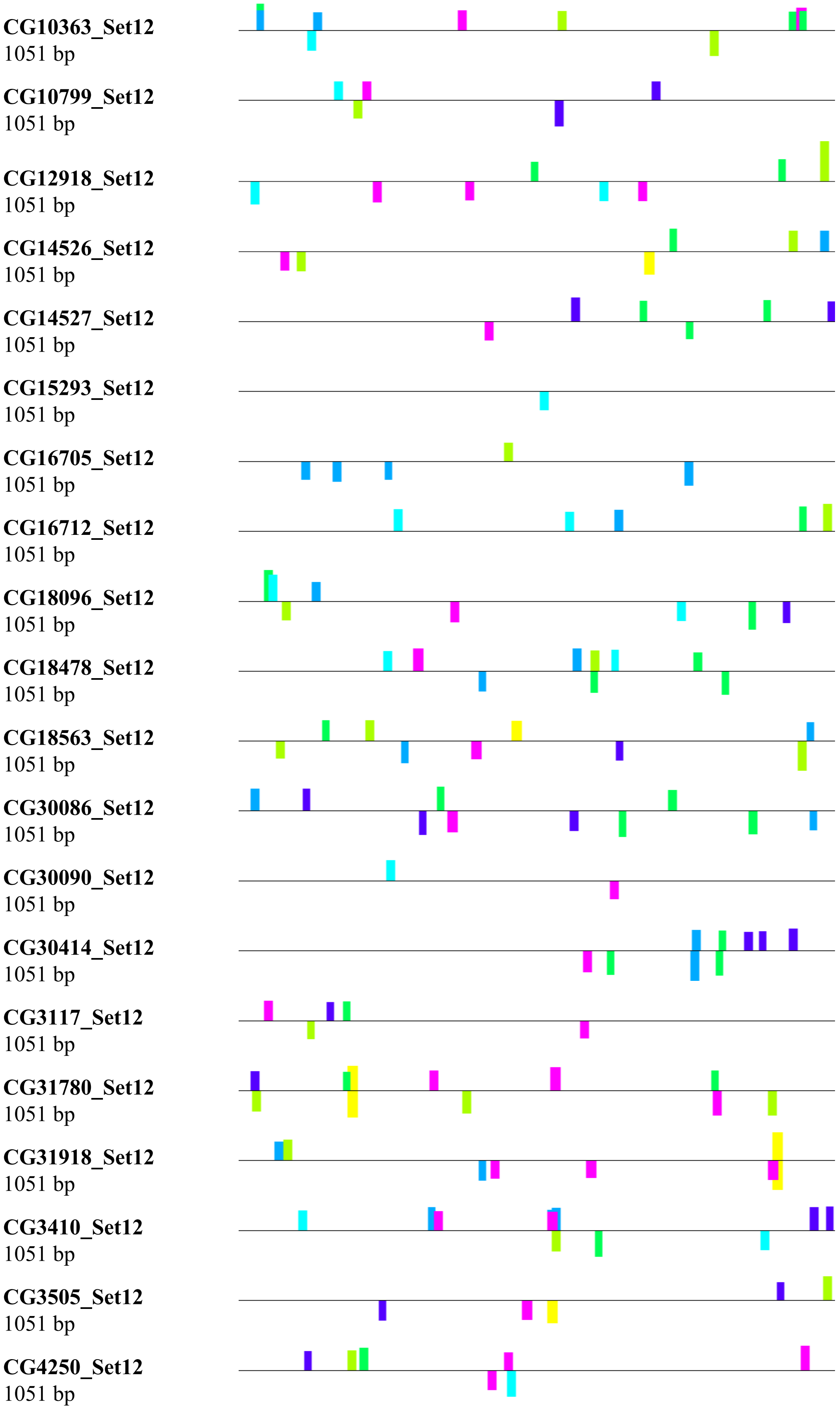

Supplemental Material:
Profiles & motifs per cluster
(Wertheim et al.)

Cluster 12 (continued)

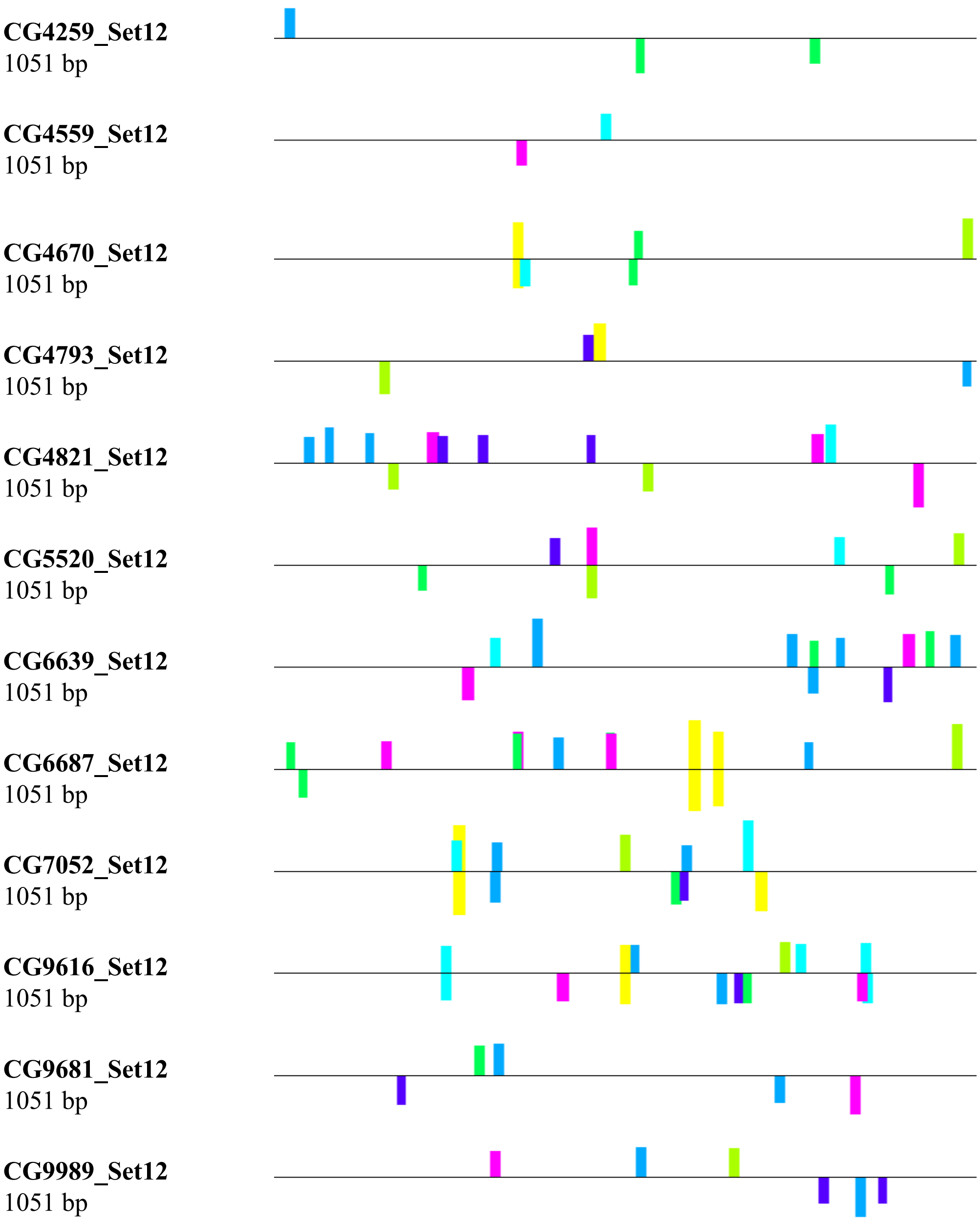

Cluster 13

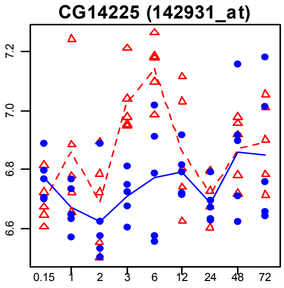

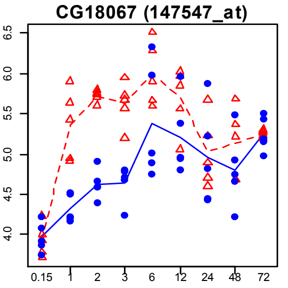

CG14225_Set13
1051 bp

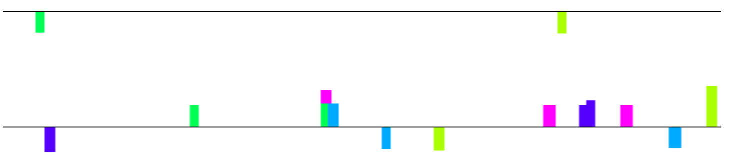

CG18067_Set13
1051 bp

Cluster 14

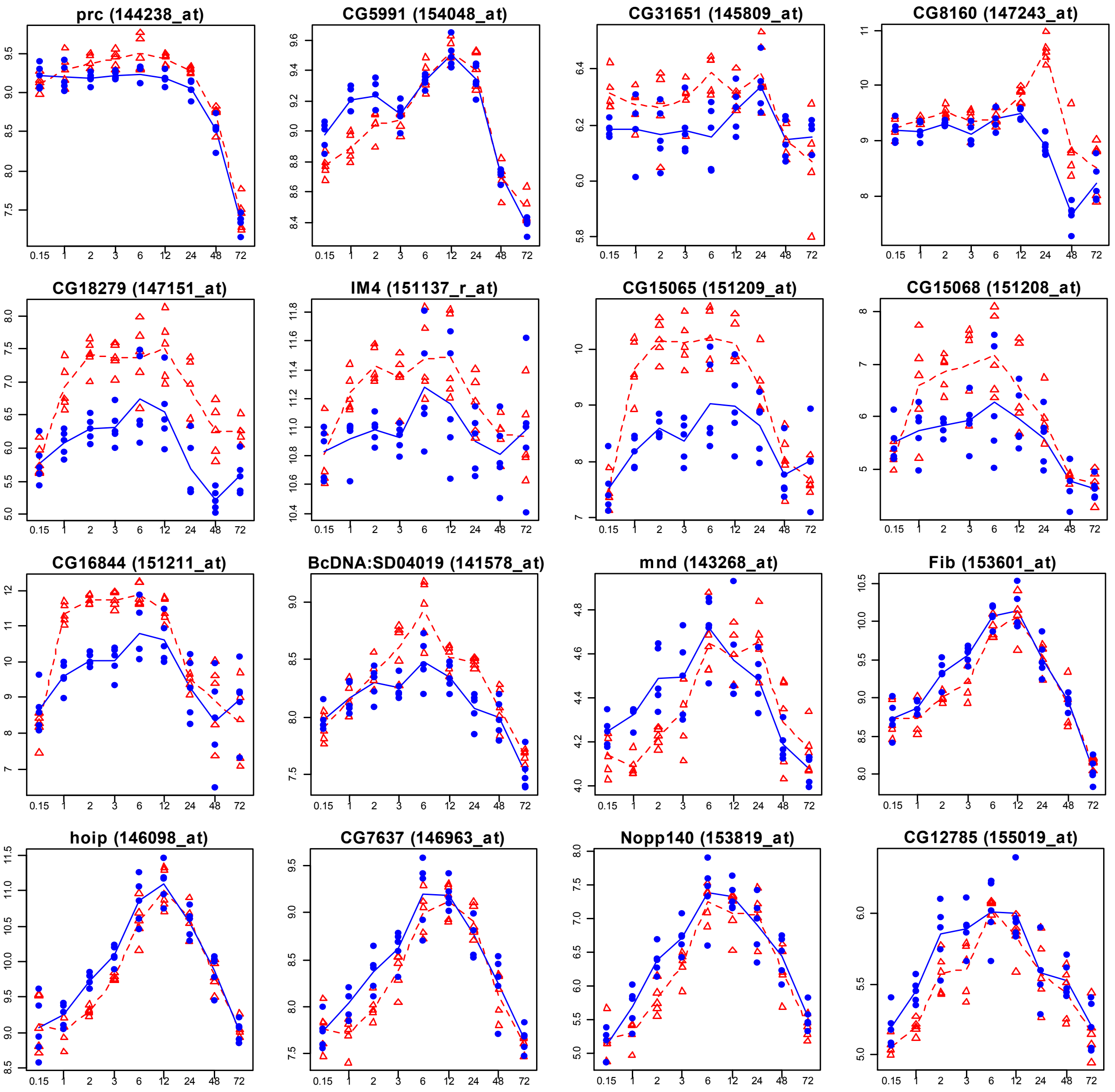
prc (144238_at)
CG5991 (154048_at)
CG31651 (145809_at)
CG8160 (147243_at)
CG18279 (147151_at)
IM4 (151137_r_at)
CG15065 (151209_at)
CG15068 (151208_at)
CG16844 (151211_at)
BcDNA:SD04019 (141578_at)
mnd (143268_at)
Fib (153601_at)
hoip (146098_at)
CG7637 (146963_at)
Nopp140 (153819_at)
CG12785 (155019_at)
0.15 1 2 3 6 12 24 48 72

Cluster 14 (continued)

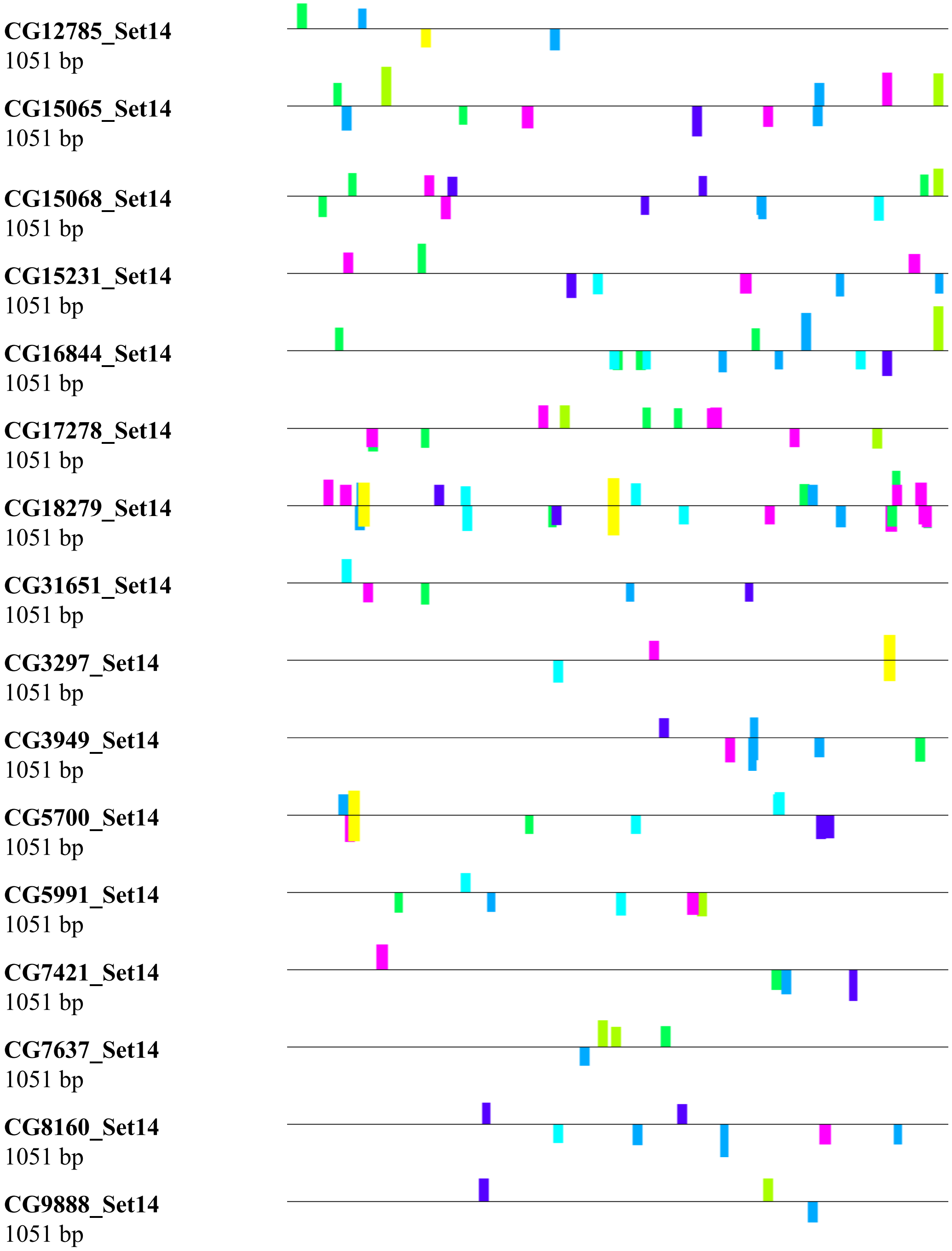

Cluster 15

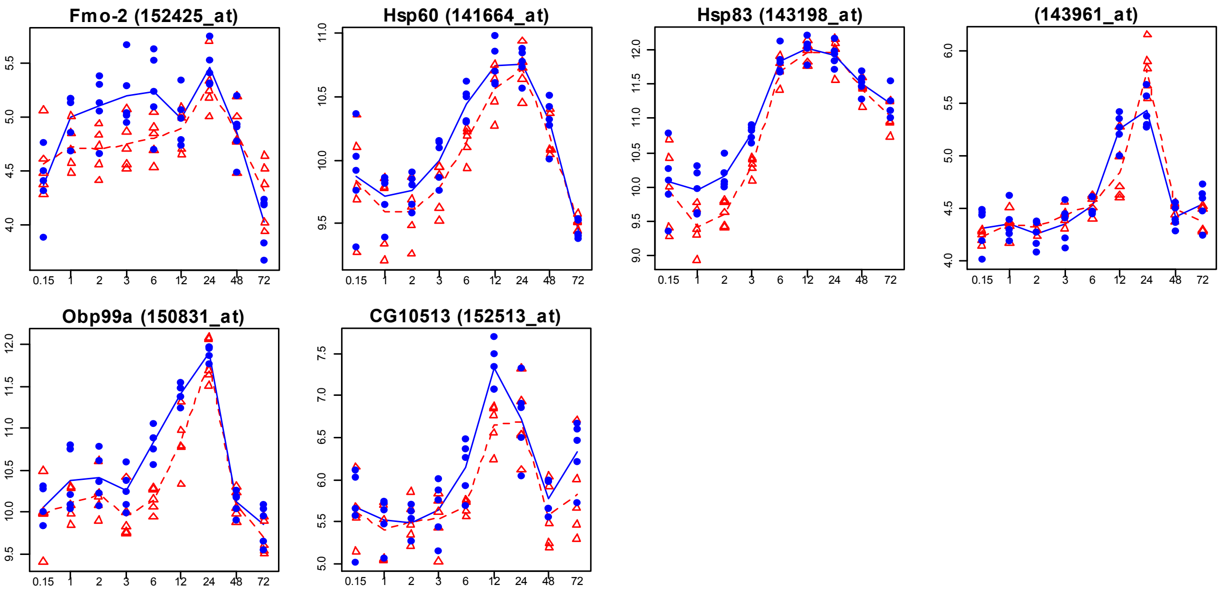
Fmo-2 (152425_at)
Hsp60 (141664_at)
Hsp83 (143198_at)
(143961_at)
Obp99a (150831_at)
CG10513 (152513_at)
0.15 1 2 3 6 12 24 48 72

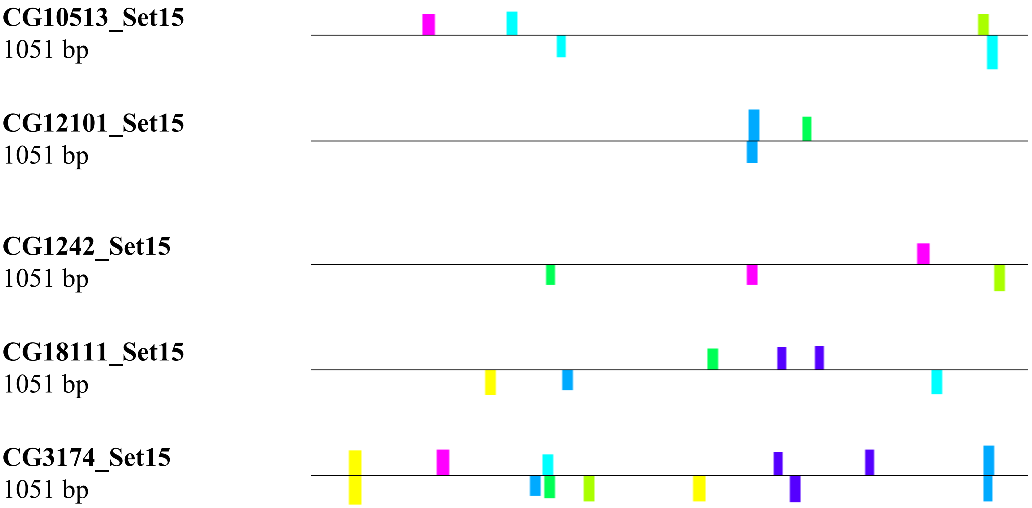
CG10513_Set15
1051 bp
CG12101_Set15
1051 bp
CG1242_Set15
1051 bp
CG18111_Set15
1051 bp
CG3174_Set15
1051 bp

Cluster 16

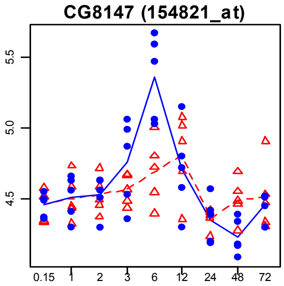

CG8147_Set16
1051 bp

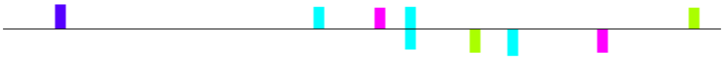

Supplement: Additional data file 2 — Expression profile and upstream motifs of all genes per cluster. The log2 expression values at the time points (hours) after parasitoid attack are shown for all replicates (blue circles for the control larvae; red triangles for the parasitized larvae) and the lines denote the average expression at each time point. The strength of match for the putative regulatory motifs in the upstream sequences is indicated by the height of the bars. [file gb-2005-6-11-r94-S2.pdf]
