# Supplementary material for: Genome-wide gene expression in response to parasitoid attack in Drosophila
Source: Genome Biol. 2005 Oct 31;6(11):R94. doi: 10.1186/gb-2005-6-11-r94 (PMC1297650; doi:10.1186/gb-2005-6-11-r94)
Supplement: Additional data file 3 — A full list of putative regulatory motifs that were significantly over-represented in our clusters of genes. Motifs were identified by MotifRegressor and over-representation was calculated in Clover. The Representative Motifs denote the degenerate motifs, using IUPAC Ambiguous DNA Characters. The Raw Score measures the strength of match and the frequency of occurrence. Significance (denoted in the last two columns) is based on the comparison with the upstream sequences of 1,000 randomly chosen genes represented on the Affymetrix Drosophila 1 Genome Array, respectively - all genes on Drosophila chromosome 2. [file gb-2005-6-11-r94-S3.pdf]

All Genes

Representative Motif	Motif	Raw Score	P-value for promoter background	P-value for chr 2R
CCARCAGRCCSA	TSGGYCTGCTGGC*GCCAGCAGRCCSA*BW_3hr.allmtfs	121	0	0
CCARCAGRCCSA	KYTCGGYCTGYTGGC*GCCARCAGRCCGARM*BW_3hr.allmtfs	114	0	0
CCARCAGRCCSA	TKYTSGGYCTGYTGG*CCARCAGRCCSARMA*BW_2hr.allmtfs	103	0	0
CCARCAGRCCSA	YTCGGYYTGYTSGC*GCSARCARRCCGAR*BW_6hr.allmtfs	97.6	0	0
CCARCAGRCCSA	TCGGYYTGYTSG*CSARCARRCCGA*BW_3hr.allmtfs	87.4	0	0
CCARCAGRCCSA	STGCTSGSYCTG*CAGRSCSAGCAS*BW_3hr.allmtfs	87.2	0	0
CCARCAGRCCSA	KTSTKYTSGSTCTG*CAGASCSARMASAM*BW_1hr.allmtfs	73.6	0	0
CCARCAGRCCSA	TSTKCTSGSYCTG*CAGRSCSAGMASA*BW_2hr.allmtfs	68.1	0	0
CCARCAGRCCSA	TSTGYTCGSTCTG*CAGASCGARCASA*BW_1hr.allmtfs	66.7	0	0
NF-kappaB-like	GGRRAATYCCAA*TTGGRATTYYCC*BW_1hr.allmtfs	54.2	0	0
NF-kappaB-like	GGAAAWTYCCA*TGGRAWTTTCC*BW_3hr.allmtfs	53.5	0	0
	ATCATKTTTKT*AMAAAMATGAT*BW_3hr.allmtfs	52	0.009	0.004
CAWTSKATTC	AGMAATMSAWTGAA*TTCAWTSKATTKCT*BW_2hr.allmtfs	51	0	0
CCARCAGRCCSA	TCGSCTTGYKGG*CCMRCAAGSCGA*BW_6hr.allmtfs	50.2	0	0
CCARCAGRCCSA	TTCGSCTTGTTSG*CSAACAAGSCGAA*BW_6hr.allmtfs	48.9	0	0
	TCGSHTTCGYTTT*AAARCGAADSCGA*BW_1hr.allmtfs	48.2	0.002	0
CAWTSKATTC	GCAATYGAWTGMA*TKCAWTCRATTGC*BW_2hr.allmtfs	43.5	0	0
NF-kappaB-like	GGGAATTTC*GAAATTCCC*BW_3hr.allmtfs	36.6	0	0
	GCTATCAATHAATTG*CAATTDATTGATAGC*BW_3hr.allmtfs	34.5	0.006	0
	TGCCCAGTGYC*GRCACTGGGCA*BW_48hr.allmtfs	33.6	0.001	0.004
CAWTSKATTC	AKMAATCCAWTS*SAWTGGATTKMT*BW_2hr.allmtfs	32.5	0	0
CAWTSKATTC	AATCGAKTGC*GCAMTCGATT*BW_3hr.allmtfs	32.3	0.002	0.002
	TCAGTCGKCWYCGT*ACGRWGMCGACTGA*BW_2hr.allmtfs	30.7	0	0
AMTCAGT	TGCAMTCARTW*WAYTGAKTGCA*BW_3hr.allmtfs	30.2	0	0
	GAATCAAWATGAAK*MTTCATWTTGATTC*BW_3hr.allmtfs	29.2	0	0
NF-kappaB-like	GGGAAWTTCYTR*YARGAAWTTCCC*BW_1hr.allmtfs	29.2	0	0.001
	AKGAARYTGTTTG*CAAACARYTTCMT*BW_72hr.allmtfs	28.1	0	0
	WKCAATCRART*AYTYGATTGMW*BW_2hr.allmtfs	27.4	0.001	0
	ARTATGCAATM*KATTGCATAYT*BW_24hr.allmtfs	25.5	0	0.002
MTTFA	KnCTTATCnnn_I$MTTFA_01	24.9	0	0
AMTCAGT	TGCAMTCAGT*ACTGAKTGCA*BW_6hr.allmtfs	22.9	0	0
	CRTGTTTGTGRT*AYCACAAACAYG*BW_2hr.allmtfs	22.7	0.009	0.01
TATA	nCTATAAAAR_V$TATA_C	22.1	0	0
NF-kappaB-like	AGAAATTYCMRAA*TTYKGRAATTTCT*BW_48hr.allmtfs	22.1	0.008	0.004
	ATBMKTGGGCT*AGCCCAMKVAT*BW_0hr.allmtfs	21.8	0	0
	CAAGATGARG*CYTCATCTTG*BW_3hr.allmtfs	21.3	0.002	0.002

	TCATWYKCARTCK*MGAYTGMRWATGA*BW_3hr.allmtfs	20.3	0.003	0.006
	ATAWKCTTTTAAK*MTTAAAAGMWTAT*BW_12hr.allmtfs	19.7	0.007	0.002
	GAAWWGGDAAGA*TCTTHCCWWTTC*BW_6hr.allmtfs	19.4	0.008	0.006
TATA	STATAAAWRnnnnnn_V$TATA_01	19.1	0	0
MTTFA/SERPENT/GATA-like	GRCTGARAAGGA*TCCTTYTCAGYC*BW_6hr.allmtfs	19	0	0.001
SERPENT	GCGATAAGAT_SERPEnT_Senger	18.8	0	0
NF-kappaB	nnnnKGGRAAnTCCCn_V$nFKB_Q6_01	18.3	0	0
MTTFA/SERPENT/GATA-like	CTTVTCAGYCGT*ACGRCTGABAAG*BW_3hr.allmtfs	17.8	0	0
GATA	nCWGATAACA_V$GATA1_05	17.5	0	0
	WAAAYATGA*TCATRTTTW*BW_2hr.allmtfs	17.2	0.003	0.008
AMTCAGT	GCAMTCAGTT*AACTGAKTGC*BW_12hr.allmtfs	16.9	0	0
AMTCAGT	CCACTGACTATC*GATAGTCAGTGG*BW_72hr.allmtfs	16.7	0	0
TATA-like	MCMYTTTTATAGC*GCTATAAAARKGK*BW_72hr.allmtfs	16	0	0
	AATCTYTBCGAR*YTCGVARAGATT*BW_48hr.allmtfs	15.4	0	0
GATA	nnWGATAASA_V$GATA2_02	13.9	0	0
	CCRATTATGMTGA*TCAKCATAATYGG*BW_72hr.allmtfs	13.8	0	0.002
AMTCAGT	CACTGAYTATCMC*GKGATARTCAGTG*BW_3hr.allmtfs	12.6	0	0
	ATCAWWMSAATC*GATTSKWWTGAT*BW_1hr.allmtfs	12.5	0	0
	KARCCGAKMTYAT*ATRAKMTCGGYTM*BW_3hr.allmtfs	10.9	0	0
NF-kappaB	SGGRnTTTCC_V$CREL_01	10.8	0.004	0
	CAKYWTCATRGTT*AACYATGAWRMTG*BW_72hr.allmtfs	10.7	0	0.001
NF-kappaB	MA0101_c-REL_REL_	10.5	0.006	0
TCAGTYTT	TTAAARGCTKA*TMAGCYTTTAA*BW_12hr.allmtfs	9.06	0	0
	YAAGTCGCGKKA*TMMCGCGACTTR*BW_3hr.allmtfs	8.22	0	0
	AATCTYTKCGAGT*ACTCGMARAGATT*BW_48hr.allmtfs	6.95	0	0
	ARMCRGTGACR*YGTCACYGKYT*BW_2hr.allmtfs	6.87	0.003	0
	GCKWATGAGTGA*TCACTCATWMGC*BW_6hr.allmtfs	6.28	0.001	0.001
	GATTARAWKWTCA*TGAWMWTYTAATC*BW_0hr.allmtfs	5.79	0.002	0.003
AMTCAGT	CAMTCAGTT*AACTGAKTG*BW_24hr.allmtfs	5.02	0.009	0.01

Set 1

Representative Motif	Motif	Raw Score	P-value for promoter background	P-value for chr 2R
HSF	AGAAnAGAAnAGAAn_I$HSF_02	8.54	0.001	0.003
NF-kappaB	nnnnKGGRAAnTCCCn_V$nFKB_Q6_01	8.15	0	0
	TCGSHTTCGYTTT*AAARCGAADSCGA*BW_1hr.allmtfs	7.78	0	0.002
MTTFA/SERPENT/GATA-like	KCATYCTTATCWT*AWGATAAGRATGM*BW_1hr.allmtfs	7.13	0	0
MTTFA/SERPENT/GATA-like	ATKCTTAKCTTKT*AMAAGMTAAGMAT*BW_2hr.allmtfs	6.85	0	0.003
SPZ1_01	DnnGGRGGGWWnnnn_V$SPZ1_01	5.94	0	0.001
	AAGCATCCWAATY*RATTWGGATGCTT*BW_2hr.allmtfs	5.62	0	0
TATA	STATAAAWRnnnnnn_V$TATA_01	5.57	0.001	0

Set 2

Representative Motif	Motif	Raw Score	P-value for promoter background	P-value for chr 2R
CCARCAGRCCSA	TKYTSGGYCTGYTGG*CCARCAGRCCSARMA*BW_2hr.allmtfs	56.1	0	0
CCARCAGRCCSA	TSGGYCTGCTGGC*GCCAGCAGRCCSA*BW_3hr.allmtfs	52	0	0
CCARCAGRCCSA	KYTCGGYCTGYTGGC*GCCARCAGRCCGARM*BW_3hr.allmtfs	51.3	0	0
CCARCAGRCCSA	YTCGGYYTGYTSGC*GCSARCARRCCGAR*BW_6hr.allmtfs	41.9	0	0
CCARCAGRCCSA	STGCTSGSYCTG*CAGRSCSAGCAS*BW_3hr.allmtfs	40.7	0	0
CCARCAGRCCSA	TCGGYYTGYTSG*CSARCARRCCGA*BW_3hr.allmtfs	40.4	0	0
CCARCAGRCCSA	TSTKCTSGSYCTG*CAGRSCSAGMASA*BW_2hr.allmtfs	38.5	0	0
CCARCAGRCCSA	KTSTKYTSGSTCTG*CAGASCSARMASAM*BW_1hr.allmtfs	37.7	0	0
	TCAGTCGKCWYCGT*ACGRWGMCGACTGA*BW_2hr.allmtfs	36.4	0	0
CCARCAGRCCSA	TSTGYTCGSTCTG*CAGASCGARCASA*BW_1hr.allmtfs	35	0	0
MTTFA/SERPENT/GATA-like	CTTVTCAGYCGT*ACGRCTGABAAG*BW_3hr.allmtfs	21.2	0	0
CCARCAGRCCSA	TCGSCTTGYKGG*CCMRCAAGSCGA*BW_6hr.allmtfs	20.2	0	0
	TCGSHTTCGYTTT*AAARCGAADSCGA*BW_1hr.allmtfs	19	0	0
	TYAYCDTYTTTKT*AMAAARAHGRTRA*BW_3hr.allmtfs	18.5	0	0
	GAATCAAWATGAAK*MTTCATWTTGATTC*BW_3hr.allmtfs	18.1	0	0
CAWTSKATTC	GCAATYGAWTGMA*TKCAWTCRATTGC*BW_2hr.allmtfs	17.5	0	0
AMTCAGT	CACTGAYTATCMC*GKGATARTCAGTG*BW_3hr.allmtfs	16.6	0	0
CAWTSKATTC	AGMAATMSAWTGAA*TTCAWTSKATTKCT*BW_2hr.allmtfs	16.5	0	0
CCARCAGRCCSA	TTCGSCTTGTTSG*CSAACAAGSCGAA*BW_6hr.allmtfs	15.9	0	0
	ARMCRGTGACR*YGTCACYGKYT*BW_2hr.allmtfs	15.1	0	0
MTTFA/SERPENT/GATA-like	ATKCTTAKCTTKT*AMAAGMTAAGMAT*BW_2hr.allmtfs	15	0.001	0.001
CAWTSKATTC	AATCGAKTGC*GCAMTCGATT*BW_3hr.allmtfs	14.6	0	0
CAWTSKATTC	AKMAATCCAWTS*SAWTGGATTKMT*BW_2hr.allmtfs	14.5	0	0
	GCTATCAATHAATTG*CAATTDATTGATAGC*BW_3hr.allmtfs	14	0	0
	KARCCGAKMTYAT*ATRAKMTCGGYTM*BW_3hr.allmtfs	13.8	0	0
	TGCCCAGTGYC*GRCACTGGGCA*BW_48hr.allmtfs	13.5	0	0
	YAAGTCGCGKKA*TMMCGCGACTTR*BW_3hr.allmtfs	13.1	0	0
AMTCAGT	TGCAMTCAGT*ACTGAKTGCA*BW_6hr.allmtfs	12.9	0	0
	ATCATKTTTKT*AMAAAMATGAT*BW_3hr.allmtfs	12.5	0.001	0.001
AMTCAGT	TGCAMTCARTW*WAYTGAKTGCA*BW_3hr.allmtfs	11.9	0	0
	WCATGTTTGTKDT*AHMACAAACATGW*BW_2hr.allmtfs	11.7	0	0.001
AMTCAGT	AATYGAKTGCA*TGCAMTCRATT*BW_2hr.allmtfs	11.3	0	0.001
MTTFA/SERPENT/GATA-like	GAMAAGCAA*TTGCTTKTC*BW_72hr.allmtfs	11.3	0.009	0.007
	CRTGTTTGTGRT*AYCACAAACAYG*BW_2hr.allmtfs	11.2	0	0.002
	AMGCATTTAWTY*RAWTAAATGCKT*BW_48hr.allmtfs	10.9	0	0
	CTCAGTHKTCA*TGAMDACTGAG*BW_3hr.allmtfs	10.8	0	0
	AAATKCTTMTTAY*RTAAKAAGMATTT*BW_2hr.allmtfs	10.6	0	0

MTTFA/SERPENT/GATA-like	GRCTGARAAGGA*TCCTTYTCAGYC*BW_6hr.allmtfs	10.5	0	0
	TCATWYKCARTCK*MGAYTGMRWATGA*BW_3hr.allmtfs	9.78	0.001	0.001
AMTCAGT	ACTGAYWATCMC*GKGATWRTCAGT*BW_3hr.allmtfs	9.67	0	0
	WKCAATCRART*AYTYGATTGMW*BW_2hr.allmtfs	8.34	0.001	0.002
AMTCAGT	CACTGAYWATC*GATWRTCAGTG*BW_3hr.allmtfs	8.26	0	0
	ATCAWWMSAATC*GATTSKWWTGAT*BW_1hr.allmtfs	8.11	0	0
NF-kappaB-like	GGGAATTTC*GAAATTCCC*BW_3hr.allmtfs	8.01	0.002	0.001
	WAAAYATGA*TCATRTTTW*BW_2hr.allmtfs	7.22	0.001	0.001
	KGRCAAGARKAA*TTMYTCTTGYCM*BW_6hr.allmtfs	7.2	0.001	0.004
NF-kappaB-like	GGGAAWTTCYTR*YARGAAWTTCCC*BW_1hr.allmtfs	6.85	0.002	0.004
MTTFA	KnCTTATCnnn_I$MTTFA_01	6.15	0	0
CDPCR1_01	nATCGATCGS_V$CDPCR1_01	6.1	0.005	0.001
MTTFA/SERPENT/GATA-like	TTCTTABYMRT*AYKRVTAAGAA*BW_2hr.allmtfs	5.96	0	0
Thing1-E47_bHLH_	MA0092_Thing1-E47_bHLH_	5.66	0.002	0.003
DORSAL	MA0023_Dorsal_2_REL_	5.62	0.004	0.001
AMTCAGT	CCACTGACTATC*GATAGTCAGTGG*BW_72hr.allmtfs	5.08	0.002	0.002
	GAARACTGAA*TTCAGTYTTC*BW_6hr.allmtfs	5.04	0.004	0.004

Set 4

Representative Motif	Motif	Raw Score	P-value for promoter background	P-value for chr 2R
FREAC2_01	nnAnnGTAAACAAnnn_V$FREAC2_01	6.5	0.006	0
FREAC-4_FORKHEAD_	MA0031_FREAC-4_FORKHEAD_	6.08	0.001	0

Set 6

Representative Motif	Motif	Raw Score	P-value for promoter background	P-value for chr 2R
CCARCAGRCCSA	TKYTSGGYCTGYTGG*CCARCAGRCCSARMA*BW_2hr.allmtfs	7.49	0.003	0.002
CREB_Q4_01	CnnTGACGTMA_V$CREB_Q4_01	5.71	0	0
$ATF_01	CnSTGACGTnnnYC_V$ATF_01	5.69	0	0.001
CCARCAGRCCSA	TCGGYYTGYTSG*CSARCARRCCGA*BW_3hr.allmtfs	5.57	0.009	0.006

Set 9

Representative Motif	Motif	Raw Score	P-value for promoter background	P-value for chr 2R
	CCAAAAAGYWGRC*GYCWRCTTTTTGG*BW_72hr.allmtfs	20.3	0	0
	AAAKTGMATYTGA*TCARATKCAMTTT*BW_72hr.allmtfs	20.1	0	0

	CAKYWTCATRGTT*AACYATGAWRMTG*BW_72hr.allmtfs	19.9	0	0
	TTKACTTRCTTAM*KTAAGYAAGTMAA*BW_72hr.allmtfs	18.5	0	0
	MAAKTGTTTGGT*ACCAAACAMTTK*BW_72hr.allmtfs	18.3	0	0
	AKGAARYTGTTTG*CAAACARYTTCMT*BW_72hr.allmtfs	17.8	0	0
MTTFA/SERPENT/GATA-like	TKTRCTTAWRYAAAT*ATTTRYWTAAGYAMA*BW_72hr.allmtfs	17.5	0	0
	TACYAGMWGGWAAM*KTTWCCWKCTRGTA*BW_72hr.allmtfs	16.6	0	0
	YTTGGCTTKT*AMAAGCCAAR*BW_72hr.allmtfs	15.9	0	0
	CCRATTATGMTGA*TCAKCATAATYGG*BW_72hr.allmtfs	15.6	0	0
	CTTGRCTTGTT*AACAAGYCAAG*BW_72hr.allmtfs	14.3	0	0
	CAATTGGTTTG*CAAACCAATTG*BW_72hr.allmtfs	14.1	0.001	0
	AMAAARAAAAKGA*TCMTTTTYTTTKT*BW_72hr.allmtfs	14	0.006	0.006
TATA-like	MCMYTTTTATAGC*GCTATAAAARKGK*BW_72hr.allmtfs	13.9	0	0
	AGWWARAACCATG*CATGGTTYTWWCT*BW_72hr.allmtfs	13	0	0
MTTFA/SERPENT/GATA-like	GAMAAGCAA*TTGCTTKTC*BW_72hr.allmtfs	12.7	0	0
	TAGWWARAACMATS*SATKGTTYTWWCTA*BW_72hr.allmtfs	12.1	0	0
TATA	nCTATAAAAR_V$TATA_C	9.4	0	0
	GCAWGTCAA*TTGACWTGC*BW_72hr.allmtfs	7.13	0	0
	TMWAKCWACA*TGTWGMTWKA*BW_72hr.allmtfs	5.82	0	0
TATA	STATAAAWRnnnnnn_V$TATA_01	5.03	0.004	0

Set 10

Representative Motif	Motif	Raw Score	P-value for promoter background	P-value for chr 2R
MTTFA/SERPENT/GATA-like	TKTRCTTAWRYAAAT*ATTTRYWTAAGYAMA*BW_72hr.allmtfs	8.43	0.009	0.01
TATA	STATAAAWRnnnnnn_V$TATA_01	6.21	0.002	0
GATA	nnWGATAASA_V$GATA2_02	5.68	0	0

Set 11

Representative Motif	Motif	Raw Score	P-value for promoter background	P-value for chr 2R
	TTYKRAAATATYTC*GARATATTTYMRAA*BW_48hr.allmtfs	10.1	0.001	0.002
	AARTAARAATTTA*TAAATTYTTAYTT*BW_48hr.allmtfs	9.55	0.004	0.01
	GAWRATTTAATGA*TCATTAAATYWTC*BW_48hr.allmtfs	6.91	0	0.006
	ARRTARTAYTTT*AAARTAYTAYYT*BW_48hr.allmtfs	6.71	0.002	0.001
	ARTTTTMAAMAKA*TMTKTTKAAAAYT*BW_48hr.allmtfs	6.42	0.01	0.004
NF-kappaB-like	SGGRnTTTCC_V$CREL_01	5.09	0.006	0.004
NF-kappaB-like	MA0101_c-REL_REL_	5.05	0.009	0.006

Set 12

Representative Motif	Motif	Raw Score	P-value for promoter background	P-value for chr 2R
CCARCAGRCCSA	KYTCGGYCTGYTGGC*GCCARCAGRCCGARM*BW_3hr.allmtfs	27.8	0	0
CCARCAGRCCSA	YTCGGYYTGYTSGC*GCSARCARRCCGAR*BW_6hr.allmtfs	25.7	0	0
CCARCAGRCCSA	TSGGYCTGCTGGC*GCCAGCAGRCCSA*BW_3hr.allmtfs	24.1	0	0
CCARCAGRCCSA	TCGGYYTGYTSG*CSARCARRCCGA*BW_3hr.allmtfs	22.1	0	0
CCARCAGRCCSA	STGCTSGSYCTG*CAGRSCSAGCAS*BW_3hr.allmtfs	21.8	0	0
CCARCAGRCCSA	TCGSCTTGYKGG*CCMRCAAGSCGA*BW_6hr.allmtfs	21.4	0	0
CCARCAGRCCSA	TTCGSCTTGTTSG*CSAACAAGSCGAA*BW_6hr.allmtfs	20.6	0	0
	GGRCAAGRGGAA*TTCCYCTTGYCC*BW_0hr.allmtfs	19.2	0	0
NF-kappaB-like	GGAAAWTYCCA*TGGRAWTTTCC*BW_3hr.allmtfs	17.3	0	0.001
CCARCAGRCCSA	GTTKGSATTGTCC*GGACAATSCMAAC*BW_48hr.allmtfs	16.7	0	0
AMTCAGT	TGCAATCRRTTK*MAAYYGATTGCA*BW_12hr.allmtfs	16.5	0	0.001
	AYTGAAAADRA*TYHTTTTCART*BW_12hr.allmtfs	16.4	0.002	0.001
CCARCAGRCCSA	TKYTSGGYCTGYTGG*CCARCAGRCCSARMA*BW_2hr.allmtfs	16.3	0.01	0.007
TCAKTYTT	TGAARACTGAAA*TTTCAGTYTTCA*BW_12hr.allmtfs	16.1	0.007	0.009
	KGRCAAGARKAA*TTMYTCTTGYCM*BW_6hr.allmtfs	15.7	0	0
TCAGTYTT	WTGAARAMTDAAA*TTTHAKTYTTCAW*BW_24hr.allmtfs	15.5	0.01	0.009
	ATWWTYMKWTTCT*AGAAWMKRAWWAT*BW_6hr.allmtfs	13.6	0.006	0.001
CCARCAGRCCSA	CCYCTTGYCC*GGRCAAGRGG*BW_12hr.allmtfs	12.6	0.001	0
	YGAATAAATAAAR*YTTTATTTATTCR*BW_12hr.allmtfs	12.2	0.001	0.002
NF-kappaB-like	GGGAATTTC*GAAATTCCC*BW_3hr.allmtfs	11.4	0	0
CCARCAGRCCSA	TSTKCTSGSYCTG*CAGRSCSAGMASA*BW_2hr.allmtfs	11.4	0.003	0.005
	TCATWYKCARTCK*MGAYTGMRWATGA*BW_3hr.allmtfs	11.3	0	0.002
	ARTTTTMAAMAKA*TMTKTTKAAAAYT*BW_48hr.allmtfs	11	0.003	0.001
AMTCAGT	TGCAMTCAGT*ACTGAKTGCA*BW_6hr.allmtfs	10.9	0	0
	CGTWCTTARTGC*GCAYTAAGWACG*BW_6hr.allmtfs	10.8	0	0
	GAAWWGGDAAGA*TCTTHCCWWTTC*BW_6hr.allmtfs	10.6	0.001	0
MTTFA/SERPENT/GATA-like	GRCTGARAAGGA*TCCTTYTCAGYC*BW_6hr.allmtfs	10.5	0.001	0
	ATAWKCTTTTAAK*MTTAAAAGMWTAT*BW_12hr.allmtfs	9.95	0.002	0.001
AMTCAGT	TGCAMTCARTW*WAYTGAKTGCA*BW_3hr.allmtfs	9.84	0	0.001
	CAATCWAMATGC*GCATKTWGATTG*BW_24hr.allmtfs	9.04	0	0.002
TCAGTYTT	AARAMTGA*TCAKTYTT*BW_12hr.allmtfs	8.87	0	0
MTTFA	KnCTTATCnnn_I$MTTFA_01	8.49	0	0
SERPENT	GCGATAAGAT_SERPEnT_Senger	8.12	0	0
	ATBMKTGGGCT*AGCCCAMKVAT*BW_0hr.allmtfs	7.9	0.002	0.001
	GRAACTCTAC*GTAGAGTTYC*BW_0hr.allmtfs	7.4	0	0
	GCKWATGAGTGA*TCACTCATWMGC*BW_6hr.allmtfs	7.08	0	0
AMTCAGT	GCAMTCAGTT*AACTGAKTGC*BW_12hr.allmtfs	6.99	0.003	0.003
GATA	nnWGATAASA_V$GATA2_02	6.75	0	0

TCAGTYTT	TTAAARGCTKA*TMAGCYTTTAA*BW_12hr.allmtfs	6.43	0.001	0.001
	AATCTYTBCGAR*YTCGVARAGATT*BW_48hr.allmtfs	6.41	0.001	0.001
	GATTARAWKWTCA*TGAWMWTYTAATC*BW_0hr.allmtfs	6.17	0	0
GATA	nCWGATAACA_V$GATA1_05	6.1	0.001	0
	GTCCTYGGTG*CACCRAGGAC*BW_0hr.allmtfs	5.94	0.006	0.004
AMTCAGT	CCACTGACTATC*GATAGTCAGTGG*BW_72hr.allmtfs	5.75	0	0
	TYATTCATTCGM*KCGAATGAATRA*BW_12hr.allmtfs	5.72	0.001	0
	ATADGMATTTMA*TKAAATKCHTAT*BW_6hr.allmtfs	5.71	0.008	0.01
	HATTYATTCG*CGAATRAATD*BW_6hr.allmtfs	5.09	0	0

Set 14

Representative Motif	Motif	Raw Score	P-value for promoter background	P-value for chr 2R
CCARCAGRCCSA	KYTCGGYCTGYTGGC*GCCARCAGRCCGARM*BW_3hr.allmtfs	14.3	0.001	0
CCARCAGRCCSA	TSGGYCTGCTGGC*GCCAGCAGRCCSA*BW_3hr.allmtfs	13.8	0.003	0.001
CCARCAGRCCSA	YTCGGYYTGYTSGC*GCSARCARRCCGAR*BW_6hr.allmtfs	13.7	0.001	0
NF-kappaB-like	GGRRAATYCCAA*TTGGRATTYYCC*BW_1hr.allmtfs	12.4	0.001	0
	GCTATCAATHAATTG*CAATTDATTGATAGC*BW_3hr.allmtfs	10.8	0.001	0.001
NF-kappaB-like	GGAAAWTYCCA*TGGRAWTTTCC*BW_3hr.allmtfs	9.44	0	0
AMTCAGT	AATYGAKTGCA*TGCAMTCRATT*BW_2hr.allmtfs	8.99	0.001	0.008
N-MYC_bHLH-ZIP_	MA0104_n-MYC_bHLH-ZIP_	8.59	0	0
CCARCAGRCCSA	KTSTKYTSGSTCTG*CAGASCSARMASAM*BW_1hr.allmtfs	8.53	0.005	0.01
NF-kappaB-like	AGAAATTYCMRAA*TTYKGRAATTTCT*BW_48hr.allmtfs	8.41	0.002	0.001
CAWTSKATTC	GCAATYGAWTGMA*TKCAWTCRATTGC*BW_2hr.allmtfs	8.39	0.008	0.008
CAWTSKATTC	AATCGAKTGC*GCAMTCGATT*BW_3hr.allmtfs	8.38	0.004	0.002
MA0004_ARnT_bHLH_	MA0004_ARnT_bHLH_	8.31	0	0
DORSAL	HGRGAAAAnCV_I$DL_02	8.23	0.001	0.005
MYC	CACGTGS_V$MYC_Q2	7.75	0	0
CCARCAGRCCSA	TSTKCTSGSYCTG*CAGRSCSAGMASA*BW_2hr.allmtfs	7.03	0.009	0.01
NF-kappaB-like	GGGAATTTC*GAAATTCCC*BW_3hr.allmtfs	6.85	0.003	0.003
	ARTATGCAATM*KATTGCATAYT*BW_24hr.allmtfs	6.79	0	0.002
AMTCAGT	TGCAMTCARTW*WAYTGAKTGCA*BW_3hr.allmtfs	6.72	0.002	0.002
	AATCTYTBCGAR*YTCGVARAGATT*BW_48hr.allmtfs	6.69	0	0
NF-kappaB-like	GGGAAWTTCYTR*YARGAAWTTCCC*BW_1hr.allmtfs	6.53	0.002	0.001
	AATCTYTKCGAGT*ACTCGMARAGATT*BW_48hr.allmtfs	5.82	0	0
USF	nCACGTGn_V$USF_C	5.47	0	0
	YAAGTCGCGKKA*TMMCGCGACTTR*BW_3hr.allmtfs	5.28	0.001	0
USF_bHLH-ZIP_	MA0093_USF_bHLH-ZIP_	5.2	0	0
AMTCAGT	TGCAMTCAGT*ACTGAKTGCA*BW_6hr.allmtfs	5.07	0.006	0.004
