# Supplementary material for: Genome-wide gene expression in response to parasitoid attack in Drosophila
Source: Genome Biol. 2005 Oct 31;6(11):R94. doi: 10.1186/gb-2005-6-11-r94 (PMC1297650; doi:10.1186/gb-2005-6-11-r94)
Supplement: Additional data file 4 — A diagrammatic representation of the degenerate motifs of the putative TFBMs. The size of the letters represents the likelihood of its occurrence at each position in the sequence. [file gb-2005-6-11-r94-S4.pdf]

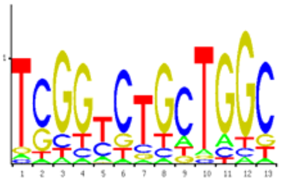

CCARCAGRCCSA:
TSGGYCTGCTGGC*GCCAGCAGRCCSA*BW_3hr.allmtfs

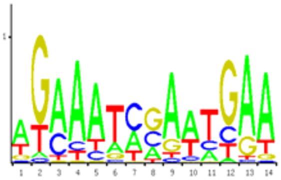

CAWTSKATTC:
AGMAATMSAWTGAA*TTCAWTSKATTKCT*BW_2hr.allmtfs

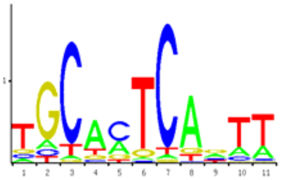

AMTCAGT:
TGCAMTCARTW*WAYTGAKTGCA*BW_3hr.allmtfs

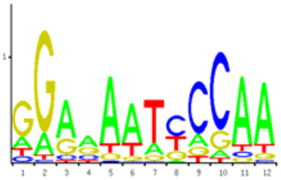

NF-kappaB-like:
GGRRAATYCCAA*TTGGRATTYYCC*BW_1hr.allmtfs

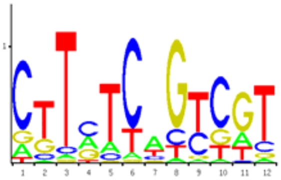

MTTFA/SERPENT/GATA-like:
CTTVTCAGYCGT*ACGRCTGABAAG*BW_3hr.allmtfs

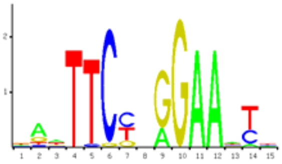

STAT:
nAWTTCYnGGAAnYn_V$STAT5A_01

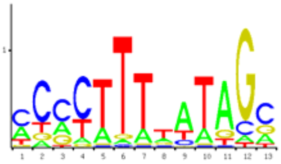

TATA-like:
MCMYTTTTATAGC*GCTATAAAARKGK*BW_72hr.allmtfs

Motif logos for TF binding sites
Wertheim et al
Supplemental Material, Figure 2
